# Lenvatinib halts aortic aneurysm growth by restoring smooth muscle cell contractility

**DOI:** 10.1172/jci.insight.140364

**Published:** 2021-08-09

**Authors:** Albert Busch, Jessica Pauli, Greg Winski, Sonja Bleichert, Ekaterina Chernogubova, Susanne Metschl, Hanna Winter, Matthias Trenner, Armin Wiegering, Christoph Otto, Johannes Fischer, Judith Reiser, Julia Werner, Joy Roy, Christine Brostjan, Christoph Knappich, Hans-Henning Eckstein, Valentina Paloschi, Lars Maegdefessel

**Affiliations:** 1 Department for Vascular and Endovascular Surgery, Klinikum rechts der Isar, Technical University of Munich, Munich, Germany.; 2 German Center for Cardiovascular Research (DZHK), Berlin, Germany.; 3 Division of Vascular and Endovascular Surgery, Department of Visceral, Thoracic and Vascular Surgery, Medical Faculty, Carl Gustav Carus and University Hospital Carl Gustav Carus Dresden, Technische Universität Dresden, Dresden, Germany.; 4 Molecular Vascular Medicine Unit, Department of Medicine, Karolinska Institutet, Stockholm, Sweden.; 5 Division of Vascular Surgery, Department of General Surgery, Medical University of Vienna, Vienna General Hospital, Vienna, Austria.; 6 Department of General, Visceral, Transplantation, Vascular & Pediatric Surgery (Department of Surgery I), University Hospital Würzburg, Würzburg, Germany.; 7 Centre of Preclinical Research, Klinikum rechts der Isar, Technical University of Munich, Munich, Germany.; 8 Department of Molecular Medicine and Surgery, Karolinska Institutet, Stockholm, Sweden.

**Keywords:** Vascular Biology, Drug therapy

## Abstract

Abdominal aortic aneurysm (AAA) is a disease with high morbidity and mortality, especially when ruptured. The rationale of this study was to evaluate the repurposing of lenvatinib, a multi–tyrosine kinase inhibitor, in limiting experimental AAA growth targeting vascular smooth muscle cells (VSMCs) and angiogenesis. We applied systemic and local lenvatinib treatment to elastase-induced murine aortic aneurysms, and RNA profiling identified myosin heavy chain 11 (*Myh11*) as the most deregulated transcript. Daily oral treatment substantially reduced aneurysm formation in 2 independent mouse models. In addition, a large animal aneurysm model in hypercholesterolemic low-density lipoprotein receptor–knockout (*LDLR^–/–^*) Yucatan minipigs was applied to endovascularly deliver lenvatinib via drug-eluting balloons (DEBs). Here, a single local endovascular delivery blocked AAA progression successfully compared with a DEB-delivered control treatment. Reduced VSMC proliferation and a restored contractile phenotype were observed in animal tissues (murine and porcine), as well as AAA patient-derived cells. Apart from increasing MYH11 levels, lenvatinib reduced downstream ERK signaling. Hence, lenvatinib is a promising therapy to limit aortic aneurysm expansion upon local endovascular delivery. The tyrosine kinase inhibitor was able to positively affect pathways of key relevance to human AAA disease, even in a potentially new local delivery using DEBs.

## Graphical abstract

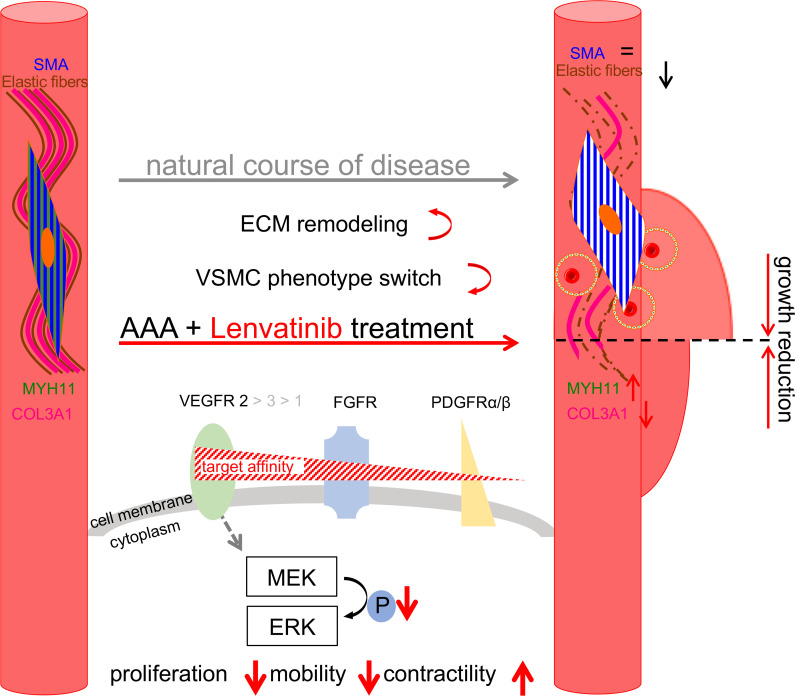

## Introduction

AAA is a diameter enlargement of the infrarenal aorta by 1.5-fold or bigger. The susceptible population has an age-dependent prevalence of 3%–11% (1). Due to the risk of rupture, aneurysm disease is among the leading causes of death in Western countries. Risk factors include arterial hypertension, hyperlipidemia, male sex, family history, and smoking. International guidelines recommend treatment upon a threshold diameter of 5.5 cm, and modern treatment favors endovascular over open repair, despite similar long-term results (2).

The pathogenesis of AAA is not yet fully understood, and the mechanisms triggering disease development and progression remain unclear. In particular, the initial event and site-specific occurrence of aneurysm formation are largely elusive, prompting recent interest into better experimental models and research in general (1). Some crucial mechanisms have been well characterized, including local wall stress alteration, enzymatic and mechanical intraluminal thrombus formation, proteolytic imbalance, and a humoral immune answer as a possible repair or healing mechanism (3). Moreover, phenotypic changes in VSMCs and the hypoxic micromilieu that involves angiogenesis in the aortic media have gained some attention and experimental therapies aimed at addressing them (4).

Lately, we were able to demonstrate a positive effect on halting AAA growth by RNA interference targeting the long noncoding RNA H19, which represses HIF1α signaling and its apoptotic effect on vascular smooth muscle cells (VSMCs) during aneurysm progression (5). Others have demonstrated that influencing VSMC signaling and plasticity alters aneurysm growth in experimental mouse models (6). However, small animal models remain limited in their ability to mimic human disease, thus making it impossible to test local and site-specific delivery of therapeutics directly into the aortic wall (7).

To date, several treatment strategies using established drugs with other indications were successfully tested in rodent AAA models (e.g., doxycycline or sartans). Some have even entered small-scale clinical trials but failed to limit aneurysm growth and rupture risk in patients (8). Still, drug repurposing has emerged as a new way to limit cost and time for compound development, by expanding the spectrum of approved medications to new or additional applications (9). The multi–tyrosine kinase inhibitor (TKI) lenvatinib is used to block angiogenesis in cancer therapy (i.e., thyroid cancer) (10). Its inhibition of vascular endothelial growth factor receptor 2 (VEGFR2) makes it a suitable repurposed candidate to potentially limit AAA progression. Thus, we decided to investigate the potential of systemic and endovascular local lenvatinib delivery to halt aneurysm growth in experimental murine models, primary human AAA patient-derived cells, and a novel preclinical Yucatan *LDLR^–/–^* minipig AAA model.

## Results

### Lenvatinib halts aneurysm growth in murine AAA and induces myosin heavy chain 11 expression.

**Figure 1:**
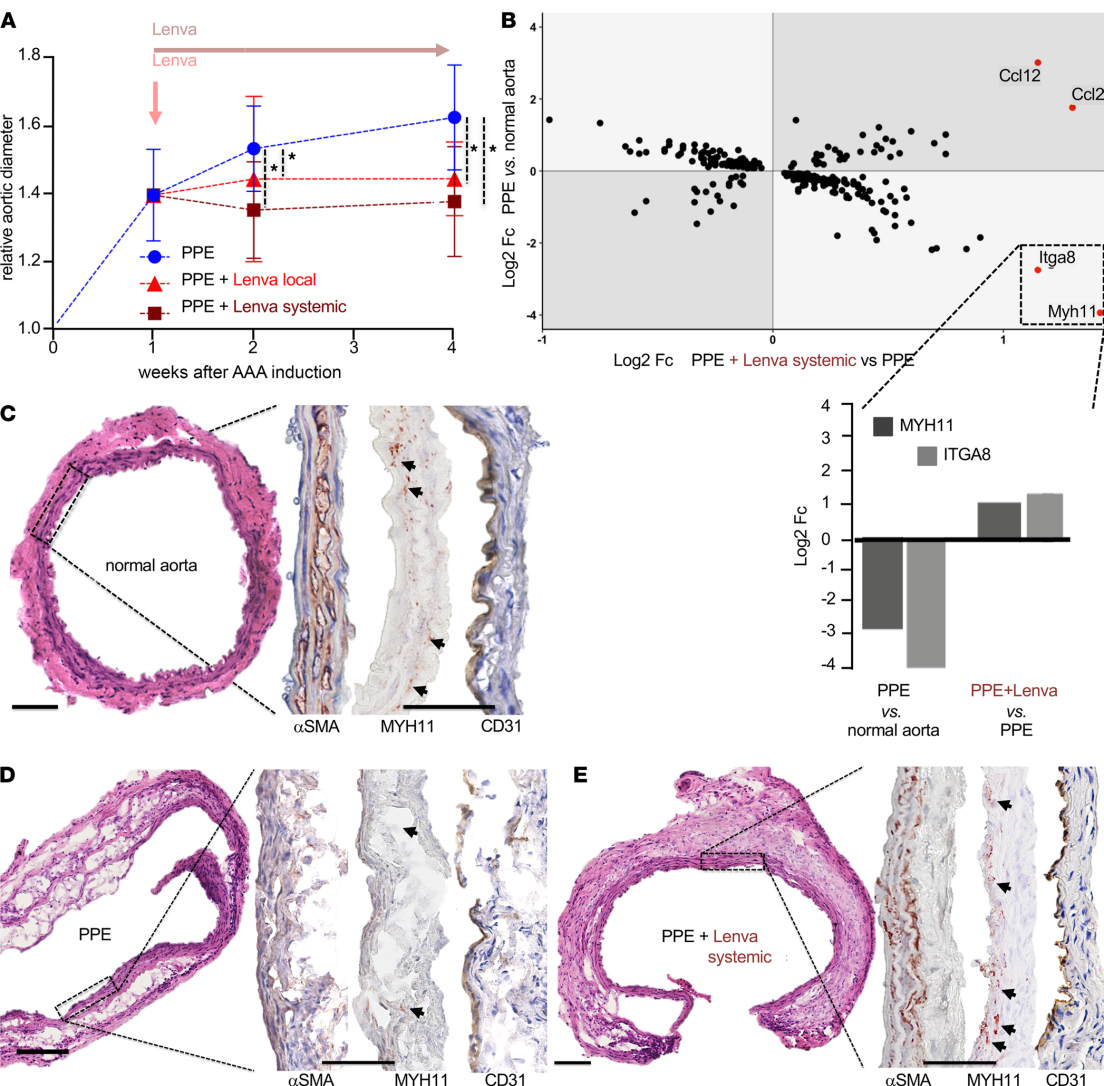
Effects of lenvatinib treatment in vivo. (**A**) Two-way repeated measures ANOVA revealed significant effects of time, treatment, and the interaction of time and treatment, on aneurysm diameter in the PPE model. Both systemic oral and local endovascular lenvatinib treatment, starting 7 days after PPE-AAA induction, completely blocked aortic dilatation (local: red, *n* = 5; systemic: dark red, *n* = 7) versus sham-treated PPE-AAA mice (blue, *n* = 11) assessed by B-mode ultrasound imaging (*P < 0.05; displayed relative growth compared with baseline aortic diameter; corresponding absolute diameters shown in Supplemental Figure 1A; *P* value calculations and detailed measurements in Supplemental Table 1; PPE AAA induction at day 0). (**B**) Double fold change (Fc) plot depicts analysis of RNA profiling (mouse transcriptome array; MTA_1.0). Areas with light-gray background represent genes regulated in opposite directions (rescue effect) upon lenvatinib treatment. *Myh11* displayed the most pronounced changes comparing log_2_ Fc of gene expression in PPE-induced AAA (PPE) versus control and PPE-induced AAA + lenvatinib (PPE+Lenva systemic) versus PPE. The bar chart highlights and indicates direction (up or down) of the most prominent changes in gene expression from the Fc plot (MTA analysis: *n* = 6 PPE; *n* = 7 PPE+Lenva systemic; *n* = 5 untreated so-called normal aorta). (**C**–**E**) H&E staining reveals a disrupted media with pronounced cellular infiltrates in PPE-AAA (**D**) compared with control aorta (**C**). (**E**) Upon systemic lenvatinib treatment, cellular infiltrates were reduced, with a more compact media, a thickened adventitia, and marked fibrosis. In all 3 groups αSMA staining was positive, emphasizing a more linear organized media in control aortas and upon lenvatinib treatment. MYH11 was absent in PPE and highly positive in the media of lenvatinib-treated mice (dark red stain, highlighted with arrows). CD31^+^ cells (brown stain) indicate intact endothelial lining in all 3 groups, but only in PPE few positive cells are present in the media and adventitia (original magnification 10×/40×; scale bar: 100 μm). αSMA, α–smooth muscle actin.

**Figure 2:**
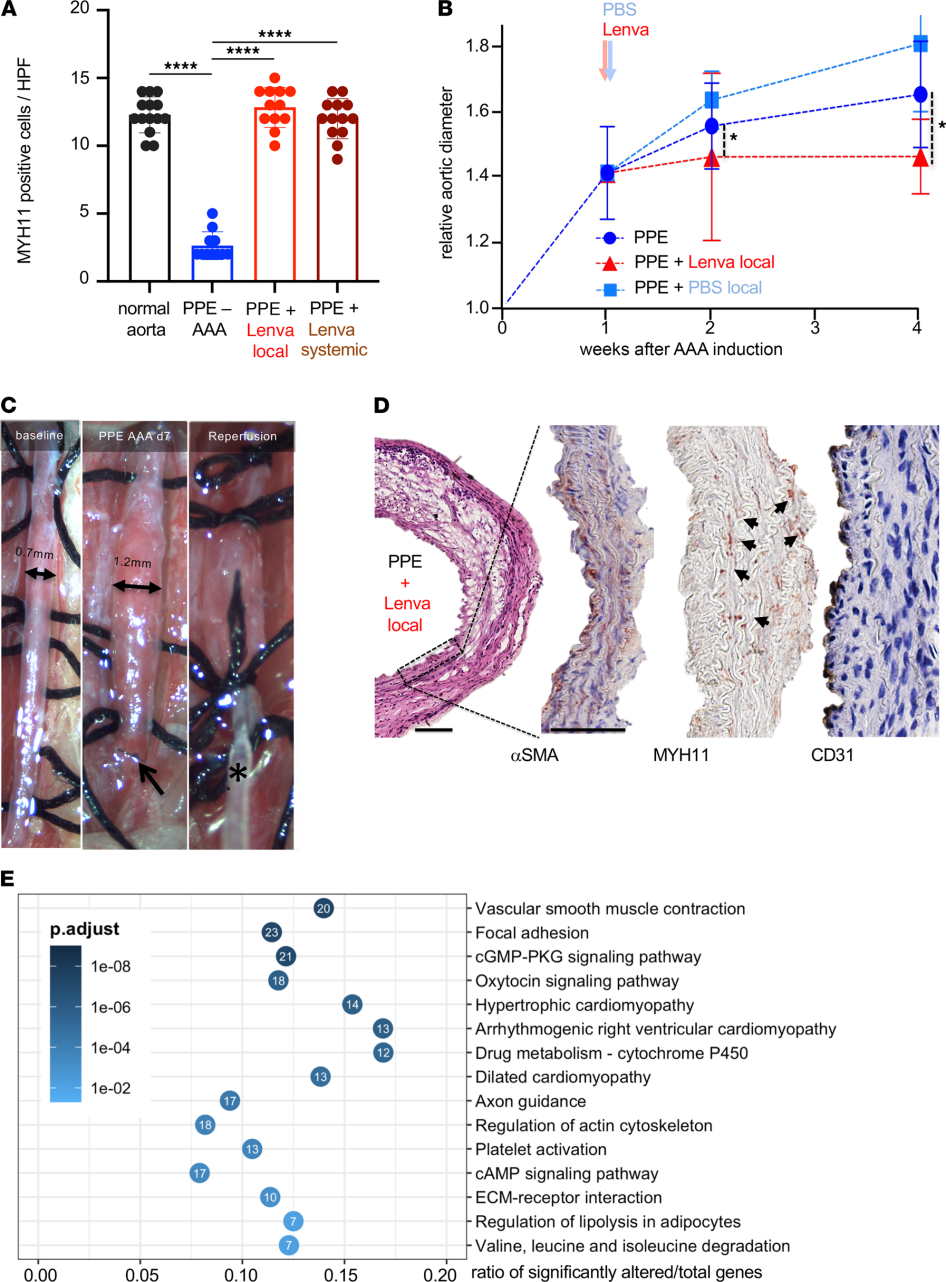
Lenvatinib rescues SMC contractility in vivo. (**A**) Quantification using high-power field (HPF) analysis of cell counts for MYH11-positive cells. Systemic and local lenvatinib treatment restored MYH11 positivity in the media of PPE-AAA (****P < 0.0001; 1-tailed *t* test). (**B**) Ultrasound measurements of aortic diameter are shown for the 3 treatment groups (PPE: *n* = 11, PPE + Lenva local: *n* = 5 and sham reoperation, PPE + PBS local: *n* = 3) during a timeline of 4 weeks. Relative aortic diameter is calculated in relation to baseline diameter at day 0 (PPE-AAA induction). (*P < 0.05; absolute diameters are shown in Supplemental Figure 1A; *P* value calculations and detailed measurements in Supplemental Table 1; 2-way repeated measures ANOVA.) (**C**) The established AAA (arrow indicates the previous aortic suture from the initial procedure) was prepared from the retroperitoneum, and a local perfusion with lenvatinib was performed (asterisk indicates the perfusion catheter). (**D**) Immunohistochemical analysis of AAAs treated locally with lenvatinib showing αSMA, MYH11 (highlighted by arrows), and CD31 (magnification 10×/40×; scale bar: 100 μm). (**E**) KEGG pathway gene overrepresentation analysis of significantly downregulated genes (adjusted *P* < 0.05, Fc < –2) at day 7 after PPE-AAA compared with sham (saline) controls, showing baseline transcriptomic characteristics of the model at the time point of lenvatinib treatment initiation. Top 15 significantly affected pathways are shown (adjusted *P* value is given in shades of blue). KEGG, Kyoto Encyclopedia of Genes and Genomes.

Mice with porcine pancreatic elastase–induced (PPE-induced) AAAs were treated daily with lenvatinib (body weight adjusted) via oral gavage as previously established by others (11). Treatment started 7 days after aneurysm induction when a small AAA had already developed upon PPE instillation (7). Lenvatinib completely halted aneurysm growth over the following 3 weeks until sacrifice (fold increase of dilation in Figure 1A, with absolute values in Supplemental Figure 1A; supplemental material available online with this article; https://doi.org/10.1172/jci.insight.140364DS1; detailed weekly measurements in Supplemental Table 1). Aortic tissue RNA profiling using the mouse transcriptome array (*MTA_1.0*) analyzing untreated animals (normal aorta), PPE-induced AAAs (PPE), and lenvatinib-treated animals (PPE + Lenva systemic) revealed myosin heavy chain 11 (*Myh11*) as the most significantly deregulated transcript (Figure 1B, Figure 2E, and Supplemental Figure 4, A and B) when comparing all subgroups (downregulated in PPE vs. normal aorta; upregulated in PPE + Lenva systemic vs. PPE).

MYH11 is an established marker for the contractile phenotype of VSMCs and known to be decreased in AAA compared with nonaneurysmal controls (12). Immunohistochemical analysis revealed substantially higher levels of MYH11 present in the media of normal aortas, as well as in lenvatinib-treated PPE-AAA; however, it was completely absent in regular PPE-induced AAAs (Figure 1, C–E; and Supplemental Figure 2, with quantification in Figure 2A). CD31^+^ cells indicated angiogenic activity in the media and endothelium of PPE-AAAs, whereas solely the endothelium was marked in control and lenvatinib-treated animals (Figure 1, C–E, and Supplemental Figure 1B). Thus, gene expression and immunohistochemistry, along with basic morphologic analysis using H&E suggested a phenotypic aortic wall restoration upon lenvatinib treatment with reduced angiogenesis, reestablished MYH11 levels, and signs of amplified fibrosis. Body weight and basic blood laboratory analysis were unchanged between treatment and control groups (Supplemental Table 1). All established lenvatinib target receptors, especially *Vegfr2*, were deregulated in PPE-induced AAAs (Supplemental Figure 1C) (13).

Similarly, lenvatinib (delivered via daily oral gavage) reduced aortic diameter and volume in a confirmatory study using a second murine model of AAA disease, angiotensin II (AngII) infusion in *ApoE^–/–^* mice (Supplemental Figure 3). In line with our study in PPE-induced AAA, treatment in this model was once more started when aortic dilatation already occurred at day 4 upon induction (detailed weekly measurements in Supplemental Table 1).

In addition, we tested the potential benefit of local delivery of lenvatinib to the aorta, again 7 days after experimental aneurysm induction was performed in the PPE model (Figure 2, B and C). Reintervention and local perfusion of the established small AAA resulted in a significant diameter reduction compared with sham-intervened mice with AAA after 28 days (fold increase of diameters in Figure 2B; absolute values in Supplemental Figure 1D; detailed weekly measurements are summarized in Supplemental Table 1). Both systemic oral and local lenvatinib treatment had analogous effects on completely halting AAA progression. A similar phenotypic salvage, with a more contained aortic structure and enhanced MYH11 levels after local lenvatinib treatment, could also be observed (Figure 2, A and D). These effects were not detectable after local reperfusion with PBS, which was intended as a surgical (sham) control experiment (Supplemental Figure 1, D and E, and Supplemental Figure 4B).

### Lenvatinib affects VSMC plasticity in AAA patient-derived cells.

**Figure 3:**
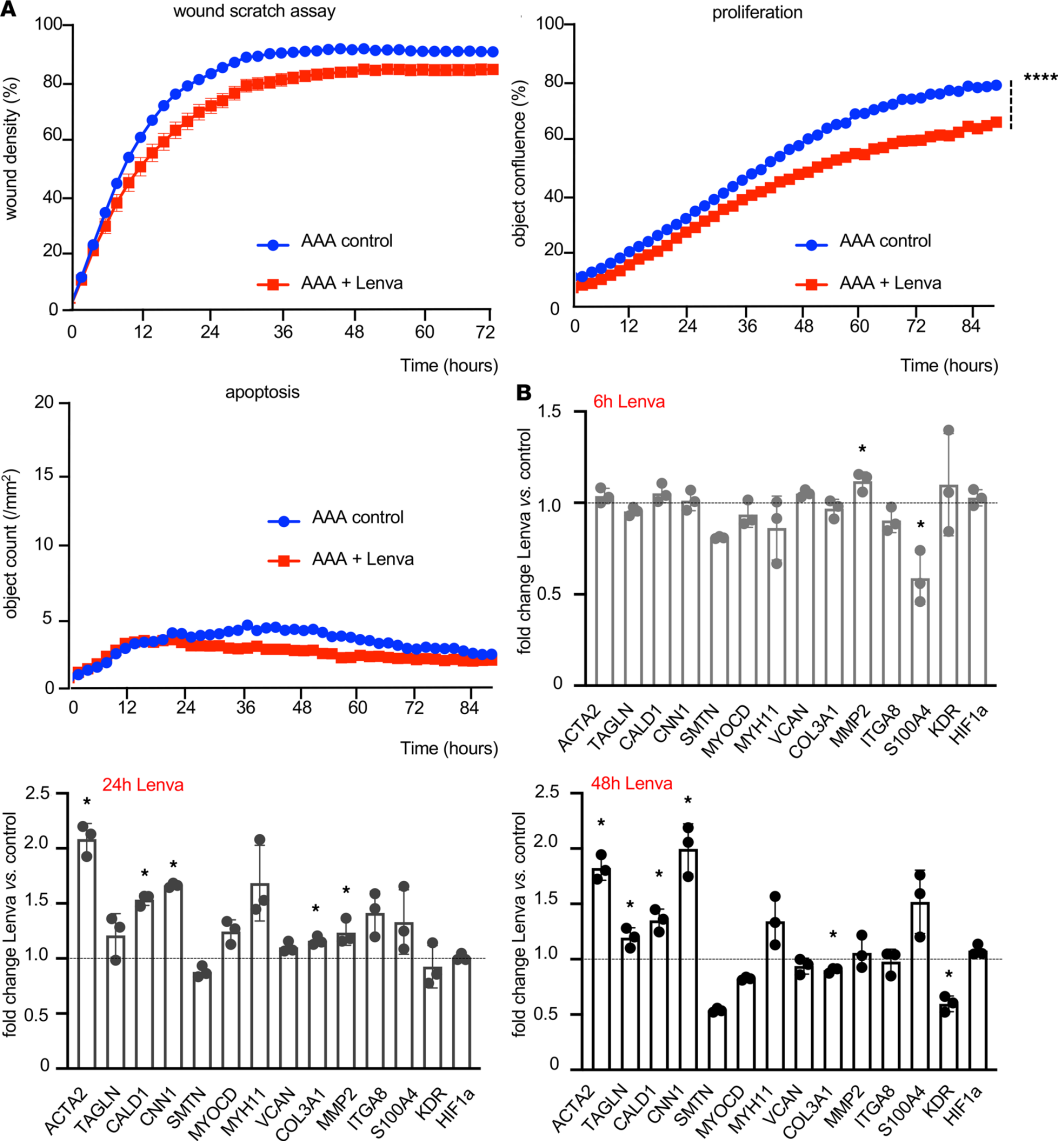
Lenvatinib effect in AAA patient-derived SMCs. (**A**) Live-cell imaging (for up to 3 days) detected a reduced VSMC migration (wound scratch assay) upon lenvatinib stimulation (0.1 μM) versus control (vehicle: DMSO, 0.1%). Lenvatinib significantly decreased VSMC proliferation but did not change survival and cell apoptosis (indicated by Caspase3/7-positive cells). (**B**) Gene expression changes following lenvatinib treatment (data are presented as mean of biological triplicates + SEM, *P < 0.05; ****P < 0.0001; 1-tailed *t* test).

To enhance translation of our finding, primary VSMC culture derived from 3 different AAA patients was obtained (age: 71 ± 3 years; diameter: 65 ± 5 mm; Figure 3 and Supplemental Figures 5–7). First, we discovered a dose-dependent antiproliferative and antimigrative effect upon lenvatinib treatment in vitro on healthy human donor aortic smooth muscle cells (Supplemental Figure 6C). Then, cell mobility (wound scratch assay) and cell proliferation were also assessed in patient-derived AAA-VSMCs. Here, lenvatinib treatment significantly reduced both mobility and proliferation compared with cells treated with vehicle (DMSO) only (Figure 3). Apoptosis appeared at low rates in general and was not affected by lenvatinib treatment (Figure 3A). However, lenvatinib did significantly reduce apoptosis in VSMCs from healthy donors (Supplemental Figure 7A, right panel). RNA profiling identified a subset of genes typically involved in maintaining contractile VSMC function as significantly increased upon lenvatinib treatment in a time-dependent manner (Figure 3B; Supplemental Figures 5, 6, and 7B; and Supplemental Table 2). VEGFR2 (Kdr), a direct target of lenvatinib, was significantly downregulated in all in vitro experiments after 48 hours of treatment. AAA patient-derived cells displayed an accelerated reactivity — based on more substantial changes in gene expression — to lenvatinib in all assays (Figure 3 and Supplemental Figures 5–7). Overall cell integrity of the patient-derived VSMCs was confirmed by cytoskeleton/contractile filament staining (Supplemental Figure 8).

### Lenvatinib promotes a contractile VSMC phenotype and reduces ERK signaling.

**Figure 4:**
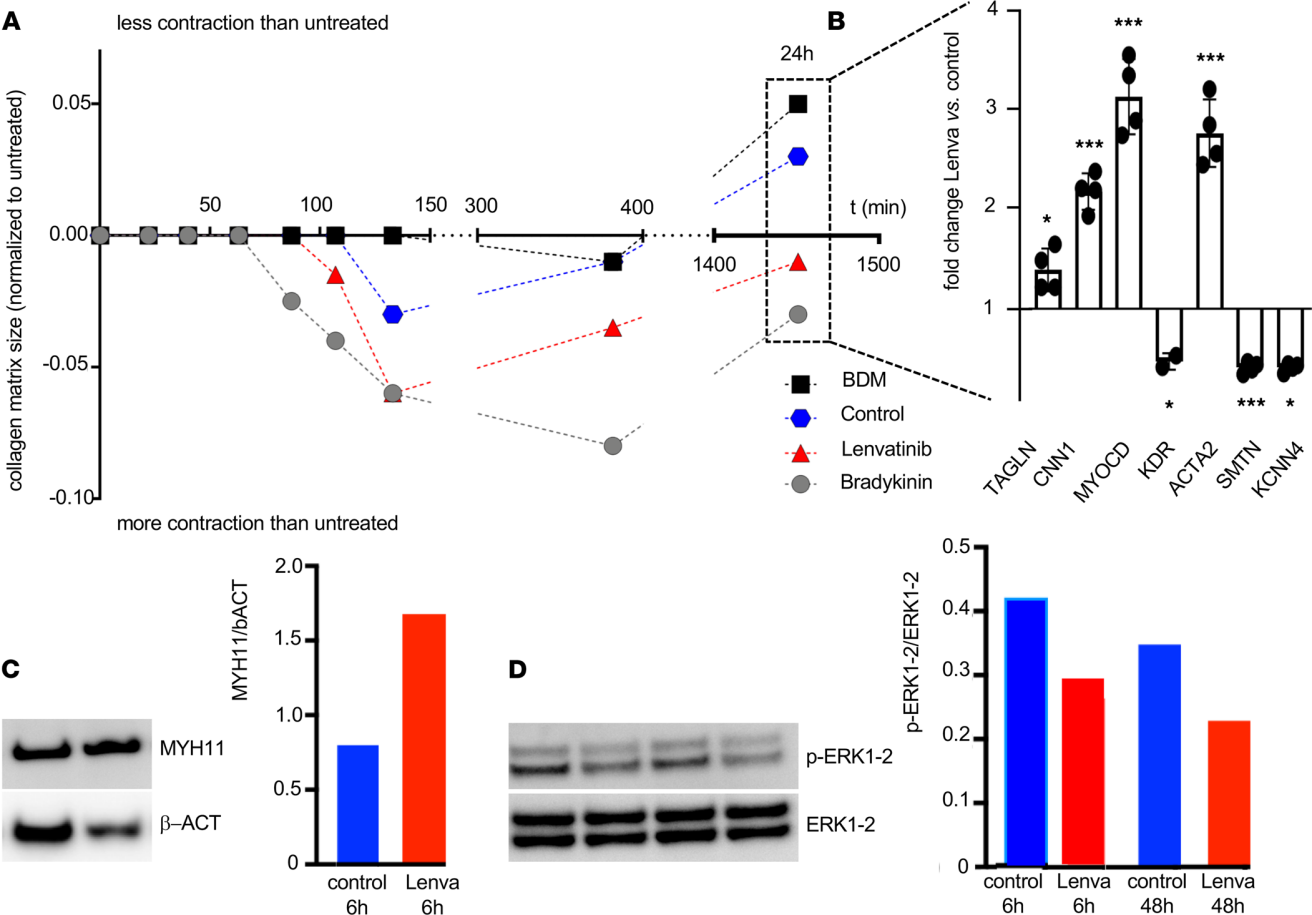
Lenvatinib triggers SMC contractility in vitro. (**A**) Collagen-based contractility assay to assess AAA patient-derived SMCs’ contraction in presence of vasoactive hormone bradykinin (gray), contraction inhibitor BDM (black), lenvatinib (red), and control treatment DMSO (blue). (**B**) Gene expression analysis performed on cells embedded in the collagen matrix after 24 hours showing the lenvatinib treatment effect in comparison to the control-exposed cells. (Data are presented as mean + SEM; *P < 0.05, ***P < 0.001.) (**C**) Western blot analysis for MYH11 and (**D**) phosphorylated ERK1-2 in control treated (blue) and lenvatinib-treated cells (red). Protein bands were quantified and normalized to β-actin and total ERK1-2, respectively. Complete Western blot (WB) images are shown in Supplemental Figure 9A and Supplemental Figure 10A.

In a collagen-based contractility assay, primary human AAA patient-derived VSMCs treated with lenvatinib displayed enhanced contractility in comparison with vehicle-treated cells (Figure 4A and Supplemental Figure 9C) and significant upregulation of contractile cell markers (i.e., MYOCD and ACTA2) 24 hours after treatment initiation (Figure 4B and Supplemental Figure 9D).

In accordance with previously shown immunohistochemistry and gene expression data, Western blot analysis also detected MYH11 markedly increased upon lenvatinib treatment (Figure 4C and Supplemental Figure 9, A and B). When exploring possible downstream signaling pathways affected by lenvatinib, we discovered a decrease in the phosphorylated ERK1-2/total ERK1-2 ratio in all cell experiments after 6 and 48 hours of lenvatinib exposure (Figure 4D and Supplemental Figure 10).

### Lenvatinib-coated balloon angioplasty blocks aneurysm progression in a preclinical Yucatan LDLR^–/–^ minipig model.

**Figure 5:**
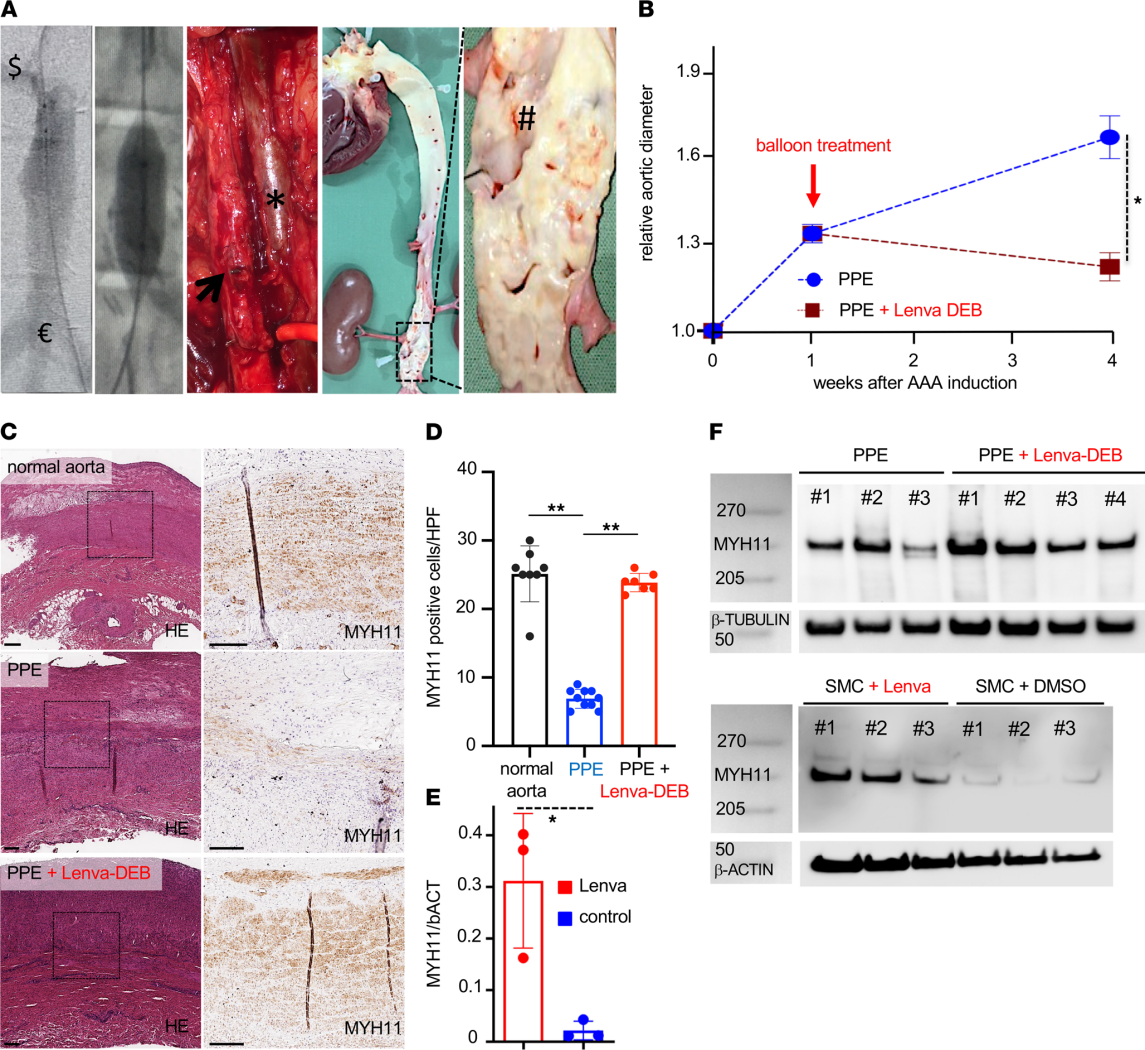
Endovascular lenvatinib treatment LDLR–/– minipigs with AAA disease. (**A**) Transfemoral angiography of the infrarenal aorta ($ = right renal artery to € = aortic trifurcation) on day 7 after AAA induction shows the dilated aortic segment. Radiopaque markers guided the DEB (12 *×* 20 mm) to the lesion. Surgical clips from the aneurysm induction procedure sealed the lumbar vessels. The photograph shows the dilated part of the aorta (arrow) at the time of sacrifice (no circulating blood) next to the cava vein (asterisk). En face preparation of the complete aorta demonstrates severe atherosclerosis and fatty streaks at the level of the aortic valve, the coronary and supra-aortic branch ostia, and the infrarenal level and the renal artery (#). (**B**) Singular treatment with a lenvatinib-coated balloon (red: Lenva-DEB; *n* = 4) on day 7 after PPE-AAA induction shows a significant aortic diameter reduction compared with nontreated (blue: PPE only; *n* = 7) animals (*P < 0.05; 2-way repeated measures ANOVA). (**C**) H&E indicates a parallel fibrous structure in the nonintervened (normal) *LDLR^–/–^* pig aorta with marked noncalcified plaque formation. Similar to the murine PPE-AAA model, a substantial MYH11 restoration was detected upon local lenvatinib treatment (Lenva-DEB) compared with PPE-AAAs. (**D**) Quantification of MYH11-positive cells confirmed loss of MYH11 in PPE-AAA minipigs compared with untreated (normal) *LDLR^–/–^* aorta and restoration upon Lenva-DEB treatment. (**E**) WB analysis confirmed higher MYH11 protein content in Lenva-DEB–treated minipigs compared with DEB-controls (PPE) with progressing AAA. (**F**) MYH11 WB analysis on primary pig aortic cells treated for 48 hours with lenvatinib (normalized to β-actin). (*P < 0.05, **P < 0.01; original magnification 5×/20×; scale bar: 200 μm; vessel lumen oriented upward; complete WB and quantifications are shown in Supplemental Figure 13.)

To further test the potential of a local endovascular delivery of lenvatinib to halt aneurysm growth, we established a large animal model with inducible AAA in hypercholesterolemic LDL receptor–knockout (*LDLR^–/–^*) Yucatan minipigs (Supplemental Figure 11, A and B). Unlike *Ldlr^–/–^* mice, which almost solely develop aortic lesions in their arch, *LDLR*-deficient minipigs presented with severe atherosclerotic disease throughout the entire vascular system, including the infrarenal aorta (Figure 5A). Thus, this preclinical model very closely resembled the aged vascular phenotype of human AAA disease (Supplemental Figure 11D, Supplemental Figure 12, and Supplemental Figure 14) (14). In addition to more closely mimicking the advanced human AAA phenotype, *LDLR^–/–^* minipigs developed more enlarged AAAs compared with their WT normo-cholesterolemic companions (82 ± 24% vs. 50 ± 13% diameter increase; *P* < 0.05) 28 days after PPE induction. At baseline, the average diameter in *LDLR^–/–^* (0.74 ± 0.56 cm) and *LDLR^+/+^* (0.75 ± 0.52 cm) was almost identical, highlighting the proaneurysmal effect of the hypercholesterolemic minipigs.

The PPE instillation itself resulted in a significant increase of the aortic diameter (week 1: 21 ± 15%, *P* = 0.0004; week 4: 56 ± 21%, *P* = 0.000002; *n* = 7; Figure 5B and Supplemental Table 1) compared with sham operation (PBS perfusion; at week 4: 10 ± 9%; Supplemental Figure 11E). Compared with nondilated *LDLR^–/–^* minipig aortas, PPE-induced aneurysms presented with significantly reduced MYH11 levels and CD31^+^ neovessels in the aortic media of the dilated vessels (Figure 5, C and D, and Supplemental Figure 12).

Drug-eluting balloon–delivered (DEB-delivered) lenvatinib treatment, 7 days after aneurysm induction, was capable of significantly reducing AAA diameters after 28 days in comparison with a control (vehicle-only) DEB intervention in PPE-AAA animals (31% increase with lenvatinib-DEB vs. 68% increase with control-DEB; *P* = 0.006; Figure 5, A and B). Only the DEB control intervention using vehicle (DMSO) had no effect on aortic diameter progression compared with regular AAA development in PPE-induced AAAs in this porcine model (Supplemental Figure 11E).

**Figure 6:**
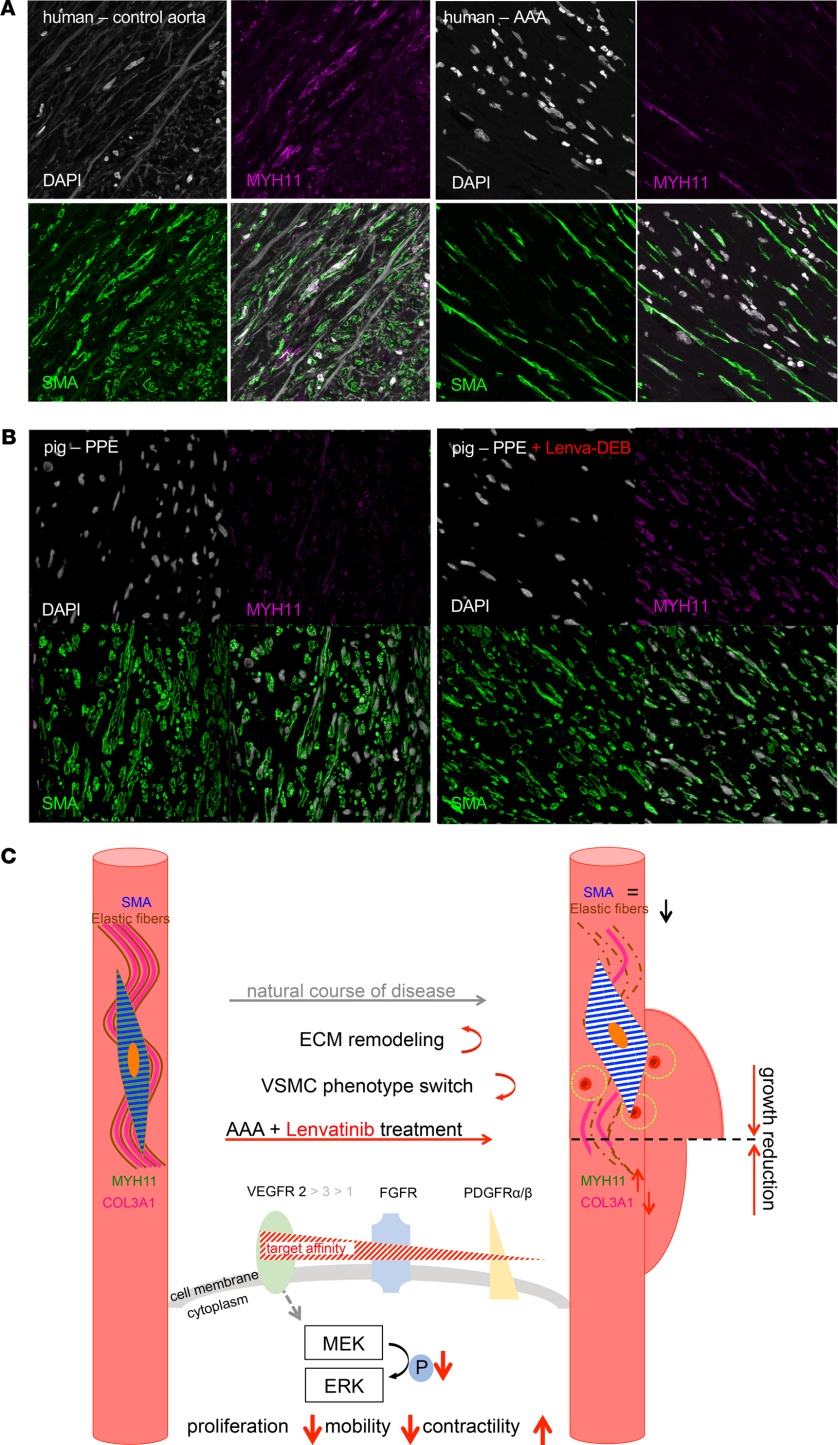
Contractile element MYH11 loss in human AAA and restoration upon DEB-delivered lenvatinib treatment in pigs; proposed mechanism of action. (**A**) Immunofluorescence staining of αSMA, MYH11, and nuclear DAPI in human control aorta and AAA and (**B**) in Yucatan *LDLR^–/–^* PPE-AAA minipig model untreated and treated with lenvatinib. Original magnification, 63×. (**C**) Scheme of the proposed mechanism of action for lenvatinib in the context of aneurysm development. Lenvatinib inhibits the tyrosine kinase intracellular signal by reducing ERK1-2 phosphorylation in aortic SMCs. This event is associated with decreased proliferation. In parallel, lenvatinib seems to improve SMC contractility, possibly by MHY11 restoration, resulting in a potential slowdown of aneurysmal growth.

Immunohistochemical analysis revealed high abundance of MYH11 colocalizing with αSMA in the aortic media (Figure 5, C and D; Figure 6B; and Supplemental Figure 12). Consistently, Western blot confirmed this result, with DEB-lenvatinib–treated pigs showing higher MYH11 protein levels compared with DEB-controls (Figure 5E). Accordingly, lenvatinib treatment significantly increased MYH11 protein levels in VSMCs isolated from healthy porcine aorta in vitro (Figure 5F and Supplemental Figure 13A). Body weight and blood parameters (white and red blood cell counts; measures of liver and kidney function) were unchanged between the treatment and control groups (Supplemental Table 3).

### Human AAA tissue is deficient in VSMC contractile elements.

Phenotypic switching in VSMCs in AAA disease has been demonstrated numerous times before (6, 12, 15). We analyzed human AAA samples from an advanced disease state (6–7 cm diameter) from patients undergoing open aortic repair. Here, the morphology of AAA compared with nondilated infrarenal aortic controls was completely altered in terms of elastic fibers’ degradation, disruption of the media and adventitia, angiogenesis in the aortic media, as well as inflammatory infiltrates (Supplemental Figure 14B). Gene expression analysis of characteristic SMC markers indicated a definite loss of contractile features in AAA compared with nondilated control aortas (Supplemental Table 2). Double-immunofluorescence staining confirmed MYH11 being colocalized with αSMA in nondilated aortas but almost entirely absent in advanced AAA (Figure 6A, Supplemental Figure 14A, and Supplemental Figure 15).

## Discussion

In this study, we successfully “repurpose” the anticancer drug lenvatinib to halt experimental AA growth in 2 species and patient-derived primary human cells via reconstitution of VSMC contractility (Figure 6C).

Our study provides evidence for what is likely the first noncancer application of the TKI lenvatinib (16, 17). Furthermore, for the first time to our knowledge, we successfully treat animals with an already existing AAA, 7 days after aneurysm induction, and thus very closely mimic the translational need for treating an established aneurysm in patients (Figure 1A; Figure 2, B and E; Figure 5A; and Supplemental Figure 4, A and B). Previous drug interaction studies started treatment in mice before — or at the time of — aneurysm induction (day 0), thus before disease onset, which might mainly counteract inflammatory processes seen in the early phases of all animal AAA models (7, 18, 19). Imatinib and erlotinib are 2 TKIs with different target receptors (c-abl and epidermal growth factor receptor, respectively) that have shown the capacity to attenuate experimental aneurysm growth in the murine AngII-induced aortic dissection model (20, 21).

In cancer therapy, lenvatinib is employed mainly for its antiangiogenic effect (16). Neo-angiogenesis in the aortic media is observed in human AAA and in the murine PPE model but remains absent in nondilated aortas (Figure 1, C–E; Figure 3B; and Figure 5D) (22, 23). We observed limited CD31/CD34 positivity as a hallmark of reduced angiogenesis in all lenvatinib-treated animals, as well as reduced expression of VEGFR2 in human AAA patient-derived primary cells (Figure 1E, Figure 2D, Figure 3B, and Figure 4B). Similarly, we have previously demonstrated aneurysm abrogation in the murine PPE and AngII model by blocking the long noncoding RNA H19, which represses HIF1α expression and subsequent angiogenesis (5). Interference with the HIF1α/VEGF/VEGFR axis has been shown multiple times to hinder aneurysm growth in mouse models (4, 23, 24). One of the major reasons to select lenvatinib for our study was that lenvatinib — of all TKIs — has the highest affinity for VEGFR2, which can be found abundantly in both human and experimental AAA (22, 25, 26).

Despite angiogenesis, the fate of VSMCs in the aortic media may crucially determine the aneurysm expansion rate. HIF1α is likely a key link between angiogenesis and VSMC phenotype polarization (5, 27). Along with fragmentation of the elastic fibers and reorganization of the ECM architecture, the number of VSMCs declines substantially during AAA progression, while turnover increases with cells losing their contractile phenotype (3, 28). These effects are strongly opposed by treatment with low-dose lenvatinib, both on a cellular level and in experimental AAA in mice and minipigs. While apoptosis seemed largely unaffected (an increase was only detectable in donor VSMCs) (Figure 3A, Supplemental Figure 5A, and Supplemental Figure 7A), mobility and proliferation were markedly decreased, and many genes involved in maintaining the contractile phenotype (e.g., *TAGLN*, *CNN1*, *MYOCD*, *ITGA8*, *CALD1*) became strongly upregulated upon TKI treatment (Figure 3, Figures 5, and Figure 6). However, *COL3A1*, which encodes the pro-α1 chains of type III collagen as the main fibrillar component of the nondiseased aorta, typically expressed by synthetic SMCs, appeared repressed upon lenvatinib stimulation in all cell experiments, highlighting its effect in regulating phenotypic SMC transitions toward the contractile state. MYH11, a prototypical contractile marker, was substantially restored by lenvatinib (Figure 1B; Figure 4C; Figure 5, E and F; Figure 6B; Supplemental Figure 9, A and B; and Supplemental Figures 12 and 13). Its regulatory role and contribution to an intact aortic wall, other than being a marker of the contractile apparatus, are largely unknown. However, it has been found repressed in aneurysmatic conditions in several studies (29–31). Targeting VSMC dynamics and fate using RNA-based therapies or chemotherapeutics has largely been proved effective in different animal models of AAA (32, 33). Moreover, ERK phosphorylation is a crucial mediator of AAA expansion and VSMC differentiation. Various substances are capable of blocking ERK-1 phosphorylation and limiting aortic dilation (34–36).

Interestingly, the magnitude of differential gene expression and the responsiveness to lenvatinib in cellular assays was enhanced in diseased (AAA patient-derived) cells compared with healthy donor cells. This suggests that the diseased state of VSMCs is more susceptible to an external stimulus or treatment. Importantly, AAA patient-derived cells and healthy donor cells stem from the infrarenal aorta, not permitting the embryologic origin to play any role in the outcome of our experiments. Interestingly, a higher extent of intracellular damage can be observed in AAA patient-derived cells (12). This supports our recent attempts to initiate treatment not at the time of AAA induction in murine models, but rather when a dilation and progression of disease have already been established (7).

Apart from systemic forms of application, we demonstrate the effectiveness of a local endovascular application of lenvatinib in mice and a novel atherosclerotic minipig model. The PPE procedure in pigs has successfully been performed before (37). However, by using *LDLR^–/–^* animals with advanced atherosclerosis, we are convinced we achieved a closer mimicry of advanced human AAA pathology (14). While only male mice have been utilized to exclude sex-specific effects, a mixed group of female and castrated male pigs were used. Nevertheless, a very uniform aneurysm formation could be observed (38).

From a translational perspective, the local treatment with a lenvatinib-coated balloon or stent (-graft) could be an amendment to difficult AAA morphologies with problematic proximal or distal sealing zones, as well as for otherwise inoperable patients (39, 40). In ophthalmology, intravitreal bevacizumab, a monoclonal VEGF blocker, is already in common use for retinal artery aneurysm treatment (41). We did not observe any side effects based on overall survival or organ function in our lenvatinib treatment group. However, side effects are certainly possible and should be further evaluated in a more chronic, extended experimental setup with greater sample size, especially when considering the current debate on long-term effects of paclitaxel-coated balloons (42, 43).

## Methods

Methods unique to this study are summarized below. Additional materials and methods are presented in the Supplemental Data.

### PPE and AngII induction of murine aortic aneurysms.

See *Study approval* for an ethics declaration. The PPE (MilliporeSigma) model was described various times before (13, 44). Briefly, 10-week-old male C57BL/6J WT mice (Taconic Biosciences) on regular chow diet (R36; Lantmännen) were kept under standard conditions (temperature 22°C, humidity 56%). Similarly, AngII-minipump implantation for AAA induction has been described various times before (19). Briefly, 10-week-old ApoE^–/–^ male mice (Charles River Laboratories) received an AngII-loaded (Bachem) osmotic minipump (model 2004, DURECT Corporation) implanted subcutaneously. Anesthesia, PPE-AAA induction, AngII-AAA induction, murine B-mode ultrasound images, and protocols for euthanasia and tissue processing are discussed in the Supplemental Data.

### Oral lenvatinib treatment in mice.

Anesthesia was induced using isoflurane. Each animal was fed an individual body weight–dependent dose (5 mg/kg) of 2.5 mg/mL lenvatinib (E7080; catalog S1164, Selleckchem) solution dissolved in DMSO (MilliporeSigma) as used and reported before by others (11). Oral gavage was performed by placing a pipet tip in glucose and then down the animal’s throat. This procedure was repeated daily from day 7 (for PPE mice) and day 4 (for AngII mice) until day 24 after AAA induction. The procedure was performed on 7 PPE and 6 AngII animals (no deaths, average procedure time 4 minutes per animal including anesthesia). Additionally, 5 AngII mice received oral gavage of PBS as a sham group.

### Local lenvatinib treatment in mice.

Anesthesia was induced with isoflurane, and analgesia was provided with buprenorphine (4 mg/kg), before and after surgery, 7 days after the initial AAA induction with PPE. The retroperitoneum was reexposed, and the established AAA was carefully dissected from the adherent soft tissue (illustrated in Figure 2A). Proximal to the initial aortic incision, a second intervention was performed using a 30 G needle. Aneurysms were then perfused with a 2.5 mg/mL lenvatinib solution in DMSO for 10 minutes via an inserted microcatheter. Thereafter, the aorta was flushed with warm saline, and a single knot suture, 10-0, was placed to seal the catheter entry site. After layered closure of the abdomen, the animal was allowed to recover under an infrared lamp. The procedure was performed on 6 animals; 1 animal died due to surgical blood loss during dissection (procedure time approximately 55 minutes including anesthesia).

### Mouse transcriptomic array analysis.

RNA profiling was performed using the Mouse Transcriptome Assay 1.0 (Affymetrix). Differential expression analysis was achieved by utilizing the Transcriptome Analysis Console v4.0.1.36 (Affymetrix) in accordance with the manufacturer’s default settings and SST-RMA summarization method. Three separate groups (nondilated control aorta, PPE-AAA, PPE-AAA+Lenva treatment; same region of infrarenal aorta in all animals) of mouse aortic tissues were analyzed for differential expression. The results were exported and further processed using RStudio v1.1.463/Rv3.5.3. Raw exported data and R script files used for the analysis can be found in the Supplemental Data. When applicable, for downstream analyses, standard filtering was performed (*P* < 0.05, absolute fold change ≥ 2), and certain groups of genes were removed (unannotated genes, predicted genes, genes coding for immunoglobulin chains). Data from a separate in-house MTA 1.0 data set of early PPE model mice (PPE-AAA [*n* = 6], saline-Ctrl [*n* = 5]; same region of infrarenal aorta harvested at day 7) were used to present baseline transcriptomic characteristics of the model at the time point of Lenva treatment initiation, as shown in Figure 2E and Supplemental Figure 4A. KEGG pathway gene overrepresentation analysis was performed using clusterProfiler v3.16.1 using directional sets of differentially regulated genes (Benjamini-Hochberg adjusted *P* < 0.05, absolute fold change either < –2 or > 2). Plotting was performed using the ggplot2 v3.3.3 library (45).

### AAA induction in Yucatan minipigs.

See *Study approval* for an ethics declaration. Twelve 13-month-old female and castrated male *LDLR^–/–^* Yucatan minipigs (77.8 ± 5.1 kg body weight) from Exemplar Genetics (Coralville, Iowa, USA) were used. Minipigs were housed in groups of 2–4 under conventional hygienic conditions and an acclimatization period of at least 9 days prior to surgery (temperature 19 ± 2°C; humidity 50–60%; 12-hour light/12-hour dark cycle; environmental enrichment provided). Animals were fed with a pelleted high-fat diet (Altromin) twice a day and received water ad libitum. Prior to anesthesia, animals were starved for 12 hours with free access to water.

All experiments commonly consisted of 3 distinct procedures: baseline/day 0 (ultrasound and AAA induction with PPE), day 8 (ultrasound and endovascular coated balloon treatment via femoral access), and day 28 (ultrasound, euthanasia, and tissue collection). Detailed protocols for anesthesia and ultrasound measurements can be found in the Supplemental Data. For the PPE induction, a retroperitoneal exposure of the aorta was achieved via a left lateral flank access. Preparation included anteflexion of the left kidney and complete dissection of the aorta from the left renal artery to the aortic trifurcation. Either 2 or 3 pairs of lumbar arteries were clipped and divided. A total of 3000 IU heparin was administered before clamping of the aorta over a distance of 3–4 cm. A 9–11 *×* 20 mm PTA balloon (Medtronic Admiral Xtreme) based on previous ultrasound measurements (diameter + 2 mm) was inserted via puncture incision and inflated to 10 atm for 1 minute to predilatate the aorta, allowing better perfusion with the elastase. Afterward, a blunt needle (5 G) was inserted, and perfusion with 10 IU/mL PPE (MilliporeSigma) was started after a tourniquet secured the needle at the puncture site. The aorta was perfused to the maximum diameter before effusion of elastase through the vessel for 10 minutes using a pressure syringe was permitted. Afterward, the aorta was flushed with heparinized saline (1000 IU/L) and the incision closed with a 5-0 suture before reopening the clamp. Layer-by-layer closure including 2× muscle, fat, and skin was performed, and the wound was coated with a spray-on dressing (Aqueos).

### Endovascular DEB treatment in Yucatan minipigs.

The animals were put in supine position. A 4 cm incision into the right groin was made, and the common femoral artery was surgically exposed. A total of 3000 IU of heparin was administered. The vessel was then ligated distally before a 5F introducer sheath (TerumoAortic) was placed in retrograde direction. Using a guide wire (TerumoAortic) and a mobile C-arm, a 4F pig tail catheter (Aimecs) was advanced into the aorta. An angiogram using 6 mL of contrast agent (Imeron) to identify aortic landmarks and the aneurysmatic lesion was performed. A 10–12 *×* 20 mm PTA balloon (Medtronic Admiral Xtreme) was then spray coated with a 50 mM lenvatinib (E7080) solution. Using a special introducer sheath to protect the coated area, the balloon was advanced to the identified aneurysm site and inflated to 10 atm for 3 minutes. Afterward a control angiography excluded aortic occlusion or dissection. The common femoral artery was ligated proximally to the puncture site. Layer-by-layer closure was performed and the wound coated with spray-on dressing. Postoperative analgesia, blood sampling, and euthanasia protocols can be found in the Supplemental Data.

### Double immunofluorescence staining.

Sections of 2.5 μm of paraffin-embedded samples were mounted on precoated SuperFrost Plus slides (Thermo Fisher Scientific). Antigen retrieval and blocking of peroxidase activity were performed as described for immunohistochemistry. Additional blocking with 5% horse serum was performed for 1 hour. Primary and secondary antibodies (Supplemental Table 4) were diluted in 5% horse serum. Both primary antibodies were incubated after another overnight each at 4°C, followed by both secondary antibodies for 1 hour each at the respective day. TrueBlack Lipofuscin Autofluorescence Quencher (Biotium) was applied to reduce background fluorescence. Sections were counterstained with DAPI (Thermo Fisher Scientific), and images were taken under a confocal microscope (TCS SP5 II, Leica). For negative controls, only the secondary antibody was applied for 1 hour at room temperature; 5% horse serum without any primary antibody was applied overnight.

### Primary cell culture of human aortic aneurysm VSMCs.

Written and informed consent was obtained from all patients, and protocols were approved by the local ethics committee (46). Human AAA material was harvested during surgical repair and stored in complete DMEM/F12 Medium (MilliporeSigma, containing 5% FBS and 1% PBS). The tissue was placed in a sterile Petri dish and washed with PBS. Adventitia, neo-intima, and calcifications were removed, and the remaining media were cut into small pieces using a sterile scalpel. The pieces of tissue were placed in digestion medium (1.4 mg/mL Collagenase A, Roche, in complete DMEM/F12 Medium) in a humidified incubator at 37°C and 5% CO_2_ for 4–6 hours. Cells were strained using a 100 μm cell strainer to remove debris. After 2 washings (centrifuge 400*g*, 5 minutes; discard supernatant; resuspend in 15 mL complete DMEM/F12 Medium) cells were resuspended in 7 mL complete DMEM/F12 Medium and placed in a small cell culture flask in a humidified incubator at 37°C and 5% CO_2_. Medium was changed every other day. After 1 week the medium was replaced by SMC Growth Medium (PeloBiotech). Upon confluence, cells were stored in liquid nitrogen or processed immediately. In this study, primary cells from 3 different patients with AAAs (diameter 6.7 ± 0.4 cm) were obtained. Cells were used between passages 3 and 7. Primary abdominal aortic SMCs from healthy donors (control SMCs) were purchased from Cell Applications (354-05a) and used between passages 5 and 7.

### Dynamic live-cell imaging assays.

All dynamic live-cell imaging experiments were performed following the instructions provided by Essen Bioscience using the IncuCyte ZOOM System. For the dose finding experiment, cells were plated in 24-well plates (Corning). The next day medium was changed to OptiMEM + 10% FBS + 1% PBS-Tween (Thermo Fisher Scientific). Lenvatinib (E7080) was dissolved in 2.3 mL DMSO (according to commercial protocol) to produce the 10 mM stock solution. This stock was diluted into working concentrations: 10 μM, 1 μM, and 0.1 μM. A total of 1 μL DMSO was added to control wells. Confluence was assessed over 72 hours using the IncuCyte ZOOM System. For proliferation and apoptosis, cells were placed with 30% confluence in a 96-well plate and treated with 0.1 μM lenvatinib in OptiMEM (Thermo Fisher Scientific; lenvatinib treatment) or 0.1% DMSO in OptiMEM (control treatment) for 72 hours. The IncuCyte Caspase-3/7 Apoptosis Reagent (Essen Bioscience) was added at a final concentration of 5 μM. The plate was monitored in the IncuCyte ZOOM System (Essen Bioscience) with phase/fluorescence and a 2-hour imaging pattern. Images were autocollected and analyzed using the IncuCyte ZOOM software (Essen Bioscience). For migration, cells were placed at 100% confluence in 96-well ImageLock plates (Essen Bioscience), and homogeneous 700–800 μm wide wounds were created using the WoundMaker (Essen Bioscience). Cells were treated with 0.1 μM lenvatinib in OptiMEM (Thermo Fisher Scientific, lenvatinib treatment) or 0.1% DMSO in OptiMEM (control treatment) for 72 hours, and the plate was monitored in the IncuCyte ZOOM System (Essen Bioscience) with a 2-hour imaging pattern. Images were autocollected and analyzed using the IncuCyte ZOOM software (Essen Bioscience).

### Cell contraction assay.

Cell Contraction Assay (CBA-202, Cell Biolabs Inc.) was performed according to the manufacturer’s protocol. Briefly, 397.5 μL Collagen Solution, 102.5 μL of 5× PBS, and 14.2 μL neutralization solution were mixed on ice. Human primary aortic cells were detached from culture flasks and suspended in SMC Growth Medium (see above). A total of 125 μL of cell suspension at a final concentration of 1.2 million cells/mL was mixed with the collagen matrix, and 500 μL aliquots were placed in a 24-well plate. The cell-containing matrix polymerized at 37°C and 5% CO_2_ for 1 hour. Then 1 mL SMC Medium was added to the matrices. After 48 hours at 37°C and 5% CO_2_, 0.5 mL of SMC Medium was added to each well to reach the following final concentrations: lenvatinib 10 μM, DMSO 0.1%, bradykinin 10 nM (B3259-1MG, MilliporeSigma), and BDM 10 mM (100× stock included in the kit). Matrices were released using a sterile spatula. Images were taken every 15–30 minutes for 6 hours and then after 24 hours. The diameter of each matrix was measured using a ruler and normalized to the vessel. After 24 hours, 200 μL QIAzol was added to each matrix, and RNA was isolated using miRNeasy Micro Kit (217084, QIAGEN).

### Statistics.

Ultrasound data from animals are shown relative to the individual baseline diameter of the aorta (i.e., Figure 1A) and as absolute aortic diameter (Supplemental Figure 1), both as mean ± 1 SEM. Statistical testing of ultrasound diameter measurements of all experimental animal model aortas, except for murine PPE model, was performed using multiple 1-tailed *t* test (1 per row) with post hoc Holm-Šídák correction for multiple comparisons (Supplemental Table 1). Due to increased complexity, the ultrasound measurements of murine PPE model aortas were analyzed using a repeated measures 2-way ANOVA approach (Supplemental Table 1). Post hoc pairwise comparisons were thereafter performed using Fisher’s least significant difference test with Holm-Šídák correction for multiple comparisons. Quantitative PCR gene expression data are shown, using the ΔΔCt-method normalized to housekeeping genes and individual control vessel (fold change = 2-ΔΔCt) as mean and standard deviation. A Welch (1-tailed) *t* test for unpaired samples of small numbers (parametric) was used with a level of less than 0.05 considered significant for gene expression data. For comparison of IncuCyte live-cell imaging experiments, data are presented as mean ± 1 SEM. Significance thresholds are defined as follows: *P < 0.05, **P < 0.01, ***P < 0.001, ****P < 0.0001. GraphPad Prism was used to prepare graphs. Microsoft PowerPoint was used to establish composite figures.

### Study approval.

All procedures were carried out in accordance with the protocols approved by the animal care committee of the Karolinska Institutet (PPE: N119/15 and Amendment N229/15) or the Austrian Ministry of Science (AngII: BMWFW-66.009/0355-WF/V/3b/2016 and amendment 2020-0.547.543) and in compliance with the guidelines on animal welfare of the Committee for Animal Experiments. The pig study was performed in compliance with the EU Directive 2010/62/EU for animal experiments and the German Animal Welfare Act (2018). All pig procedures and animal handling were approved by the Animal Research Ethical Committee of the Government of Upper Bavaria (Munich, Germany; protocol ROB-55.2-2532.Vet_02-18-53). Human sample collection from subjects who gave written and informed conset was approved by the ethics committee of Technical University of Munich.

## Author contributions

AB, VP, and LM conceived and designed the study; AB, JP, VP, GW, CO, CK, HHE, and LM analyzed and interpreted data; AB, JP, VP, SB GW, EC, JP, SM, HW, MT, AW, CO, JF, J Reiser, JW, J Roy, CB, and CK collected data; AB and LM wrote the article; AB, JP, VP, J Roy, HHE, CO, CB, and LM critically revised the article; all authors gave final approval of the article; AB, VP, GW, and HW performed statistical analysis; AB, HHE, and LM obtained funding; and AB had overall responsibility.

## Supplementary Material

Supplemental data

## Version 1 06/29/2021

In-Press Preview

## Version 2 08/09/2021

Electronic publication

## References

[1] Nordon IM (2011). Pathophysiology and epidemiology of abdominal aortic aneurysms. Nat Rev Cardiol.

[2] Wanhainen A (2019). Editor’s choice — European society for vascular surgery (ESVS) 2019 Clinical practice guidelines on the management of abdominal aorto-iliac artery aneurysms. Eur J Vasc Endovasc Surg.

[3] Michel JB (2011). Novel aspects of the pathogenesis of aneurysms of the abdominal aorta in humans. Cardiovasc Res.

[4] Xu B (2019). Inhibition of VEGF (vascular endothelial growth factor)-A or its receptor activity suppresses experimental aneurysm progression in the aortic elastase infusion model. Arterioscler Thromb Vasc Biol.

[5] Li DY (2018). H19 induces abdominal aortic aneurysm development and progression. Circulation.

[6] Peng H (2018). VPO1 modulates vascular smooth muscle cell phenotypic switch by activating extracellular signal-regulated Kinase 1/2 (ERK 1/2) in abdominal aortic aneurysms. J Am Heart Assoc.

[7] Busch A (2018). Four surgical modifications to the classic elastase perfusion aneurysm model enable haemodynamic alterations and extended elastase perfusion. Eur J Vasc Endovasc Surg.

[8] Lindeman JH (2015). The pathophysiologic basis of abdominal aortic aneurysm progression: a critical appraisal. Expert Rev Cardiovasc Ther.

[9] Hodos RA (2016). In silico methods for drug repurposing and pharmacology. Wiley Interdiscip Rev Syst Biol Med.

[10] Schlumberger M (2015). Lenvatinib versus placebo in radioiodine-refractory thyroid cancer. N Engl J Med.

[11] Wiegering A (2014). E7080 (lenvatinib), a multi-targeted tyrosine kinase inhibitor, demonstrates antitumor activities against colorectal cancer xenografts. Neoplasia.

[12] Riches K (2018). Progressive development of aberrant smooth muscle cell phenotype in abdominal aortic aneurysm disease. J Vasc Res.

[13] Busch A (2016). Extra- and intraluminal elastase induce morphologically distinct abdominal aortic aneurysms in mice and thus represent specific subtypes of human disease. J Vasc Res.

[14] Davis BT (2014). Targeted disruption of LDLR causes hypercholesterolemia and atherosclerosis in Yucatan miniature pigs. PLoS One.

[15] Sachdeva J (2017). Smooth muscle cell-specific Notch1 haploinsufficiency restricts the progression of abdominal aortic aneurysm by modulating CTGF expression. PLoS One.

[16] Cabanillas ME, Habra MA (2016). Lenvatinib: role in thyroid cancer and other solid tumors. Cancer Treat Rev.

[17] Kudo M (2018). Lenvatinib versus sorafenib in first-line treatment of patients with unresectable hepatocellular carcinoma: a randomised phase 3 non-inferiority trial. Lancet.

[18] Di Gennaro A (2018). Cysteinyl leukotriene receptor 1 antagonism prevents experimental abdominal aortic aneurysm. Proc Natl Acad Sci U S A.

[19] Maegdefessel L (2014). miR-24 limits aortic vascular inflammation and murine abdominal aneurysm development. Nat Commun.

[20] Obama T (2015). Epidermal growth factor receptor inhibitor protects against abdominal aortic aneurysm in a mouse model. Clin Sci (Lond).

[21] Vorkapic E (2016). Imatinib treatment attenuates growth and inflammation of angiotensin II induced abdominal aortic aneurysm. Atherosclerosis.

[22] Busch A (2017). Heterogeneous histomorphology, yet homogeneous vascular smooth muscle cell dedifferentiation, characterize human aneurysm disease. J Vasc Surg.

[23] Wang W (2018). Hypoxia-inducible factor 1 in clinical and experimental aortic aneurysm disease. J Vasc Surg.

[24] Choke E (2010). Vascular endothelial growth factor enhances angiotensin II-induced aneurysm formation in apolipoprotein E-deficient mice. J Vasc Surg.

[25] Tedesco MM (2009). Analysis of in situ and ex vivo vascular endothelial growth factor receptor expression during experimental aortic aneurysm progression. Arterioscler Thromb Vasc Biol.

[26] Miwa K (2005). Inhibition of ets, an essential transcription factor for angiogenesis, to prevent the development of abdominal aortic aneurysm in a rat model. Gene Ther.

[27] Liu K (2017). Hypoxia-inducible factor 1a induces phenotype switch of human aortic vascular smooth muscle cell through PI3K/AKT/AEG-1 signaling. Oncotarget.

[28] Petsophonsakul P (2019). Role of vascular smooth muscle cell phenotypic switching and calcification in aortic aneurysm formation. Arterioscler Thromb Vasc Biol.

[29] Yokoyama U (2018). Proteomic analysis of aortic smooth muscle cell secretions reveals an association of myosin heavy chain 11 with abdominal aortic aneurysm. Am J Physiol Heart Circ Physiol.

[30] Kurtelius A (2019). Association of intracranial aneurysms with aortic aneurysms in 125 patients with fusiform and 4253 patients with saccular intracranial aneurysms and their family members and population controls. J Am Heart Assoc.

[31] Yeung KK (2017). Transdifferentiation of human dermal fibroblasts to smooth muscle-like cells to study the effect of MYH11 and ACTA2 mutations in aortic aneurysms. Hum Mutat.

[32] Li G (2017). Inhibition of the mTOR pathway in abdominal aortic aneurysm: implications of smooth muscle cell contractile phenotype, inflammation, and aneurysm expansion. Am J Physiol Heart Circ Physiol.

[33] Leeper NJ, Maegdefessel L (2018). Non-coding RNAs: key regulators of smooth muscle cell fate in vascular disease. Cardiovasc Res.

[34] Ghosh A (2012). The role of extracellular signal-related kinase during abdominal aortic aneurysm formation. J Am Coll Surg.

[35] Chen PY (2009). FRS2 via fibroblast growth factor receptor 1 is required for platelet-derived growth factor receptor beta-mediated regulation of vascular smooth muscle marker gene expression. J Biol Chem.

[36] Moran CS (2017). Resveratrol inhibits growth of experimental abdominal aortic aneurysm associated with upregulation of angiotensin-converting enzyme 2. Arterioscler Thromb Vasc Biol.

[37] Hynecek RL (2007). The creation of an infrarenal aneurysm within the native abdominal aorta of swine. Surgery.

[38] Cho BS (2009). Differential regulation of aortic growth in male and female rodents is associated with AAA development. J Surg Res.

[39] Oliveira NFG (2019). Patients with large neck diameter have a higher risk of type IA endoleaks and aneurysm rupture after standard endovascular aneurysm repair. J Vasc Surg.

[40] Bryce Y (2018). Outcomes over time in patients with hostile neck anatomy undergoing endovascular repair of abdominal aortic aneurysm. J Vasc Interv Radiol.

[41] Pichi F (2013). Intravitreal bevacizumab for macular complications from retinal arterial macroaneurysms. Am J Ophthalmol.

[42] Xu L (2017). Abdominal aortic dissection during sorafenib therapy for hepatocellular carcinoma. Clin Res Hepatol Gastroenterol.

[43] Behrendt CA (2020). Editor’s choice - long term survival after femoropopliteal artery revascularisation with paclitaxel coated devices: a propensity score matched cohort analysis. Eur J Vasc Endovasc Surg.

[44] Azuma J (2009). Creation of murine experimental abdominal aortic aneurysms with elastase. J Vis Exp.

[45] Wickham H. *ggplot2: Elegant Graphics for Data Analysis*. Springer; 2016.

[46] Pelisek J (2019). Biobanking: objectives, requirements, and future challenges-experiences from the Munich vascular biobank. J Clin Med.

