# Supplementary material for: Lenvatinib halts aortic aneurysm growth by restoring smooth muscle cell contractility
Source: JCI Insight. 2021 Aug 9;6(15):e140364. doi: 10.1172/jci.insight.140364 (PMC8410098; doi:10.1172/jci.insight.140364)
Supplement: Supplemental data [file jciinsight-6-140364-s038.pdf]

Suppl. Fig. 1

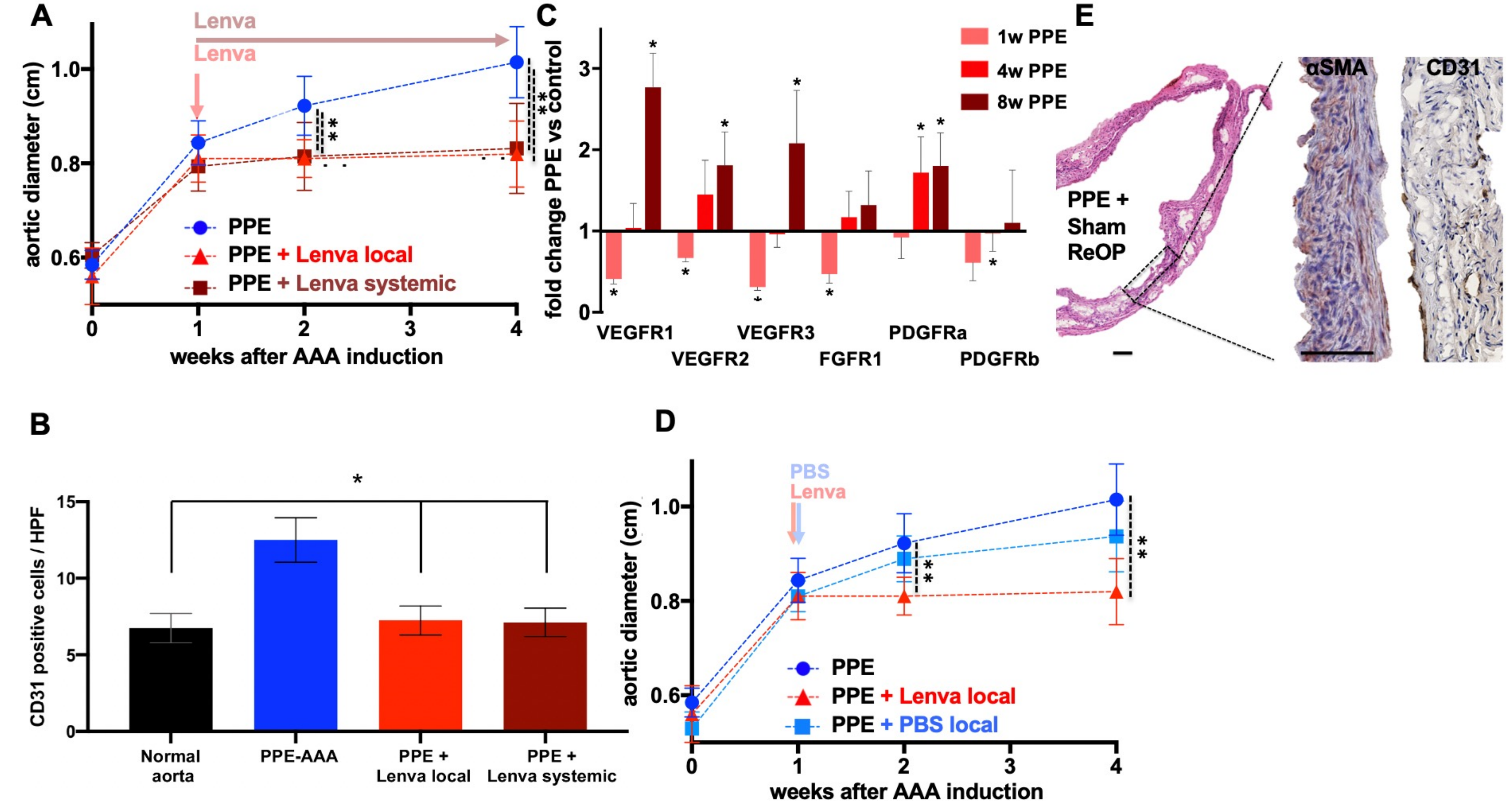

Suppl. Figure 1: Additional murine PPE-AAA data: (A) Absolute aortic diameters in cm for the murine PPE and lenvatinib experiments (n=11 for PPE; n=7 for Lenva systemic; n=5 for Lenva local; **=p<0.01; mean + SEM; PPE AAA induction at day 0). **(B)** Quantification of CD31 positive cells using high power fields (HPF) analysis of representative images presented in Figures 1C-E (*=p<0.05; mean + SEM). **(C)** Lenvatinib target receptors show a time-dependent up-regulation after initial suppression in PPE-AAA mice compared to non-treated control animals over a 8-week period (n=5 for controls, PPE 1 week, PPE 4 weeks and n=4 for PPE 8 weeks; *=p<0.05; mean + SEM). **(D)** Absolute aortic diameters in cm for the murine PPE and local lenvatinib experiments (n=11 for PPE; n=5 for Lenva local; n=3 for PBS local; **=p<0.01; mean + SEM). **(E)** PPE+Sham re-operation shows a disrupted media with remaining CD31 and αSMA positivity, 4 weeks after initial aneurysm induction (magnification 10x/40x; scale bar 1mm).

Suppl. Fig. 2

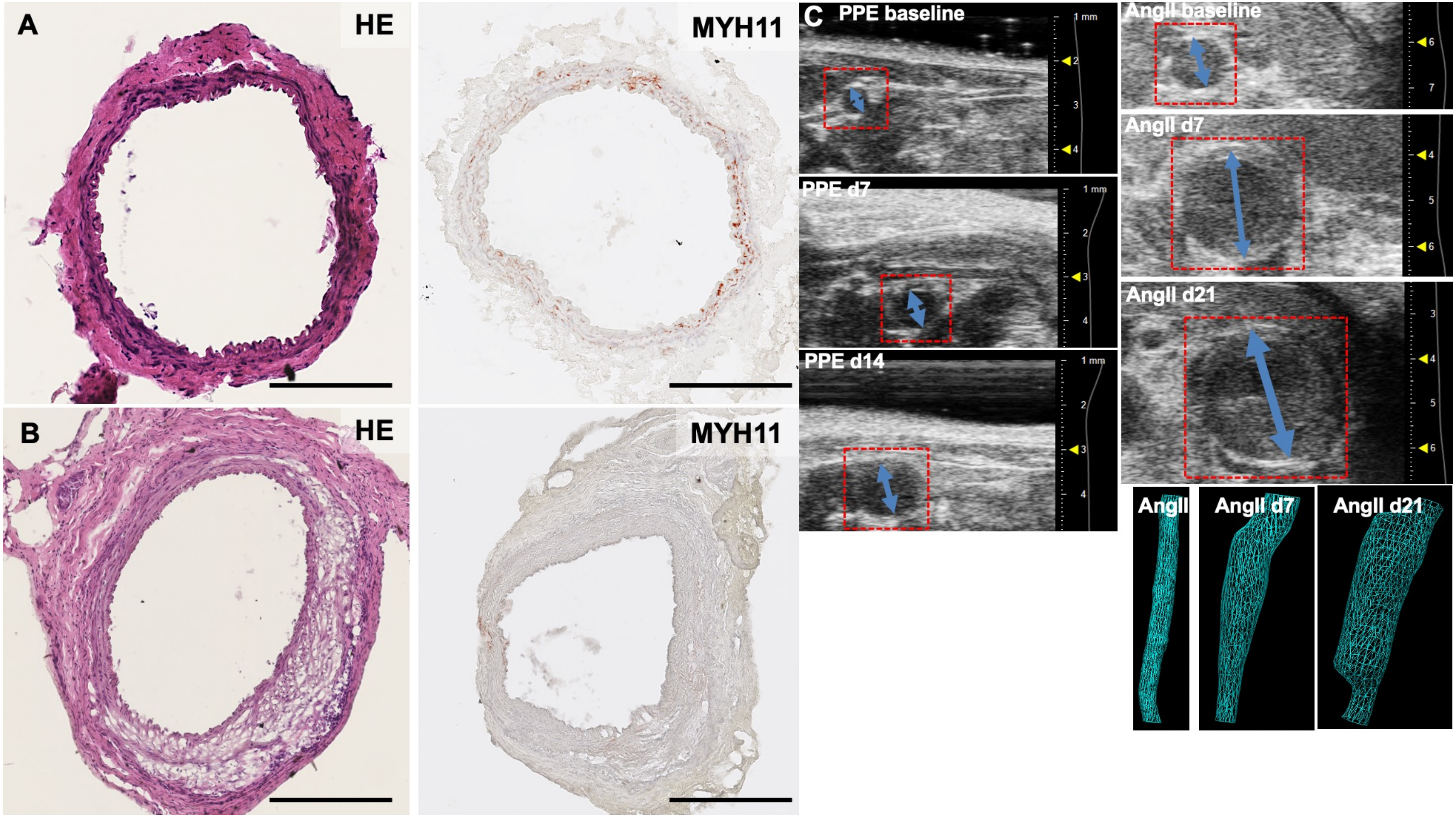

Suppl. Figure 2: Murine MYH11 phenotype staining and murine ultrasound: (A) Untreated control infrarenal aorta with HE staining and MYH11 immunofluorescence with red-brown target staining. **(B)** PPE aorta (day7 after induction) with altered tissue architecture and loss of MYH11 positivity. (scale bar 250µm) **(C)** Left panel: B mode ultrasound of the murine PPE model shows a gradual increase of the infrarenal aortic diameter from baseline to d7 and d14 (red dotted square indicates the area of interest; blue arrow indicates the aortic diameter at the maximum distance; scale bar on right side of each photograph). Right panel: ultrasound pictures from the AngII model showing diameter increase of the suprarenal aorta with visible intramural thrombus formation d21 (red dotted circle indicates the area of interest; scale bar on right side of each photograph). 3D reconstructions of the same animal at all three time points for volume calculation as presented by the Vevo 2100 Imaging System.

Suppl. Fig. 3

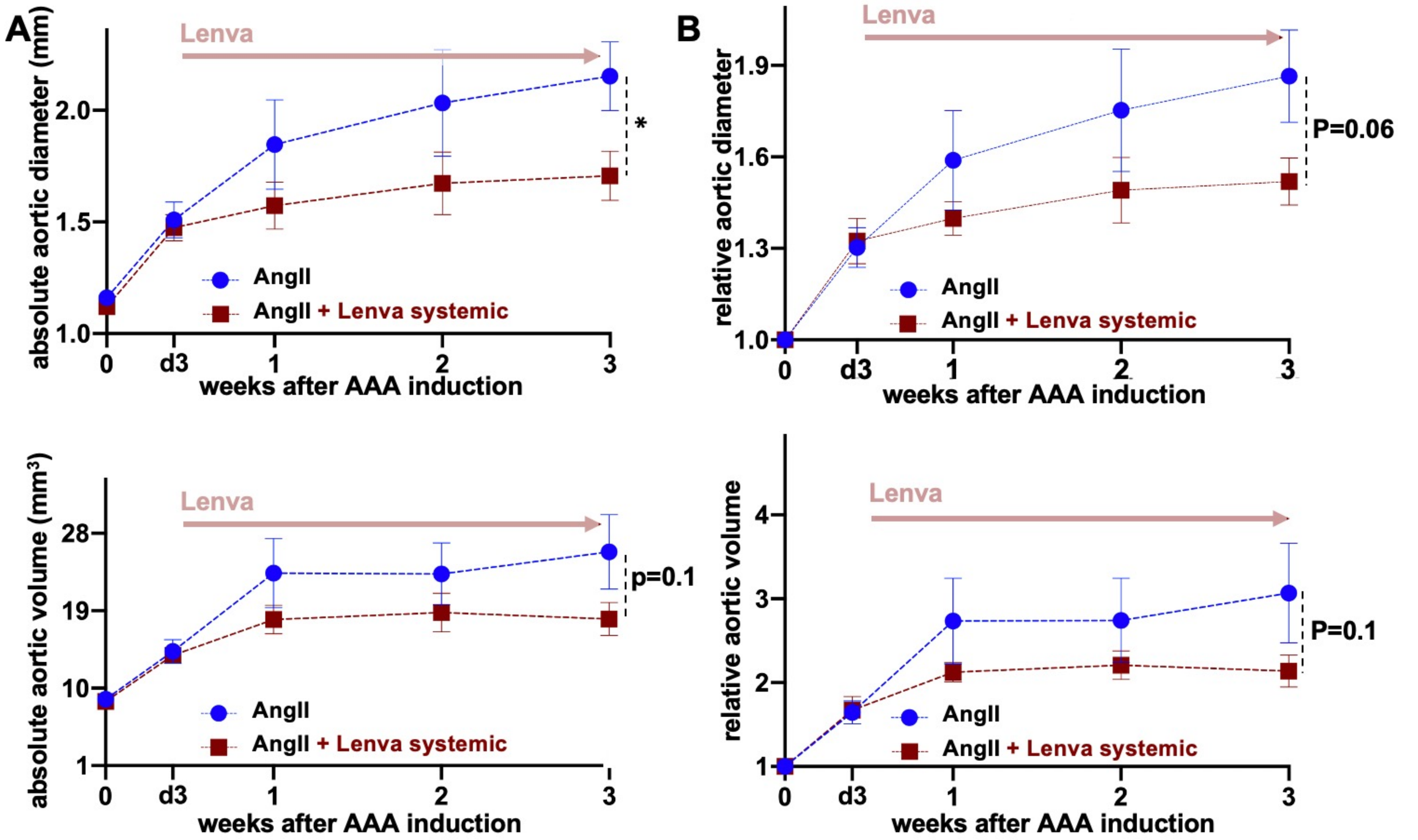

Suppl. Figure 3: Murine AngII-AAA data: Oral gavage of lenvatinib (dark red; n=6) continuously from day 4 (ultrasound at day 3) after AAA induction by Angiotensin II infusion. **(A)** Absolute aortic diameters and volumes in mm and mm^3 for the murine AngII and lenvantinib experiments (n=5 for AngII; n=6 for AngII + Lenva systemic; *=p<0.05; mean + SEM; Ang II induction started by implantation of osmotic minipump at day 0). **(B)** shows the relative aortic diameter and volume for the same experiment (relative to baseline aortic diameter/volume). (*p*-value calculations and detailed measurements in Suppl. Table I).

Suppl. Fig. 4

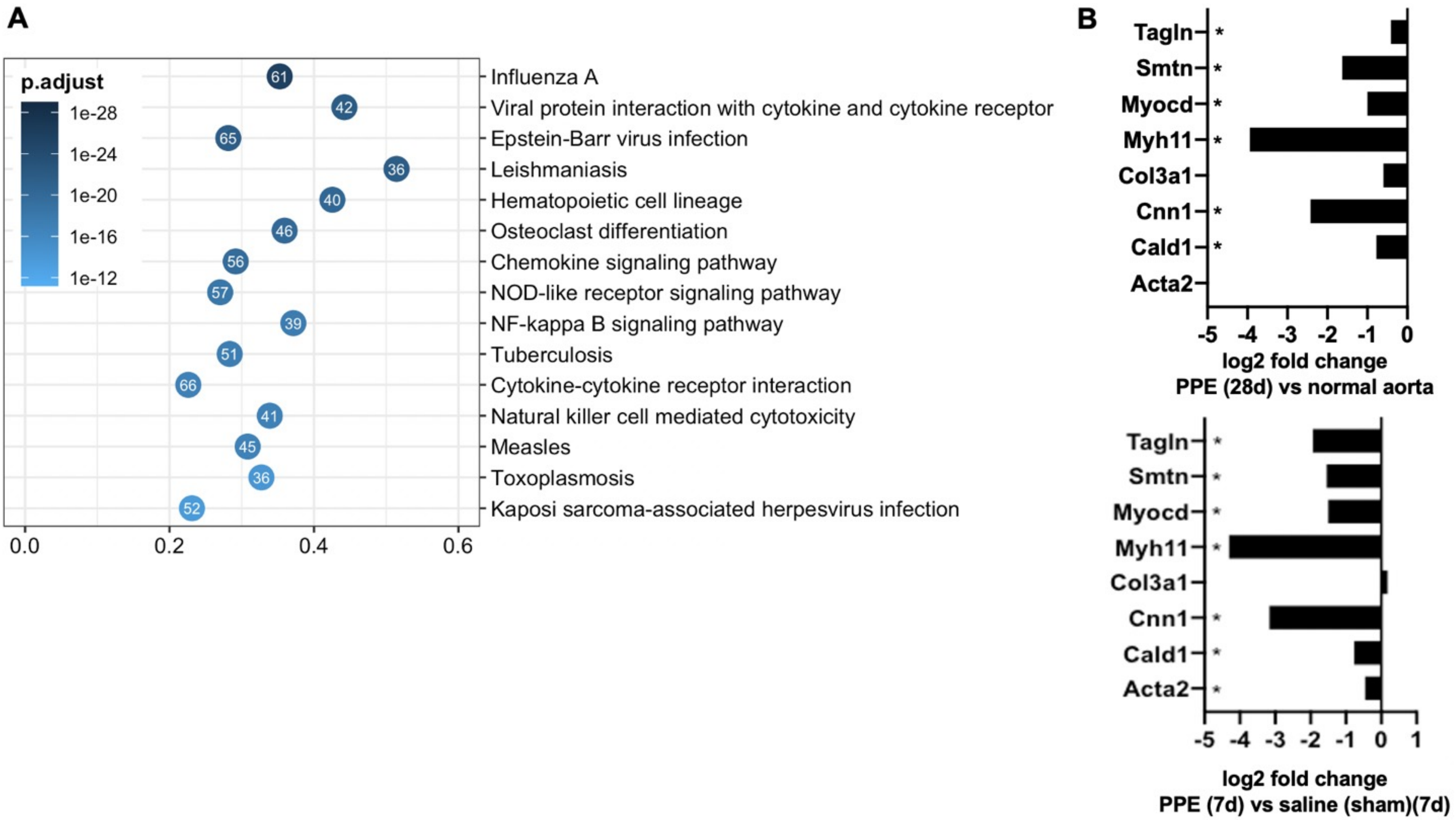

Suppl. Figure 4: PPE MTA pathway analysis: (A) KEGG-pathway gene overrepresentation analysis of significantly up-regulated genes (adjusted p-value < 0.05, Fc < -2) at day 7 post PPE compared to sham (saline) controls, showing baseline transcriptomic characteristics of the model at the timepoint of lenvatinib treatment initiation. Top 15 significantly affected pathways are shown (adjusted p value is given in shades of blue). **(B)** Subset of contractile marker gene expression analysis of PPE mice aortic tissue on day 28 after aneurysm induction compared to untreated control aortas based on own MTA array data (upper panel). Similar analysis of PPE mice aortic tissue on day 7 after aneurysm induction compared to sham-treated (saline) aortas (lower panel) (*=p<0.05).

Suppl. Fig. 5

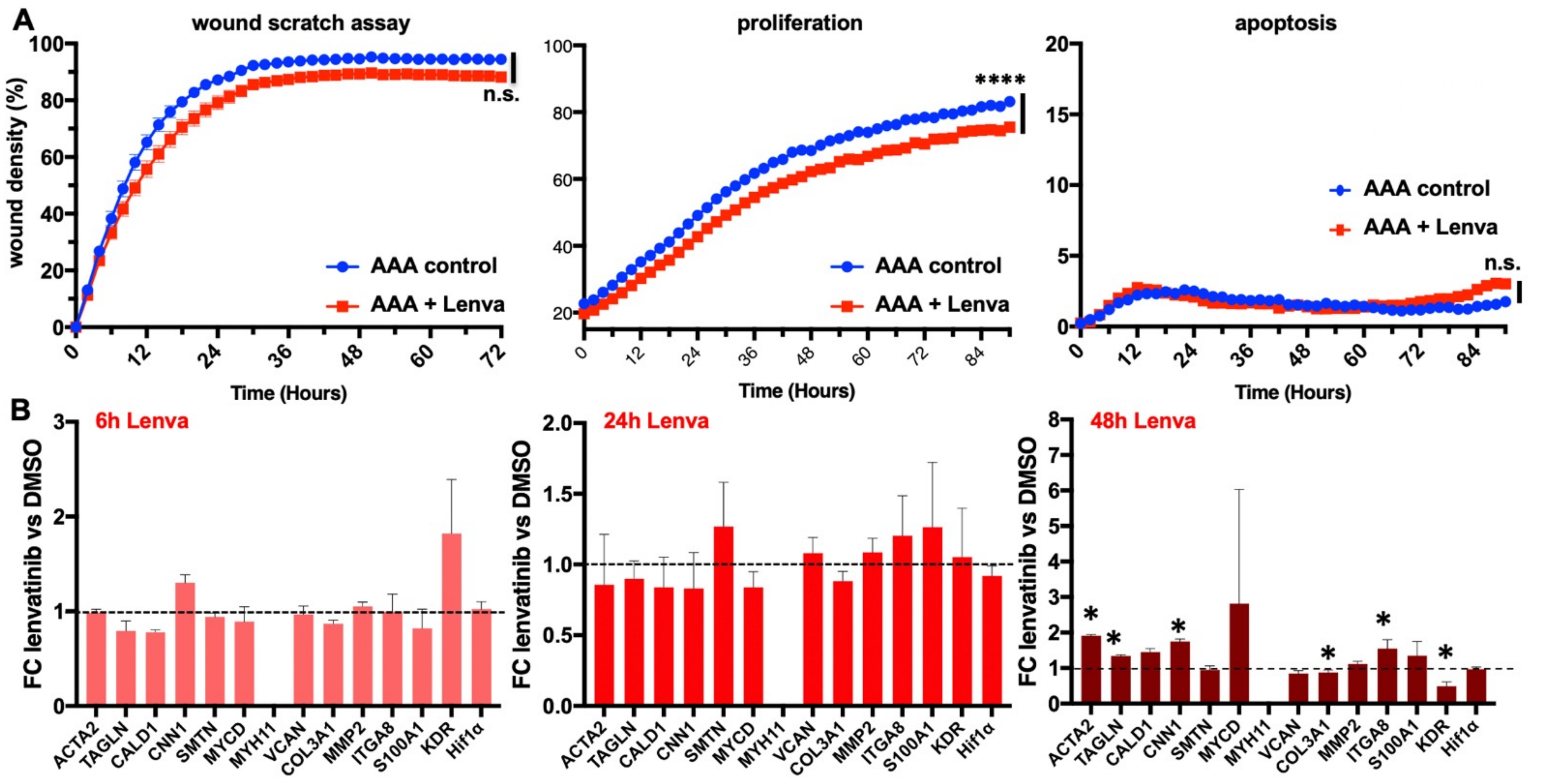

Suppl. Figure 5: Lenvatinib effect on AAA patient-derived SMCs #3: (A) Migration (left panel) proliferation (middle panel) and apoptotic (right panel) assays of AAA patient #3-derived SMCs exposed to lenvatinib. **(B)** A timely resolution of gene expression analysis at 6h, 24h, and 48h, assessing early and late effects of lenvatinib treatment on primary human AAA patient-derived VSMCs. (AAA control=treatment with 0.1% DMSO; *=p<0.05; ****=p<0.0001; for list of genes, primers and assays used, a detailed explanation is presented in Suppl. Table 4)

Suppl. Fig. 6

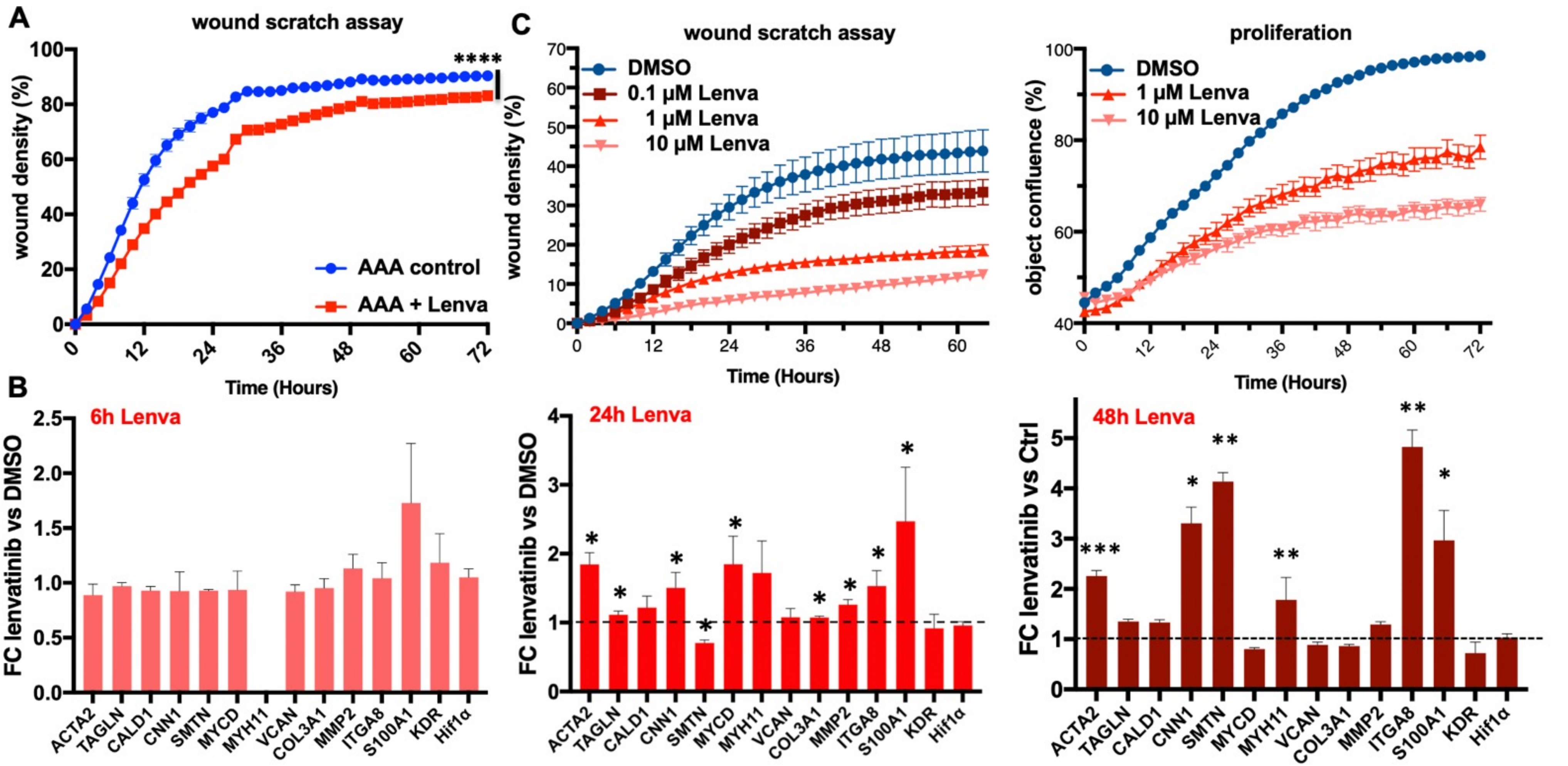

Suppl. Figure 6: Lenvatinib dose-finding experiments and its effect on AAA patient-derived cells #2: (A) Migration assays on AAA patient #2 derived SMCs. **(B)** A timely resolution of gene expression analysis after 6h, 24h, and 48h, assessing the early and late effects of lenvatinib treatment on primary human aortic cells. **(C)** Three doses of lenvatinib were evaluated by effects on migration (wound scratch assay) and proliferation (right panel). A dose of 0.1µM lenvatinib markedly reduced mobility and was thus chosen for all cell-based assays. (AAA control=treatment with 0.1% DMSO; *=p<0.05; ****=p<0.0001; full list of genes, primers and assays are displayed in Suppl. Table 4)

Suppl. Fig. 7

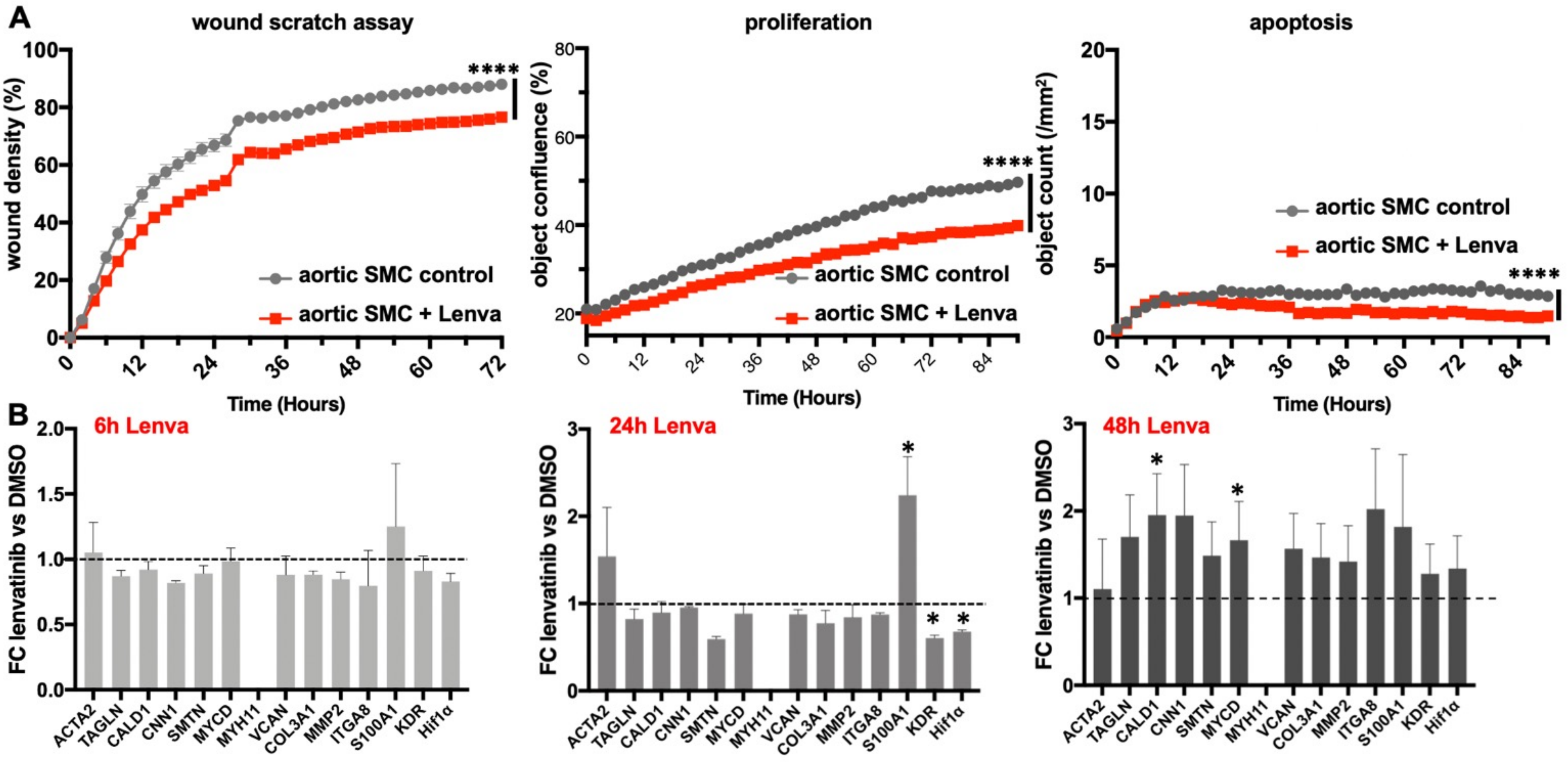

Suppl. Figure 7: Lenvatinib effect on healthy donor-derived SMCs (control cells): (A) Migration, proliferation and apoptosis assays upon lenvatinib treatment (from left to right panel). **(B)** A timely resolution of gene expression analysis at 6h, 24h, and 48h, assessing early and late effects of lenvatinib treatment on primary control VSMCs. (aortic SMC control=treatment with 0.1% DMSO; *=p<0.05; ****=p<0.0001; full list of genes, primers, and assays are shown in Suppl. Table 4)

Suppl. Fig. 8

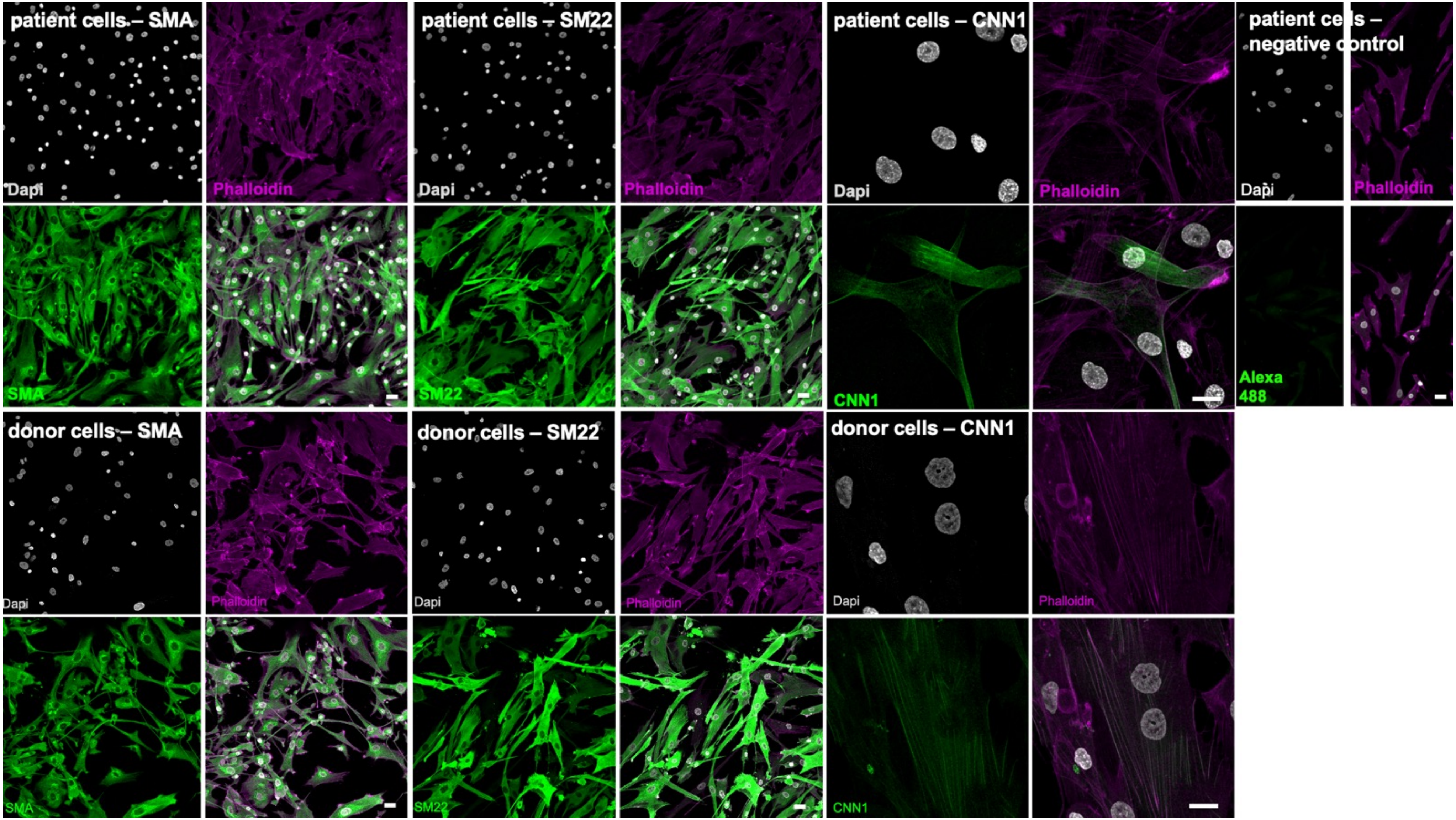

Suppl. Figure 8: Characterization of AAA patient-derived SMCs and healthy donor SMCs: Representative confocal laser scanning microscopy images of AAA patient-derived SMCs (patient #1 – upper panel) used for the previously described experiments. Staining for αSMA, SM22, CNN1, and zoomed area for CNN1 are shown sequentially, in presence of cytoskeleton marker G-actin filament (phalloidin). Negative control for the green channel reveals little to no auto fluorescence within the cells. (20x/40x magnification; scale bar: 100µm). Lower panel shows the same stains for donor aortic cells.

Suppl. Fig. 9

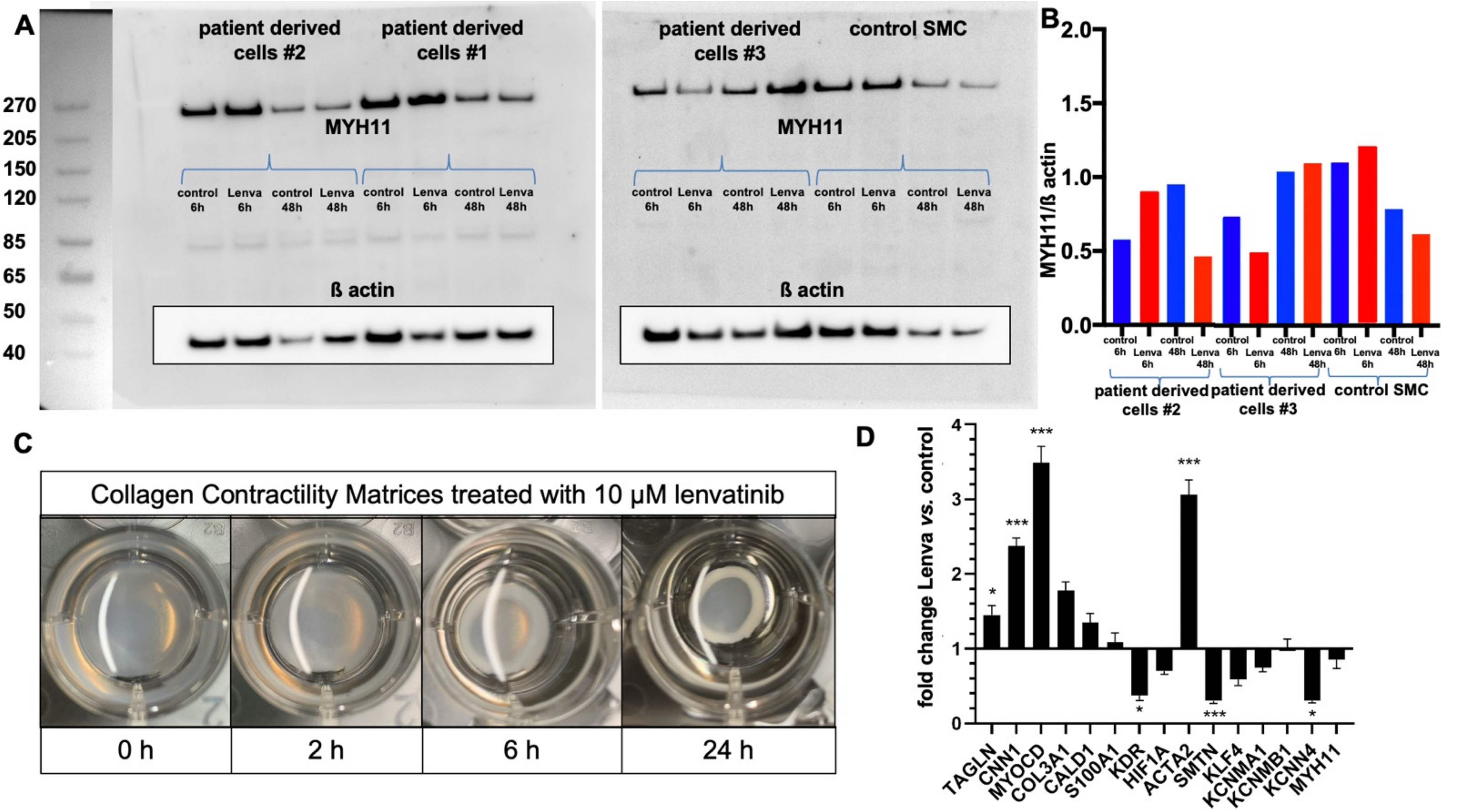

Suppl. Figure 9: MYH11 complete Western Blot images from primary human cells and contractility assay: (A) The two membranes show the staining of MYH11 in all 3 primary human AAA patient-derived cells (patient #1-3), and the healthy donor control cells. ß-actin serves as loading control and for normalization, shown as an inlay. Patient-derived cells #1 is blown-up and shown in Figure 4C. **(B)** Additionally, the quantification of the western blot images (MYH11 normalized to ß-actin) is presented for all conditions (control treatment 6h/48h and lenvatinib 6h/48h). **(C)** Representative images of collagen matrices from the collagen contractility assay after treatment with lenvatinib reveals a time-dependent contraction of the matrix from 0-24h. **(D)** The gene expression analysis at 24h time point of the contractility assay shows upregulation of contractile VSMC genes (i.e. *ACTA2, TAGLN* and *MYH11*) upon lenvatinib treatment (*=$p<0.05$; mean ± SEM) A subset of these genes is presented in Figure 4.

Suppl. Fig. 10

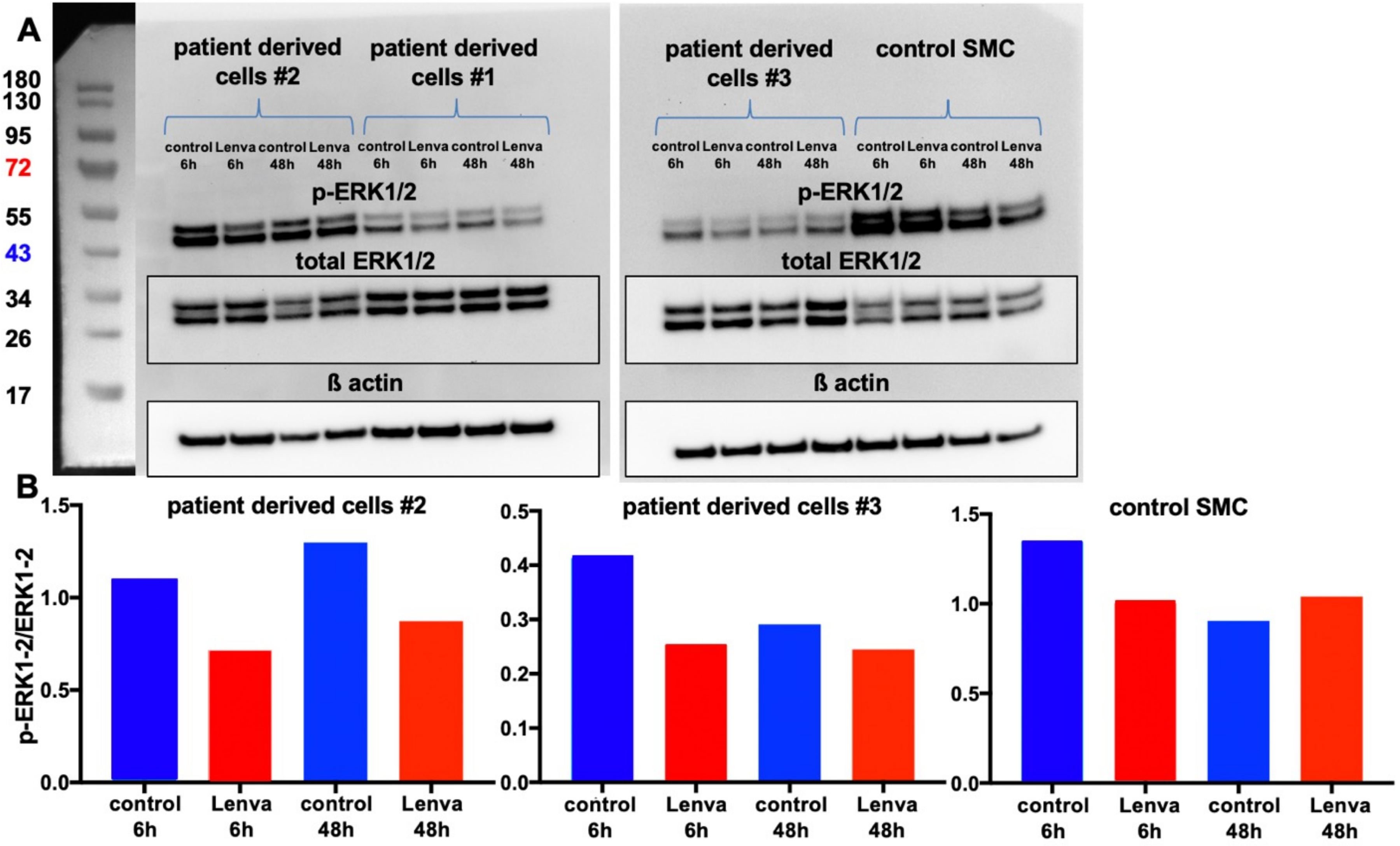

Suppl. Figure 10: ERK1-2/pERK1-2 complete Western Blot images: (A) The two membranes show the staining for pERK1-2/ERK1-2 for all 3 AAA patient-derived cells (#1-3) and the healthy donor control cells. Total ERK1-2 and ß-actin as loading control are shown as an inlay. One cell isolate (patient derived cells #1) is highlighted and presented in Figure 4D. **(B)** Additionally, the quantification of the Western Blot images (pERK1-2 normalized to ERK1-2) is shown for all conditions (control 6h/48h and lenvatinib 6h/48h).

Suppl. Fig. 11

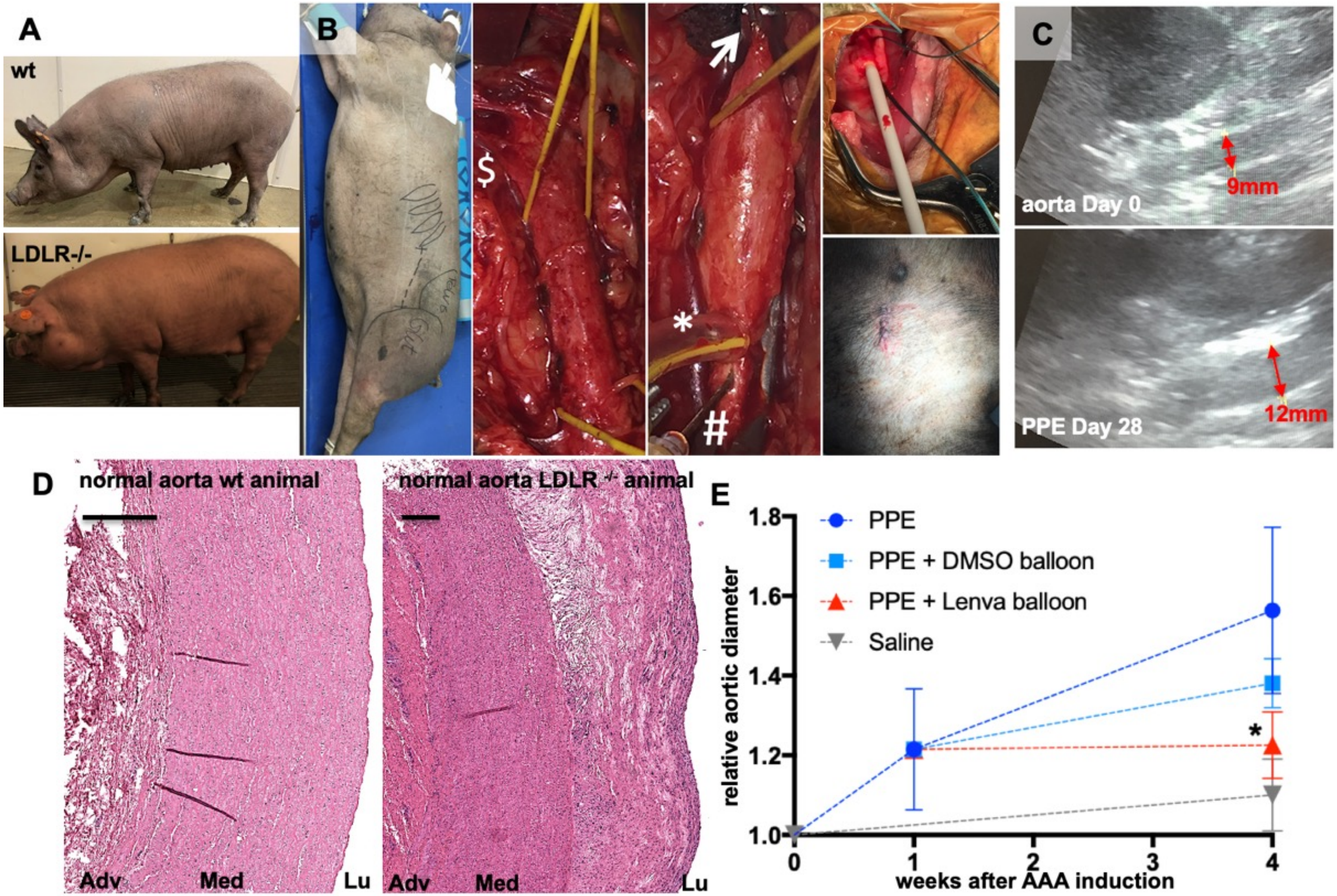

Suppl. Figure 11: PPE-AAA in Yucatan minipigs: (A) 12-months-old LDLR-/- (bottom) and wildtype (top) Yucatan minipigs demonstrating an obese phenotype upon gross appearance. **(B)** Pigs are placed on their right side and a left retroperitoneal approach exposes the infrarenal aorta from the lower renal artery ($) to the aortic trifurcation. After ligation of the lumbar arteries, a Satinsky clamp (arrow) is applied, and the isolated segment is pressure-perfused with elastase via a blunt 5G needle (#) secured by an additional vessel loop tourniquet (asterisk). For the angioplasty (7 days after AAA induction), femoral access is achieved by a longitudinal incision into the right groin (bottom image), and a 5F introducer sheath is inserted after distal ligation of the vessel (top image). **(C)** Abdominal B-mode ultrasound is used to measure diameters of the infrarenal aorta, based on leading-edge technique at baseline (top), and after 1 and 4 weeks after aneurysm induction. **(D)** HE staining indicates non-calcified plaque formation with foam cells and cholesterol depositions in the endothelium and media of LDLR$^{-/-}$ compared to wildtype aorta. Note the cell-rich infiltrate at the border of plaques. **(E)** Intraluminal saline perfusion (grey) instead of PPE (dark blue) does not lead to aortic diameter enlargement. Endovascular control (DMSO as vehicle only) coated balloon (light blue) treatment at day 7 after AAA induction showed a trend towards smaller diameter, however, only lenvatinib coated balloon angioplasty (red) resulted in a significant diameter reduction compared to PPE (*=p<0.05; mean ± SEM; PPE AAA induction at day 0; scale bar 200µm; Adv=adventitia; Lu=lumen; Med=Media).

Suppl. Fig. 12

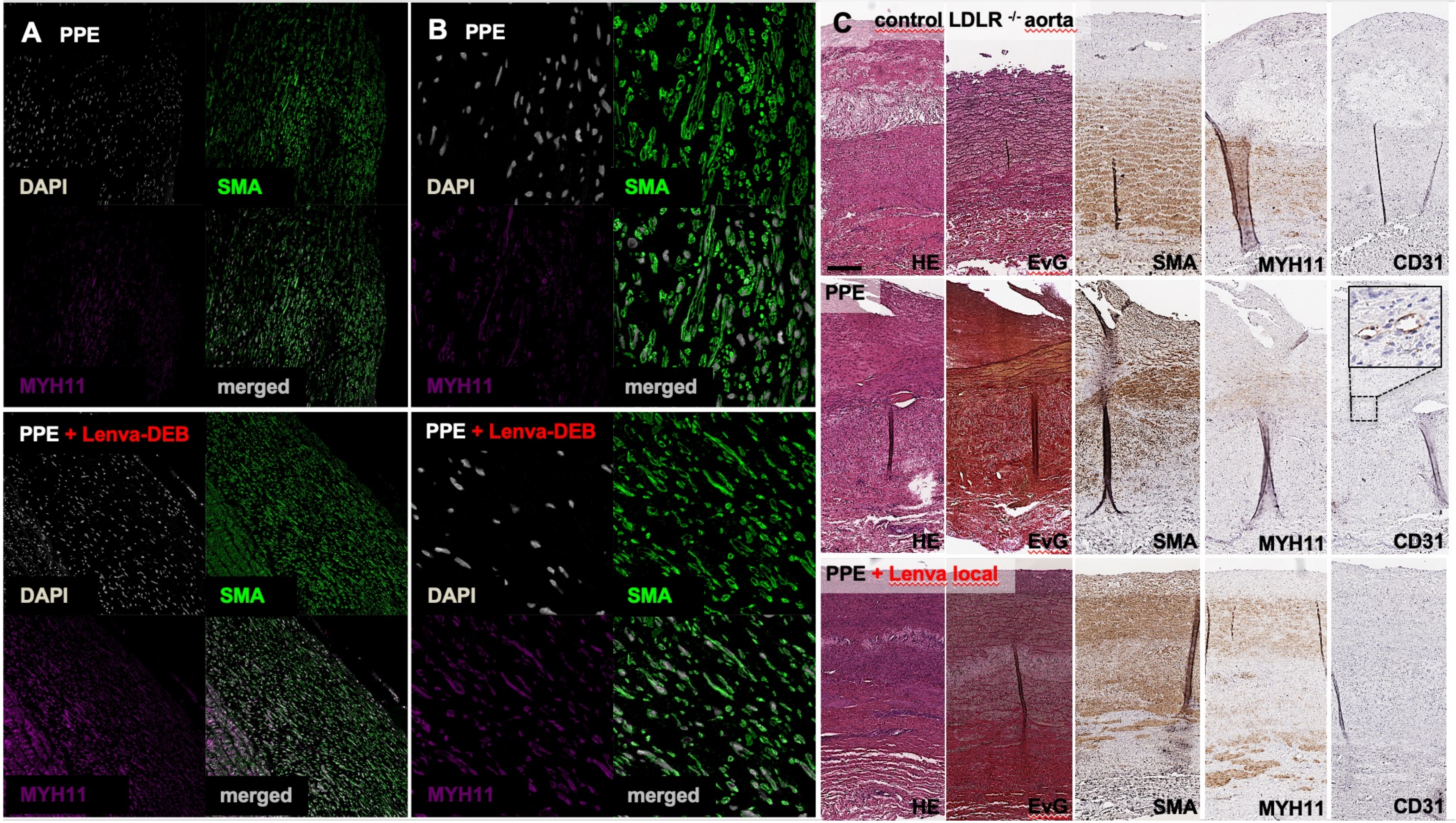

Suppl. Figure 12: Minipig low and high magnification double IF and conventional IHC: (A,B) Porcine double staining for αSMA and MYH11 of the aortic media shown at 20x magnification in relation to higher magnifications using digital zoom (Lumen always oriented to the right). The high magnification images are also shown in Figure 6 B. **(C, upper panel)** Aortas of LDLR-/- minipigs present a wall structure with parallel orientated elastic fibers in the media and plaque formation in the intima/media (HE and EvG stains) **(C, middle panel)** VSMCs in the media are positive for αSMA and MYH11, without indicating CD31 positivity. Upon PPE-AAA induction, elastic fibers become disrupted, and the parallel-layered structure of the media is vanished. Vast positivity for αSMA remains, whereas only a few cells show MYH11. **(C, highlighted window)** CD31-positive neovessels are detected in the media and the thickened adventitia. **(C, lower panel)** In animals with PPE-induced aneurysms and local lenvatinib-coated balloon treatment, a more organized and parallel-layered structure is recovered. Elastic fibers remain disrupted, but cells show positivity for αSMA and MYH11. CD31 positivity is not observed (magnification 5x; scale bar 100µm)

Suppl. Fig. 13

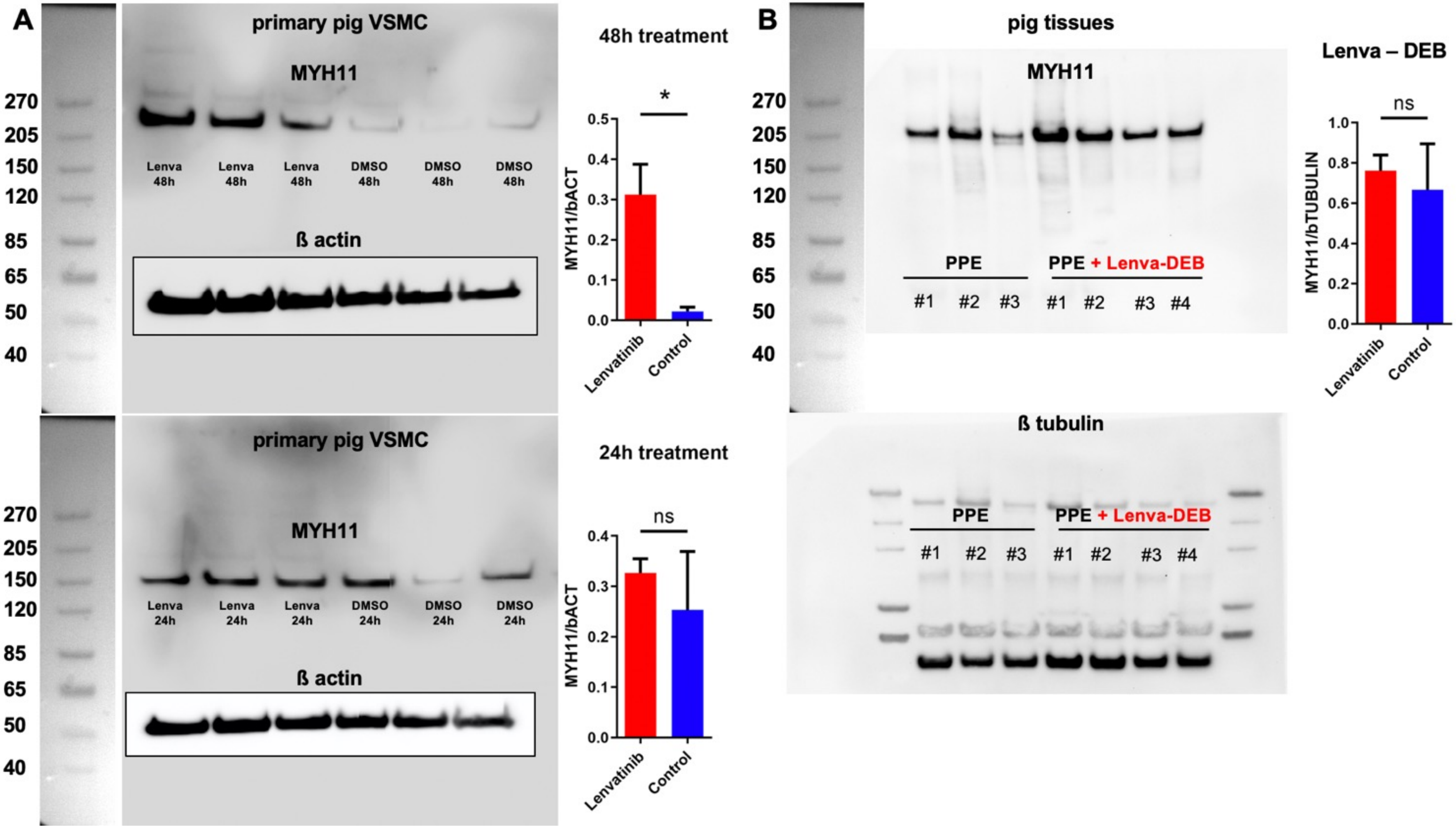

Suppl. Figure 13: Complete Western Blots and quantification from primary porcine SMC culture and pig tissue lysates: The two membranes show the staining for MYH11 (255kDa) after 24/48h incubation of primary porcine SMCs with lenvatinib or control treatment, respectively **(A)**. ß-actin (45kDa) is used for normalization and presented as an inlay. The 48h-time point is highlighted and presented in Figure 5F. **(B)** Additionally, the membranes and quantification of the staining intensity comparing MYH11 and ß-tubulin (55kDa) in lysates from pigs treated with the PPE + Lenva-DEB versus PPE only show a trend of higher MYH11 protein abundance. This membrane is also shown in Figure 5E.

Suppl. Fig. 14

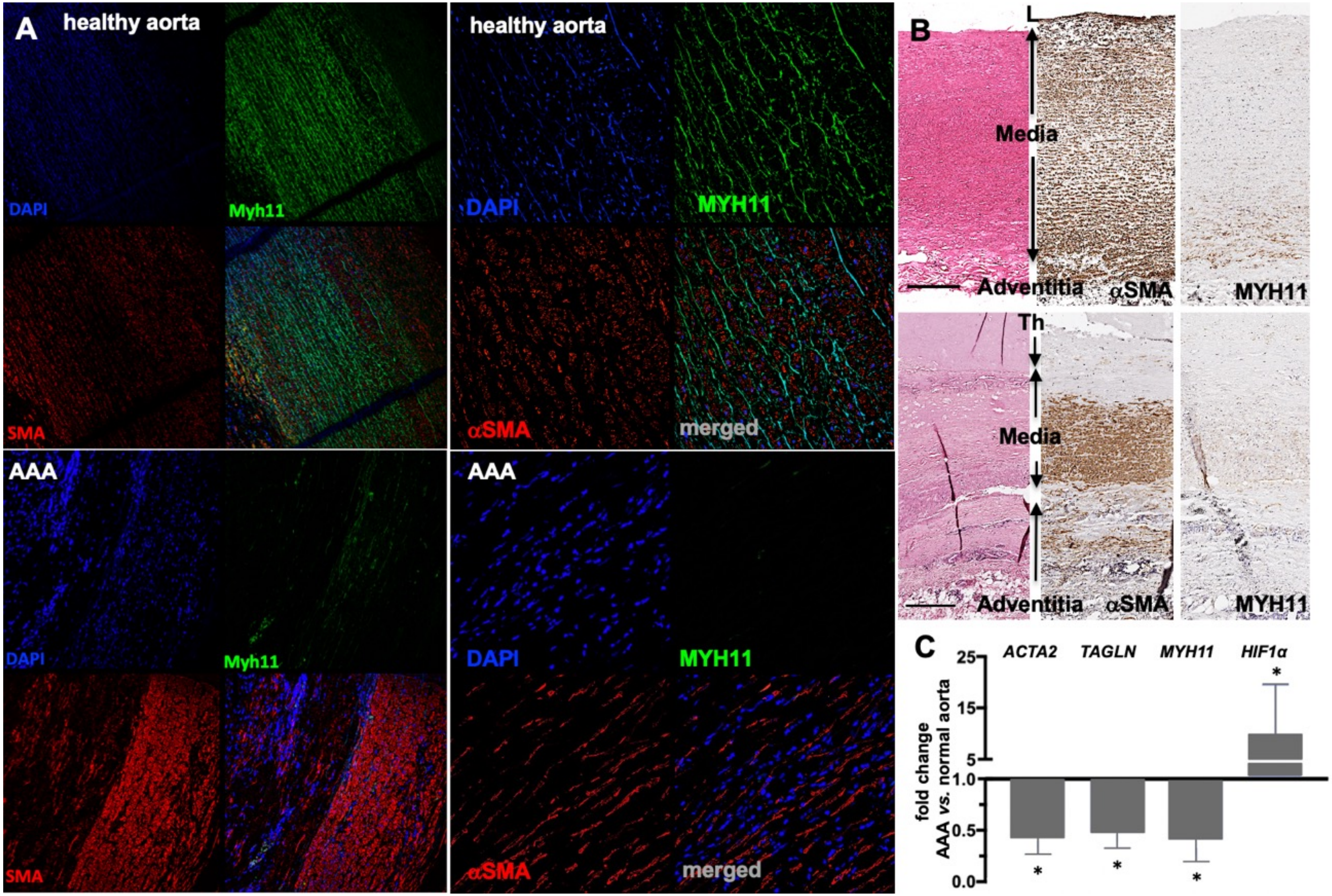

Suppl. Figure 14: Human AAA characterization I: immunofluorescence analysis reveals the distribution pattern of filamental αSMA and MYH11 in the aortic media, and a disruption of this co-expression in AAA in low and high magnification (20x/63x) **(A).** The non-aneurysmatic human infrarenal aorta has a densely organized media with approximately 30 layers of elastic fibers in parallel orientation with αSMA and MYH11 positive VSMCs **(B – upper panel)**. In AAA, massive disruptions in the media and in elastic fibers are detectable **(B – lower panel)**. αSMA positivity remains in the media, whereas MYH11 vanishes (Lu=Lumen; Adv=Adventitia; Med=Media; Th=Thrombus; magnification 2.5x/40x; scale bar 200μm). Gene expression in human AAA and non-aneurysmatic aortic tissues reveal indicators of the contractile VSMC phenotype (*ACTA2*, *TAGLN* and *MYH11*) significantly reduced, whereas *HIF1α* expression is enhanced (*=p<0.05; mean ± SEM) **(C)**.

Suppl. Fig. 15

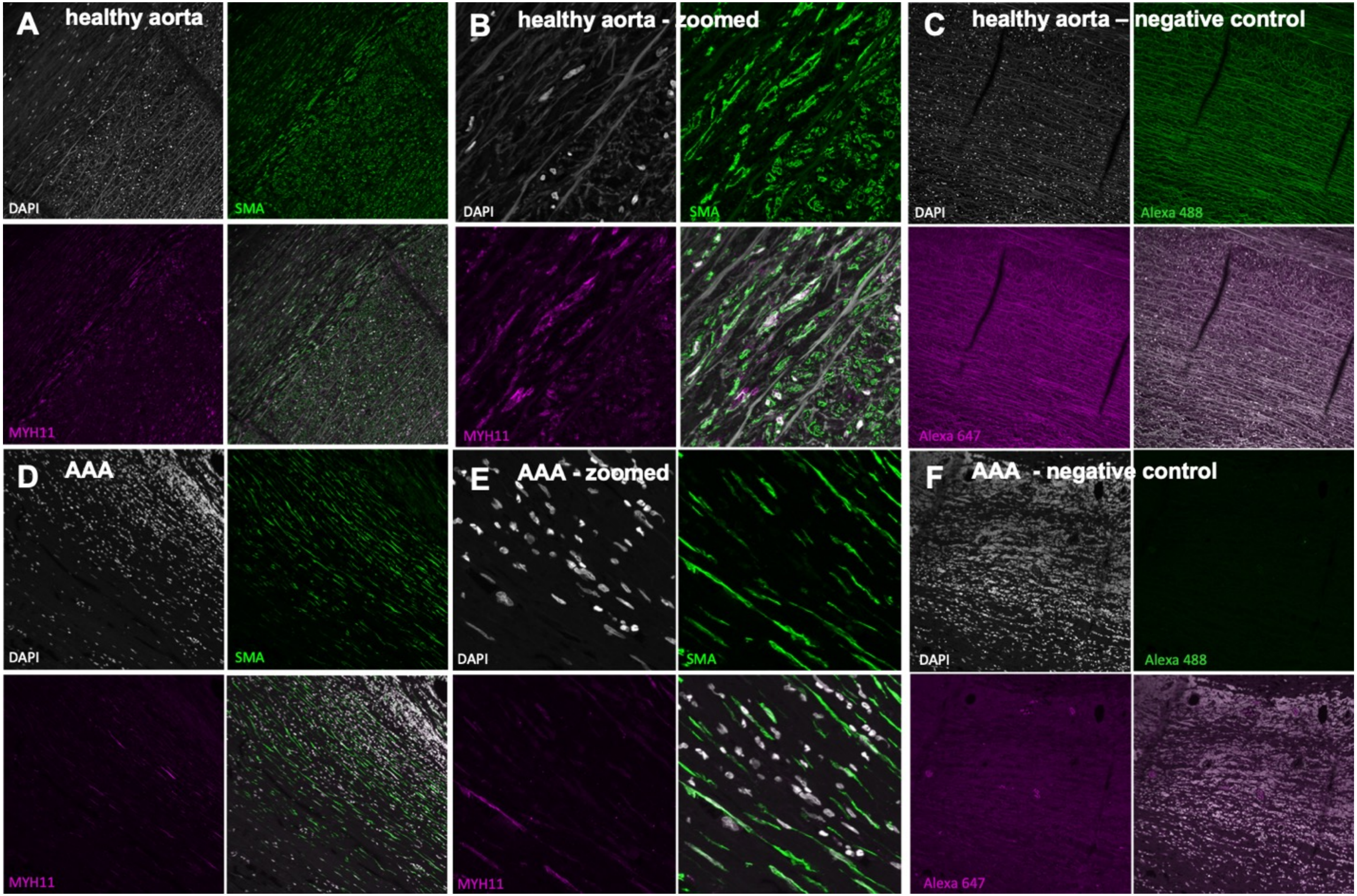

Suppl. Figure 15: Human AAA characterization II: Double immunofluorescence (different secondary antibodies as compared to Suppl. Figures 12, 14) in healthy (upper panel) and AAA (lower panel) aortas. In healthy tissues there is positive signals for αSMA and MYH11 (as highlighted in the zoomed area) whereas MYH11 is barely detectable in AAA specimens. The negative control with secondary antibody only reveals the unspecific background staining typical for vascular tissues. (scale bar: 100μm)

Online data supplement – Supplementary Material and Methods

Mouse PPE experiments:

S. **study approval section** for ethics declaration.

Anesthesia and PPE Procedure: Anesthesia was induced with isoflurane, while analgesia was provided with Temgesic (4mg/kg) before and after surgery. The aorta was isolated, and a temporary proximal and distal sutures were placed. Aneurysms were then induced with porcine pancreatic elastase (PPE; SigmaAldrich, Stockholm, Sweden) at a final concentration of 2 U/mL for 10 min by inserting a microcatheter into the aortic lumen. The aorta was then flushed with warm saline, and a single knot suture (10-0) was placed to seal the catheter entry site. After layered closure of the abdomen, the animal was allowed to recover on a heated mat.

Murine ultrasound: B-mode ultrasound was performed under general anesthesia with isoflurane using a Vevo 770 system and a Vevo Imaging Station (both Visualsonics, San Antonio, TX, USA). Our protocol for diameter assessment has been described before.(1) (detailed ultrasound data in Suppl. Table I)

Euthanasia and tissue processing: In all experiments presented in this study, animals were sacrificed 4 weeks after aneurysm induction using CO_2. Operations and images were performed using the operating microscope LEICA EZ4HD with a built in camera and the Leica Acquire Version 3.1 software (both Leica Microsystems, Buffalo Grove, IL, USA). Tissue samples were collected at the time of animal sacrifice. The dilated part of the aorta was carefully dissected and then cut in half. One part was kept for formalin fixation for histochemistry, and the second part was placed in RNAlater (SigmaAldrich, Stockholm, Sweden) and stored at -80°C awaiting further analysis. Control tissue was non-dilated infrarenal aorta from an additional six animals (also 10 week old male C57BL/6J wild type mice (Taconic, Biosciences, Hudson, NY, USA) sacrificed explicitly for this purpose.

Mouse AngII experiments:

S. **study approval section** for ethics declaration.

Anesthesia and AngII Procedure: 18 mice were purchased from Charles River (Sulzfeld, Germany). Ang-II (BACHEM, Switzerland) was dissolved in saline for the required dose of 1000 ng/kg/min at a pump distribution rate of 0.25 µl/h. The Alzet osmotic pumps (Model 2004, DURECT Corporation) were filled with Ang-II solution and conditioned over night at 37°C in saline. Animals were anesthetized and the Alzet osmotic pump was inserted into a subcutaneous pocket lateral of the spine, with the flow moderator facing downwards.

Murine ultrasound: For ultrasound measurements of aneurysms, the following settings of the Vevo 2100 Imaging System (FUJIFILM VisualSonics Inc., Toronto, ON, Canada) were applied: gain 30 dB, image depth 9 mm, image width 8 mm. Respiratory gating was set to 25% delay and a window of 50%; T1 50 ms were used for ECG trigger. After localization of the left renal artery, the MS550 transmitter was moved 6 mm cranially for automated scan of the suprarenal aorta. 157 imaging frames were generated over a scan distance of 12 mm with 0.076 mm step size. The suprarenal aortic volume was calculated in mm3 (over the monitored distance of 12

mm) with Vevo Lab 3.1.1 software; images of ECG-gated kilohertz visualization were used to additionally determine the maximum diameter of the aorta (inner-to-inner wall) at maximal blood flow as described before.(2) Independent measurements by three observers were averaged in volume and diameter calculations (Suppl.Fig.2).
Euthanasia and tissue processing: please see section above on PPE experiments.

Mouse RNA extraction:
Approximately 1mm long pieces of mouse aortic tissue were homogenized in Qiazol using Multi-Gen probes (Pro-Scientific, Stockholm, Sweden). RNA extraction was performed using the Qiagen miRNeasy Micro Kit (Qiagen, Sollentuna, Sweden) following the manufacturer's protocol. Total RNA concentration was assessed using a NanoDrop 2000 spectrophotometer (Thermo Fisher Scientific, Waltham, MA, USA). In the MTA analysis (Affymetrix, Santa Clara, CA, USA), samples from *n*=6 PPE, *n*=7 PPE+Lenvatinib, and *n*=5 control aortas were included with RIN numbers between 5.7-9.8. The MTA procedure was performed by the Center for Molecular Medicine (CMM) core facility at Karolinska Institutet, Stockholm, Sweden, according to the manufacturer's protocol.

Mouse Immunohistochemistry:
5*µ*m sections were stained for different targets with an overnight protocol with primary antibodies (Suppl. Table 3). Endogenous peroxidase activity was quenched with hydrogen peroxide for 10 min (Merck, Darmstadt, Germany). Target staining was done with the Vectastain ABC and the Peroxidase Substrate kit (both Vector Laboratories, Burlingame, CA, USA) after species-specific secondary antibody incubation. Counterstaining was performed using Mayer's hematoxylin (Carl Roth, Karlsruhe, Germany). Negative control, including incubation with phosphate buffered saline instead of primary antibody, was done for each antibody. Slides were then imaged with a Keyence BZ9000 microscope (Keyence, Kyoto, Japan) and scanned with a NanoZoomer 2.0-HT Digital slide scanner C9600. Pictures were analyzed with the NDP.view2 software (both Meyer Instruments, Hamamatsu, Japan). Image analysis was performed with AZ AnalyserII software (Keyence), as well as Imaris (Oxford Instruments, Zurich, Switzerland) and Fiji ImageJ for high power field (HPF) analysis of cell counts.
Mouse OCT frozen sections were fixed in 4% paraformaldehyde PBS buffered solution and incubated with primary Ki-67 antibodies (Abcam, ab16667) 1:200 overnight. To visualize staining TSA Plus Cyanine 5 Evaluation Kit (PerkinElmer, NEL745E001KT) was applied in 1:50 dilution. Sections were mounted with ProLong Gold (ThermoFisher), and images were taken using a confocal Leica SP5 microscope.
For each mouse aorta, 2 slides were stained for the respective antibody and out of these, the most representative image was chosen for presentation.

Overview animal experiments (animal numbers):

**mouse experiments**		
group		*# animals*
untreated control aorta		5
PPE	PPE only	11
	+ systemic Lenvatinib treatment	7
	+ local Lenvatinib treatment	5
	+ local sham (PBS) treatment	3
AngII	w/o oral gavage	5 completed<br>*4 ruptures*
	+ oral gavage Lenvatinib	6 completed<br>*3 ruptures*
**pig experiments**		
PPE	PPE only	7
	+ local Lenvatinib DEB	4
	+ local sham (DMSO) DEB	3
untreated control aorta		3

Minipig PPE-AAA experiments:

S. **study approval section** for ethics declaration.

Anesthesia: The basic anesthesia regime included: Sedation by combination of ketamine 15mg/kg, azaperone 2mg/kg and atropine 0.1mg/kg intramuscularly. An intravenous catheter was placed in the lateral auricular vein. Induction of anesthesia by propofol (4-8 mg/kg) and oral intubation with a 7.0-8.5 mm cuffed endotracheal tube. Anesthesia was maintained by continuous infusion of propofol (2.5-7mg/kg/h). Pigs were mechanically ventilated in volume-controlled mode (6-10ml/kg) with 40-50% oxygen (1-2l/min) and breathing rate 11-13x/minute. Parameters were adjusted to maintain normocapnia (end-tidal CO2: 35-45mmHg). Saline was infused at a maintenance rate of ~10 ml/kg/h. Intraoperative monitoring included reflex status, heart rate and peripheral arterial oxygen saturation. External warm-air supply was provided. A single-shot cefuroxim (750mg) was administered before skin incision. For multimodal analgesia during the surgery, metamizole (50mg/kg), carprofen (4mg/kg) and fentanyl (0.001–0.01mg/kg) were administered.

Ultrasound: Ultrasound was performed immediately after anesthesia before any further procedure by 2 independent examiners (A Busch and/or C Knappich) using a GE Logiq S7 system equipped with a 4-5MhZ abdominal ultrasound probe (GE, Frankfurt, Germany). The aortic trifurcation and the renal arteries were identified and aortic diameter was acquired using the leading edge method in transverse and longitudinal sections at the point of maximum diameter (Suppl. Figure 9C; Suppl. Table II).(3)

Postoperative analgesia: After each surgery, carprofen (4mg/kg) orally for at least 5 days and a single dose of buprenorphine (0.005–0.1mg/kg) shortly before the end of anesthesia were given to each pig. After the first surgery (induction of aneurysm), the administration of buprenorphine was continued for at least 2 days.

Blood sampling: Blood sampling (10ml) under anesthesia was performed at day 0, day 8±2 (catheterization) and day 28 ±3 (euthanasia) *via* the lateral auricular vein or femoral artery. Parameters: Blood cell count, alkaline phosphatase, AST, ALT, γ-GT, GLDH, bilirubin total, creatinine, urea, albumin, total protein, glucose (Suppl. Table III).
Euthanasia: After a final ultrasound examination the animals were sacrificed under general anesthesia with pentobarbital (>50mg/kg) and 40ml of 1M KCl solution.

Minipig tissue collection and processing:
Directly after euthanasia, the incisional wounds were reopened and access to the infrarenal aorta was gained in order to identify the area of the aneurysmatic lesion. Samples were collected from the AAA, the surgically untouched pararenal aorta (no additional animals were sacrificed for control tissue sampling), the common carotid, the iliac and the coronary arteries, the aortic valve, the left ventricle myocardium, a jugular lymph node, liver, kidney, spleen and lung parenchyma, as well as whole blood in EDTA and serum. All samples were divided for RNA isolation and snap frozen in liquid nitrogen and formalin fixation.

Minipig RNA extraction and qPCR experiments:
Approx. 3mm sections of aortic tissue were homogenized with the Bio-Gen PRO200 Homogenizer and Multi-Gen 7XL Probes (PRO Scientific, Oxford, CT, USA) after adding Qiazol. RNA extraction was performed using the miRNeasy Mini Kit (Qiagen, Hilden, Germany), according to the manufacturer's protocol and total RNA concentration was determined with a NanoDrop2000c (Thermo Fisher Scientific, Waltham, MA, USA).
300ng RNA were reverse-transcribed using the High-Capacity RNA-to-cDNA Kit (Thermo Fisher Scientific, Waltham, MA, USA). mRNA expression was measured based on qRT-PCR using TaqMan FAM labelled assays. Amplification was performed on a QuantStudio 3 PCR System (Thermo Fisher Scientific, Waltham, MA, USA) and all samples were normalized to RPLP0 (Ribosomal Protein Lateral Stalk Subunit P0). RNA expression was calculated by the ΔΔCt method and displayed as fold change compared to untreated pig aortas proximal to the surgically treated segment with a relevant distance.

Minipig immunohistochemistry:
2*µ*m sections of paraffin-embedded tissue were mounted on SuperFrost slides (Thermo Fisher Scientific, Waltham, MA, USA) and standard hematoxylin-eosin (HE) and Elastica van Gieson (EVG) stains were performed.
For immunohistochemistry (IHC), sections were mounted on 0.1% poly-L-lysine (Sigma-Aldrich, St. Louis, MO, USA) pre-coated SuperFrost Plus slides (Thermo Fisher Scientific, Waltham, MA, USA). For antigen retrieval the slides boiled in a pressure cooker with 10nM citrate buffer (distilled water with citric acid monohydrate, pH 6.0), and endogenous peroxidase activity was blocked with 3% hydrogen peroxide. Samples were incubated with primary antibodies (Suppl. Table 3) diluted in Dako REAL Antibody Diluent (Dako, Glostrup, Denmark). Slides were then treated

with biotinylated secondary antibodies and target staining was performed with peroxidase conjugated streptavidin and DAB chromogen (Dako REAL Detection System Peroxidase/DAB+, Rabbit/Mouse Kit; Dako, Glostrup, Denmark). Mayer's hematoxylin (Carl Roth, Karlsruhe, Germany) was used for counterstaining and appropriate positive and negative controls were performed for each antibody. All slides were scanned with an Aperio AT2 (Leica, Wetzlar, Germany), and images were taken with the Aperio ImageScope software (Leica, Wetzlar, Germany).

Primary porcine SMC isolation:

Porcine aorta material of healthy landrace pigs was harvested and stored in complete DMEM/F12 Medium (Sigma Aldrich, Taufkirchen, Germany containing 5% FBS and 1% PS). The tissue was placed in a sterile petri dish and washed with PBS. Adventitia and intima were removed, and the remaining media was cut into small pieces using a sterile scalpel. These were placed in digestion medium (1.4mg/ml Collagenase A, Roche, Mannheim, Germany, in complete DMEM/F12 Medium) in a humidified incubator at 37°C and 5% CO2 for 4-6 h. Cells were strained using a 100µm cell strainer to remove debris. After 2 washing steps (centrifuge 400g, 5 min; discard supernatant, re-suspended in 15ml complete DMEM/F12 Medium) cells were re-suspended in 7ml complete DMEM/F12 Medium and placed in a small cell culture flask in a humidified incubator at 37°C and 5% CO2. Medium was changed every other day. After 1 week the medium was replaced by SMC Growth Medium (PeloBiotech, Planegg, Germany). Upon confluence, cells were stored in liquid nitrogen or processed immediately. Cells were used between passages 3-7.

Incubation of pSMC with Lenvatinib and Western Blot:

Cells: Cells were placed in triplicates in 25cm2 flasks and treated with 0.1µM lenvatinib in OptiMEM (Thermo Fisher, USA, Lenvatinib-treatment) or 0.1% DMSO in OptiMEM (control-treatment) for 24h and 48h. Cells were washed with ice-cold PBS and harvested with 250µl freshly prepared complete RIPA Buffer (RIPA Lysis and Extraction Buffer, Thermo Fisher, USA) containing Phosphatase Inhibitor Cocktail 2 and 3 (Sigma Aldrich, USA) and Halt Protease Inhibitor Cocktail (ThermoFisher). After homogenization with a pistil and centrifugation for 20 min (14.000rpm at 4°C), the supernatant was frozen down at -80°C in aliquots of total protein lysate. Total protein concentration was measured using the Pierce BCA Protein Assay Kit (Thermo Fisher), following the manufacturer´s instruction.

Western Blot for MYH11: 5µg of protein of each sample was denatured and reduced at 70°C for 10min, then separated in a NuPage 3-8% Tris Acetate-Gel and transferred onto Trans-Blot Turbo Mini-Size LF-PVDF Membranes (BioRad, USA). The blots were blocked with 5% BSA in Tris-buffered saline+0.1%Tween-20 for 1h, followed by overnight incubation with the primary Rabbit Anti-smooth muscle Myosin heavy chain 11 antibody (ab53219, Abcam, United Kingdom) in TBS-T + 5% BSA. After washing with TBS-T, blots were incubated with anti-rabbit IgG H&L HRP (horseradish peroxidase)-conjugated secondary antibody (ab205718, Abcam, United Kingdom) and visualized using ECL Prime Western Blotting Detection Reagent

(RPN2236) in the c600 System from Azure Biosystems (Dublin, CA, USA), cSeries Capture Software v2.1.4.0731

Wester Blot for ß-actin (loading control): 5µg of protein of each sample (exact same batch as for MYH11 WB) was denatured and reduced at 70°C for 10min, then separated in a Bolt 4-12% Bis-Tris Plus Gel (Thermo Fisher) and transferred onto Trans-Blot Turbo Mini-Size LF-PVDF Membranes (BioRad, USA). The blots were blocked with 5% milk powder in Tris-buffered saline+0.1% Tween-20 for 1h, followed by overnight incubation with the primary antibody against ß-actin (A1978, Sigma Aldrich, Taufkichen) in TBS-T+5%milk. After washing with TBS-T, blots were incubated with anti-mouse IgG H&L HRP (horseradish peroxidase)-conjugated secondary antibody (ab205719 Abcam, United Kingdom) and visualized using ECL Prime Western Blotting Detection Reagent (RPN2236) in the c600 System from Azure Biosystems (Dublin, CA, USA), cSeries Capture Software v2.1.4.0731.

The blots were quantified using Fiji ImageJ Software.

Primary human AAA cells RNA isolation and qPCR:

Cells were placed in 6-well plates (triplicates each) and treated with 0.1*µ*M lenvatinib in OptiMEM (Thermo Fisher, USA, Lenvatinib-treatment) or 0.1% DMSO in OptiMEM (control-treatment) for 6, 24 and 48h. Cells were washed with PBS and harvested with 350*µ*l RLT Lysis Buffer (Qiagen, Netherlands). Total RNA was isolated using the RNeasy Mini Kit (Qiagen, Netherlands) according to manufacturer´s instruction. RNA concentration and purity was assessed using a NanoDrop system. Next, first strand cDNA synthesis was performed using the High-Capacity-RNA-to-cDNA Kit (Applied Biosystems, USA), following the manufacturer's instruction.

Quantitative real-time TaqMan PCR was then performed using primers for the following genes: Tagln, Cnn1, Myocd, Itga8, Col3A1, Cald1, S100A1, Kdr, Vcan, Hif1a, Acta2, Smtn and Mmp2 (detailed primer description in Suppl. Table 4). qPCR was run on a QuantStudio5 Cycler (Applied Biosystems, USA), using 384 well plates. Gene expression was normalized to Rplp0 and GAPDH, and quantified with the $2^{\Delta\Delta Ct}$ method.

For Myh11, SYBR Green based quantitative real-time PCR was performed. PCR was run on a QuantStudio5 Cycler, using 384 well plates. Gene expression was normalized to Rplp0 and GAPDH and quantified with the $2^{\Delta\Delta Ct}$ method.

Cytoskeleton staining (integrity) of primary human cells:

Human primary SMCs isolated AAA biopsies (from three different patients) were stained by immunofluorescence for the typical SMC markers (SM22-SMA-CNN1) as well as for F-actin with phalloidin and compared to commercially available SMC (from healthy donor). Patient-derived SMCs show a similar expression pattern for the utilised markers, to the control SMCs. Protein lysates from three different patient-derived cells were also analysed for the presence of SM22 protein by western blot.

Human tissue biobank:

Human aortic aneurysm samples and control aortic wall samples (non-aneurysmatic) were taken from the Munich Vascular Biobank described previously upon patients'

informed consent.(4) The biobank is approved by the local Hospital Ethics Committee (2799/10, Ethikkommission der Fakultät für Medizin der Technischen Universität München, Munich, Germany) and in accordance with the Declaration of Helsinki. Briefly, AAA samples were acquired during open repair from the left anterior wall of the aneurysm, control, non-aneurysmatic samples, and included donor patients from kidney transplants and samples from PAOD procedures - all from the infrarenal abdominal aorta. Samples were divided for, both, formalin-fixation and paraffin embedding, and snap frozen in liquid nitrogen until further use.

Immunohistochemistry human tissue:
2*µ*m sections of paraffin-embedded human aortic samples were mounted on SuperFrost slides (Thermo Fisher Scientific, Waltham, MA, USA) for HE and EvG stains. For IHC, sections were stained with different target antibodies (details in Suppl. Table 3), following the protocol described previously for minipig sections. Slides (including immunohistochemistry) were scanned with an Aperio AT2 (Leica, Wetzlar, Germany), and images were taken with the Aperio ImageScope software (Leica).

Human immunofluorescence (IF) double staining:
4*µ*m sections of paraffin-embedded human aortic samples were mounted on pre-coated SuperFrost Plus slides. Antigen retrieval and blocking of peroxidase activity was performed as described previously for IHC. In addition, slides were blocked with 5% horse serum for 30 min and all antibodies were diluted in 5% horse serum. Two primary and appropriate secondary antibodies (Suppl. Table IV) were added subsequently to the slides, with the primary antibody of interest (MYH11) being incubated overnight at 4°C, and the following primary antibody for co-staining (aSMA) for 1h at room temperature. Secondary antibodies were incubated for 1h each. Auto-fluorescence quenching, counterstaining, and imaging were performed as described in the pig double IF section.

Human tissue RNA extraction and qPCR:
Tissues were cut in ~50mg pieces on dry ice. Tissue was homogenized in 700*µ*l Qiazol lysis reagent and total RNA was isolated using the miRNeasy Mini Kit (Qiagen, Netherlands) according to manufacturer´s instruction. RNA concentration and purity were assessed using the NanoDrop system described above. RIN number was assessed using the RNA Screen Tape (Agilent, USA) in the Agilent TapeStation 4200. Next, first strand cDNA synthesis was performed using the High-Capacity-RNA-to-cDNA Kit (Applied Biosystems, USA), following the manufacturer's instruction.
Quantitative real-time TaqMan PCR was then performed using primers for the following genes: Tagln, Cnn1, Myocd, Itga8, Col3A1, Cald1, S100A1, Kdr, Vcan, Hif1a, Acta2, Smtn and Mmp2 (Suppl. Table 4). qPCR was run on a QuantStudio5 Cycler (Applied Biosystems, USA), using 384 well plates. Gene expression was normalized to Rplp0 and Gapdh, and quantified with the 2^ΔΔCt method.

For Myh11, SYBR Green based quantitative real-time PCR was performed. PCR was run on a QuantStudio5 Cycler using 384 well plates. Gene expression was normalized to Rplp0 and Gapdh and quantified with the $2^{\Delta\Delta Ct}$ method.

Protein isolation and Western Blot:

Cells: Cells were placed in 75cm^2 flasks and treated with 0.1μM lenvatinib in OptiMEM (Thermo Fisher, USA, Lenvatinib-treatment) or 0.1% DMSO in OptiMEM (control-treatment) for 6 and 48h. Cells were washed with ice-cold PBS and harvested with 750μl freshly prepared complete RIPA Buffer (RIPA Lysis and Extraction Buffer, Thermo Fisher, USA) containing Phosphatase Inhibitor Cocktail 2 and 3 (Sigma Aldrich, USA) and Halt Protease Inhibitor Cocktail (ThermoFisher). After homogenization with a pistil and centrifugation for 20 min (14.000rpm at 4°C), the supernatant was frozen down at -80°C in aliquots of total protein lysate. Total protein concentration was measured using the Pierce BCA Protein Assay Kit (Thermo Fisher), following the manufacturer´s instruction.

Western Blot for signaling cascade: 10μg of protein from each sample was denatured and reduced at 70°C for 10min, then separated in a Bolt 4-12% Bis-Tris Plus Gel (Thermo Fisher) and transferred onto Trans-Blot Turbo Mini-Size LF-PVDF Membranes (BioRad, USA). The blots were blocked with 5% BSA in Tris-buffered saline+0.1% Tween-20 for 1h, followed by overnight incubation with the primary antibody against the un-phosphorylated Kinase in TBS-T+5%BSA. After washing with TBS-T, blots were incubated with anti-mouse and rabbit HRP (horseradish peroxidase)-conjugated secondary antibody and visualized using Enhanced Chemiluminescence (Pierce Fast Western Blot Kit ECL Substrate, Thermo Fisher, USA) in the Intas ECL Chemocam Imager (Intas, Germany) using the Intas ChemoStar Imager Software (Intas, Germany). Blots were stripped using Restore Plus Western Blot Stripping Buffer (Thermo Fisher) and incubated overnight with the primary antibody against the phosphorylated Kinase in TBS-T+5%BSA. After washing with TBS-T, blots were again incubated with anti-mouse and rabbit HRP (horseradish peroxidase)-conjugated secondary antibody and visualized using Enhanced Chemiluminescence (Pierce Fast Western Blot Kit ECL Substrate, Thermo Fisher, USA) in the Intas ECL Chemocam Imager (Intas, Germany), using the Intas ChemoStar Imager Software (Intas, Germany). The blots were quantified using Fiji ImageJ Software.

Western Blot for human MYH11: 10μg of protein of each sample was denatured and reduced at 70°C for 10min, then separated in a NuPage 3-8% Tris Acetate-Gel and transferred onto Trans-Blot Turbo Mini-Size LF-PVDF Membranes (BioRad, USA). The blots were blocked with 5% BSA in Tris-buffered saline+0.1%Tween-20 for 1h, followed by overnight incubation with the primary Rabbit Anti-smooth muscle Myosin heavy chain 11 antibody (ab53219, Abcam, United Kingdom) in TBS-T + 5% BSA. After washing with TBS-T, blots were incubated with anti-mouse and rabbit HRP (horseradish peroxidase)-conjugated secondary antibody and visualized using Enhanced Chemiluminescence (Pierce Fast Western Blot Kit ECL Substrate, Thermo Fisher) in the Intas ECL Chemocam Imager (Intas, Germany) using the Intas

ChemoStar Imager Software (Intas, Germany). Blots were stripped using Restore Plus Western Blot Stripping Buffer (Thermo Fisher, USA) and incubated with the loading control primary antibody against ß-Actin (A1978-200µl, Sigma Aldrich, USA) in TBS-T+5% BSA for 1h. After washing with TBS-T, blots were again incubated with anti-mouse and rabbit HRP (horseradish peroxidase)-conjugated secondary antibody and visualized using Enhanced Chemiluminescence (Pierce Fast Western Blot Kit ECL Substrate, Thermo Fisher, USA) in the Intas ECL Chemocam Imager (Intas, Germany), using the Intas ChemoStar Imager Software (Intas, Germany).

Porcine Tissue: Protein-Isolation, Concentration Measurement and WB: AAA tissue of 25mM Lenvatinib-treated (*n*=4) and control-treated pigs (*n*=3) was cut in ~50mg pieces on dry ice. Tissue was homogenized in 200*µ*l Tissue Extraction Reagent I (Thermo Fisher, USA). After homogenization with the Bio-Gen PRO200 Homogenizer and Multi-Gen 7XL Probes (Pro Scientific, USA), samples were centrifuged for 20 min, 14.000 rpm at 4°C and the supernatant was frozen down at -80°C in aliquots of total protein lysate. Total protein concentration was measured using the Pierce BCA Protein Assay Kit (Thermo Fisher) following the manufacturer´s instruction. 15*µ*g of protein of each sample was denatured and reduced at 70°C for 10 minutes, then separated in a NuPage 3-8% Tris Acetate-Gel and transferred onto Trans-Blot Turbo Mini-Size LF-PVDF Membranes (BioRad, USA). The blots were blocked with 5% BSA in Tris-buffered saline+0.1% Tween-20 for 1h, followed by overnight incubation with the primary Rabbit Anti-smooth muscle Myosin heavy chain 11 antibody (ab53219, Abcam, United Kingdom) in TBS-T+5%BSA. After washing with TBS-T, blots were incubated with anti-mouse and rabbit HRP (horseradish peroxidase)-conjugated secondary antibody and visualized using Enhanced Chemiluminescence (Pierce Fast Western Blot Kit ECL Substrate, Thermo Fisher, USA) in the Intas ECL Chemocam Imager (Intas, Germany) using the Intas ChemoStar Imager Software (Intas, Germany). Blots were stripped using Restore Plus Western Blot Stripping Buffer (Thermo Fisher, USA) and incubated with the loading control primary antibody against ß-Tubulin in TBS-T+5%BSA for 1h. After washing with TBS-T, blots were again incubated with anti-mouse and rabbit HRP (horseradish peroxidase)-conjugated secondary antibody and visualized using Enhanced Chemiluminescence (Pierce Fast Western Blot Kit ECL Substrate, Thermo Fisher, USA) in the Intas ECL Chemocam Imager (Intas, Germany), using the Intas ChemoStar Imager Software (Intas, Germany).

Statistics and figure composition:
s. material and methods in the main manuscript

References

1. Busch A, Chernogubova E, Jin H, Meurer F, Eckstein HH, Kim M, et al. Four Surgical Modifications to the Classic Elastase Perfusion Aneurysm Model Enable Haemodynamic Alterations and Extended Elastase Perfusion. *European journal of vascular and endovascular surgery : the official journal of the European Society for Vascular Surgery.* 2018.

2. Gandhi R, Cawthorne C, Craggs LJL, Wright JD, Domarkas J, He P, et al. Cell proliferation detected using [(18)F]FLT PET/CT as an early marker of abdominal aortic aneurysm. *J Nucl Cardiol.* 2019.
3. Wanhainen A, Verzini F, Van Herzeele I, Allaire E, Bown M, Cohnert T, et al. Editor's Choice - European Society for Vascular Surgery (ESVS) 2019 Clinical Practice Guidelines on the Management of Abdominal Aorto-iliac Artery Aneurysms. *European journal of vascular and endovascular surgery : the official journal of the European Society for Vascular Surgery.* 2019;57(1):8-93.
4. Pelisek J, Hegenloh R, Bauer S, Metschl S, Pauli J, Glukha N, et al. Biobanking: Objectives, Requirements, and Future Challenges-Experiences from the Munich Vascular Biobank. *J Clin Med.* 2019;8(2).

Supplement Table I

Ultrasound measurements

experiment	Day 0		Day 7		Day 14		Day 28		
	absolu	norma	absolu	norma	absolut	norma	absolu	normalized	
stan PPE 1	0,58	1,00	0,93	1,59	0,85	1,47	0,98	1,69	stan PPE N=11
stan PPE 2	0,55	1,00	0,90	1,64	0,86	1,56	0,89	1,61	
stan PPE 3	0,62	1,00	0,87	1,39	0,87	1,39	1,00	1,60	
stan PPE 4	0,62	1,00	0,87	1,40	0,93	1,50	1,07	1,73	
stan PPE 5	0,56	1,00	0,76	1,36	0,83	1,48	0,93	1,66	
stan PPE 6	0,55	1,00	0,83	1,51	0,99	1,80	0,99	1,80	
stan PPE 7	0,62	1,00	0,80	1,29	1,03	1,66	1,10	1,77	
stan PPE 8	0,60	1,00	0,79	1,32	0,95	1,59	1,03	1,71	
stan PPE 9	0,59	1,00	0,86	1,45	0,99	1,67	1,04	1,75	
stan PPE 10	0,53	1,00	0,81	1,52	0,91	1,71	0,98	1,83	
stan PPE 11	0,61	1,00	0,87	1,43	0,93	1,53	1,17	1,93	
	Day 0		Day 7		Day 14		Day 28		
	absolu	rmaliz	absolu	rmaliz	absolut	rmaliz	absolu	rmalized	
mean	**0,59**	**1,00**	**0,84**	**1,45**	**0,92**	**1,58**	**1,01**	**1,73**	
standard dev	0,03	0,00	0,05	0,10	0,06	0,12	0,08	0,09	
Lenva syst	0,58	1,00	0,85	1,45	0,89	1,53	0,86	1,48	Lenva syst N=7
Lenva syst2	0,61	1,00	0,75	1,23	0,94	1,54	0,95	1,55	
Lenva syst	0,63	1,00	0,87	1,39	0,80	1,27	0,94	1,50	
Lenva syst	0,59	1,00	0,70	1,18	0,78	1,31	0,67	1,13	
Lenva syst	0,56	1,00	0,80	1,43	0,77	1,38	0,81	1,44	
Lenva syst	0,61	1,00	0,78	1,28	0,82	1,35	0,87	1,43	
Lenva syst	0,65	1,00	0,81	1,24	0,71	1,09	0,73	1,12	
	Day 0		Day 7		Day 14		Day 28		
	absolu	rmaliz	absolu	rmaliz	absolut	rmaliz	absolu	rmalized	
mean	**0,60**	**1,00**	**0,79**	**1,32**	**0,81**	**1,35**	**0,83**	**1,38**	
standard dev	0,03	0,00	0,05	0,10	0,07	0,14	0,10	0,16	
Sham-Re O	0,52	1,00	0,84	1,61	0,93	1,80	1,04	2,01	Sham-Re OP N=3
Sham-Re O	0,58	1,00	0,83	1,44	0,92	1,59	0,88	1,52	
Sham-Re O	0,49	1,00	0,76	1,55	0,82	1,66	0,89	1,80	
mean	**0,53**	**1,00**	**0,81**	**1,53**	**0,89**	**1,68**	**0,94**	**1,78**	
standard dev	0,03	0,00	0,03	0,07	0,05	0,09	0,08	0,20	

Lenva local	0,58	1,00	0,75	1,29	0,79	1,37	0,75	1,29	Lenva local N=5
Lenva local	0,53	1,00	0,83	1,57					
Lenva local	0,61	1,00	0,88	1,45	0,78	1,28	0,90	1,48	
Lenva local	0,63	1,00	0,83	1,33	0,79	1,26	0,89	1,41	
Lenva local	0,47	1,00	0,78	1,66	0,88	1,87	0,75	1,60	
**mean**	**0,56**	**1,00**	**0,81**	**1,46**	**0,81**	**1,44**	**0,82**	**1,45**	
standard dev	0,06	0,00	0,05	0,14	0,04	0,25	0,07	0,11	

Laboratory Analysis

stan PPE 4	4,4	3,4	0,1	0,9	727	8,6
stan PPE 5	3,6	2,9	0,1	0,6	818	8,6
stan PPE 6	2,3	1,7	0,1	0,5	350	10,6
stan PPE 7	3,9	2,9	0,1	0,9	576	9,4
**mean**	**3,55**	**2,73**	**0,10**	**0,73**	**617,75**	**9,3**
**standard dev**	**0,78**	**0,63**	**0,00**	**0,18**	**177,11**	**0,82**

	**white cell count**	**monocytes**	**granulocytes**
normal range	**3.0-15.0 x10^9/µl**	**0.3-0.8 x10^9/µl**	**1.2-6.8 x10^9/µl**
	**platelets**	**hemoglobin**	**lymphocytes**
	**140-600 x10^9/µl**	**6.9-11,2mmol/l**	**1.2-3.2 x10^9/µl**

Lenva syst [illegible]	6,3	4,2	0,2	1,9	637	9,6
Lenva syst2	4,6	3,1	0,2	1,3	937	9,2
Lenva syst [illegible]	4,2	2,9	0,1	1,2	798	9,2
Lenva syst [illegible]	8,8	6,2	0,3	2,3	669	9,9
Lenva syst [illegible]	4,7	3,5	0,2	1	835	9,1
Lenva syst [illegible]	4,7	3,7	0,1	0,9	855	8,8
Lenva syst [illegible]	4,4	3,3	0,1	1	788	9,5
**mean**	**5,39**	**3,84**	**0,17**	**1,37**	**788,43**	**9,33**
**standard dev**	**1,53**	**1,04**	**0,07**	**0,49**	**97,07**	**0,34**

Angotension II model

diameter absolute

		baselin	d3	d7	d14	d21	d27
2060	PBS	1,087	1,396	2,010	2,224	2,447	2,444
2061	PBS	1,072	1,428	1,183	1,409	1,900	1,446
2053	PBS	1,240	1,517	1,663	1,657	1,790	1,688
2067	PBS	1,190	1,821	2,373	2,792	2,580	2,610
2068	PBS	1,215	1,388	2,007	2,086	2,049	2,355
2054	Lenva	1,252	1,339	2,080	2,267	2,079	3,126
2058	Lenva	1,149	1,559	1,530	1,555	1,907	1,760
2062	Lenva	1,147	1,519	1,579	1,415	1,597	1,463
2063	Lenva	1,037	1,685	1,443	1,891	1,774	1,847
2065	Lenva	1,079	1,453	1,383	1,544	1,327	2,139
2056	Lenva	1,063	1,294	1,426	1,366	1,556	1,426

Volume abs

2060	PBS	7,97	14,25	25,63	29,82	32,83	32,93
2061	PBS	8,42	11,18	9,66	12,36	15,79	10,36
2053	PBS	10,41	14,84	20,05	16,95	14,73	17,42
2067	PBS	9,06	19,03	33,41	28,72	33,09	29,69
2068	PBS	7,70	12,12	28,12	28,56	32,75	33,81
2054	Lenva	10,54	12,00	25,78	29,54	26,08	49,04
2058	Lenva	8,08	14,11	19,18	17,73	19,87	17,06
2062	Lenva	8,61	16,20	15,75	15,18	13,61	13,37
2063	Lenva	7,04	15,65	16,16	18,23	19,10	19,98
2065	Lenva	7,86	13,86	15,04	14,26	13,67	25,97
2056	Lenva	8,64	11,03	16,06	17,84	16,02	14,35

Local Mouse Data

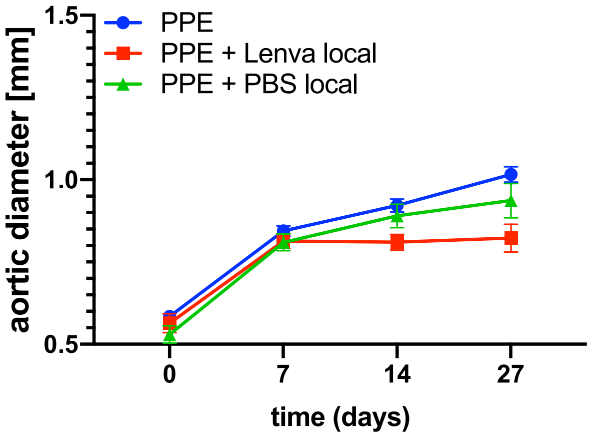

	Significant?	P value	Mean of PPE	Mean of PPE + Lenva local	Difference	SE of difference	t ratio	df	Adjusted P Value
0	No	0.403659	0.5845	0.5640	0.02055	0.02386	0.8612	14.00	0.487498
7	No	0.284107	0.8445	0.8140	0.03055	0.02742	1.114	14.00	0.487498
14	Yes	0.008020	0.9218	0.8100	0.1118	0.03576	3.127	13.00	0.023868
27	Yes	0.001095	1.016	0.8225	0.1939	0.04646	4.172	13.00	0.004372

	Significant?	P value	Mean of PPE	Mean of PPE + PBS local	Difference	SE of difference	t ratio	df	Adjusted P Value
0	No	0.034843	0.5845	0.5300	0.05455	0.02293	2.379	12.00	0.132254
7	No	0.308702	0.8445	0.8100	0.03455	0.03250	1.063	12.00	0.522107
14	No	0.461861	0.9218	0.8900	0.03182	0.04186	0.7601	12.00	0.522107
27	No	0.153341	1.016	0.9367	0.07970	0.05228	1.524	12.00	0.393088

Data shown as Mean +/- SEM. Multiple t-test (1 per row) with *post hoc* Holm-Sidak correction for multiple comparisons. n = 11 (PPE) , 5 (Lenva local) or 3 (PBS local)

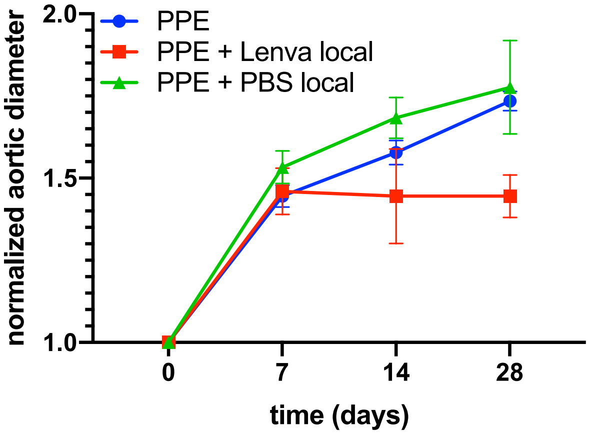

	Significant?	P value	Mean of PPE	Mean of PPE + Lenva local	Difference	SE of difference	t ratio	df	Adjusted P Value
0			1.000	1.000	0.000	0.000			
7	No	0.832420	1.445	1.460	-0.01455	0.06747	0.2156	14.00	0.832420
14	No	0.213376	1.578	1.445	0.1332	0.1018	1.308	13.00	0.381222
28	Yes	0.000423	1.735	1.445	0.2895	0.06174	4.690	13.00	0.001269

	Significant?	P value	Mean of PPE	Mean of PPE + PBS local	Difference	SE of difference	t ratio	df	Adjusted P Value
0			1.000	1.000	0.000	0.000			
7	No	0.228973	1.445	1.533	-0.08788	0.06932	1.268	12.00	0.488461
14	No	0.200240	1.578	1.683	-0.1052	0.07758	1.355	12.00	0.488461
28	No	0.638269	1.735	1.777	-0.04212	0.08733	0.4823	12.00	0.638269

Data shown as Mean +/- SEM. Multiple t-test (1 per row) with *post hoc* Holm-Sidak correction for multiple comparisons. n = 11 (PPE) , 5 (Lenva local) or 3 (PBS local)

Mouse Data

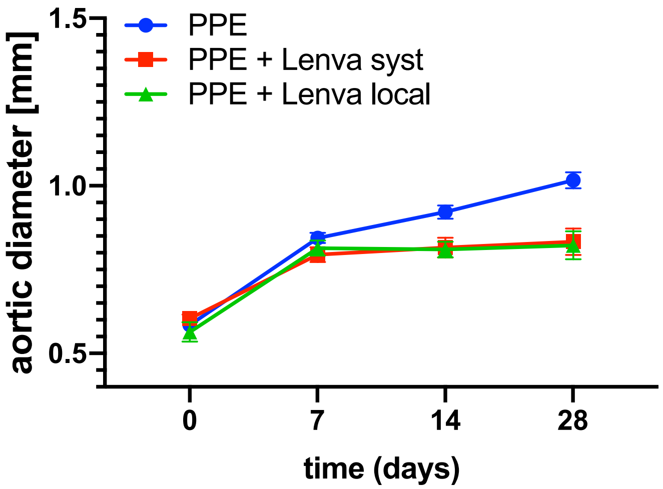

	Significant?	P value	Mean of PPE	Mean of PPE + Lenva syst	Difference	SE of difference	t ratio	df	Adjusted P Value
0	No	0.218325	0.5845	0.6043	-0.01974	0.01541	1.281	16.00	0.218325
7	No	0.071058	0.8445	0.7943	0.05026	0.02599	1.934	16.00	0.137067
14	Yes	0.006269	0.9218	0.8157	0.1061	0.03374	3.144	16.00	0.018689
28	Yes	0.000582	1.016	0.8329	0.1835	0.04294	4.274	16.00	0.002325

	Significant?	P value	Mean of PPE	Mean of PPE + Lenva local	Difference	SE of difference	t ratio	df	Adjusted P Value
0	No	0.403659	0.5845	0.5640	0.02055	0.02386	0.8612	14.00	0.487498
7	No	0.284107	0.8445	0.8140	0.03055	0.02742	1.114	14.00	0.487498
14	Yes	0.008020	0.9218	0.8100	0.1118	0.03576	3.127	13.00	0.023868
28	Yes	0.001095	1.016	0.8225	0.1939	0.04646	4.172	13.00	0.004372

Data shown as Mean +/- SEM. Multiple t-test (1 per row) with *post hoc* Holm-Sidak correction for multiple comparisons. n = 11 (PPE) , 7 (Lenva systemic) or 5 (Lenva local)

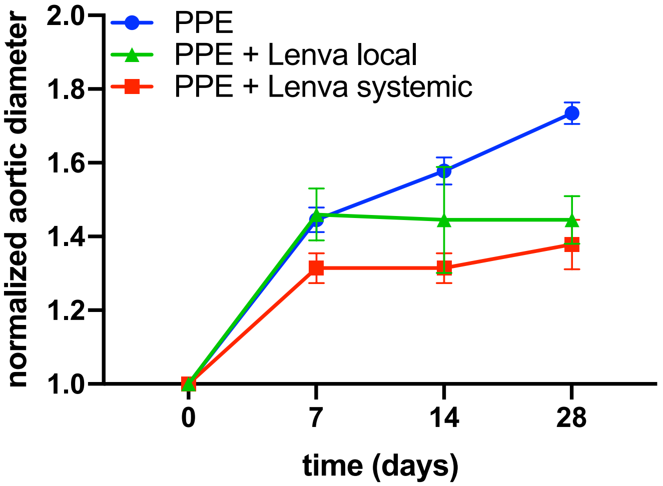

	Significant?	P value	Mean of PPE	Mean of PPE + Lenva syst	Difference	SE of difference	t ratio	df	Adjusted P Value
0			1.000	1.000	0.000	0.000			
7	Yes	0.024318	1.445	1.314	0.1312	0.05275	2.487	16.00	0.024318
14	Yes	0.000246	1.578	1.314	0.2639	0.05627	4.690	16.00	0.000492
28	Yes	0.000046	1.735	1.379	0.3560	0.06446	5.523	16.00	0.000139

	Significant?	P value	Mean of PPE	Mean of PPE + Lenva local	Difference	SE of difference	t ratio	df	Adjusted P Value
0			1.000	1.000	0.000	0.000			
7	No	0.832420	1.445	1.460	-0.01455	0.06747	0.2156	14.00	0.832420
14	No	0.213376	1.578	1.445	0.1332	0.1018	1.308	13.00	0.381222
28	Yes	0.000423	1.735	1.445	0.2895	0.06174	4.690	13.00	0.001269

Data shown as Mean +/- SEM. Multiple t-test (1 per row) with *post hoc* Holm-Sidak correction for multiple comparisons. n = 11 (PPE) , 7 (Lenva systemic) or 5 (Lenva local)

AngII + Gavage Data - Diameter

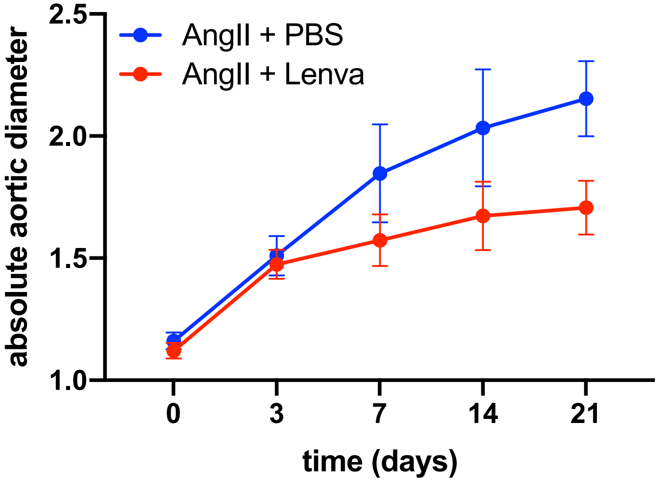

	Below threshold?	P value	Mean of AngII + PBS	Mean of AngII + Lenva	Difference	SE of difference	t ratio	df	Adjusted P Value
0	No	0.420840	1.161	1.121	0.03963	0.04699	0.8434	9.000	0.664573
3	No	0.728246	1.510	1.475	0.03517	0.09810	0.3585	9.000	0.728246
7	No	0.235388	1.847	1.574	0.2737	0.2152	1.272	9.000	0.607306
14	No	0.208386	2.034	1.673	0.3606	0.2661	1.355	9.000	0.607306
21	No	0.038779	2.153	1.707	0.4465	0.1847	2.417	9.000	0.179429

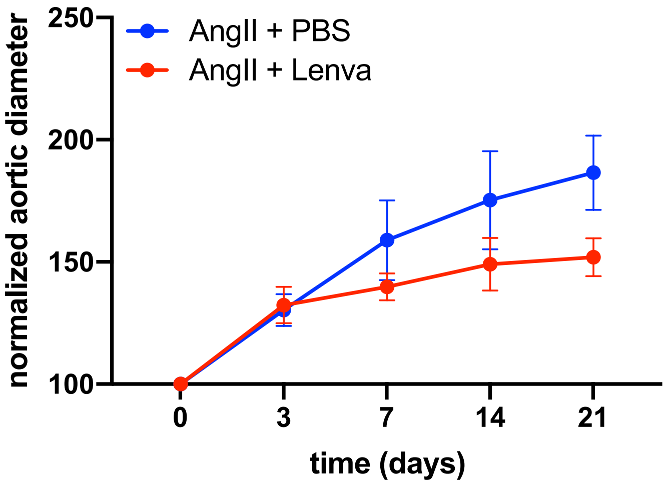

	Below threshold?	P value	Mean of AngII + PBS	Mean of AngII + Lenva	Difference	SE of difference	t ratio	df	Adjusted P Value
0			100.0	100.0	0.000	0.000			
3	No	0.843013	130.3	132.4	-2.064	10.13	0.2038	9.000	0.843013
7	No	0.262617	158.9	139.8	19.07	15.96	1.195	9.000	0.591034
14	No	0.257729	175.3	149.1	26.19	21.68	1.208	9.000	0.591034
21	No	0.060732	186.5	152.0	34.55	16.12	2.143	9.000	0.221680

AngII + Gavage Data - Volume

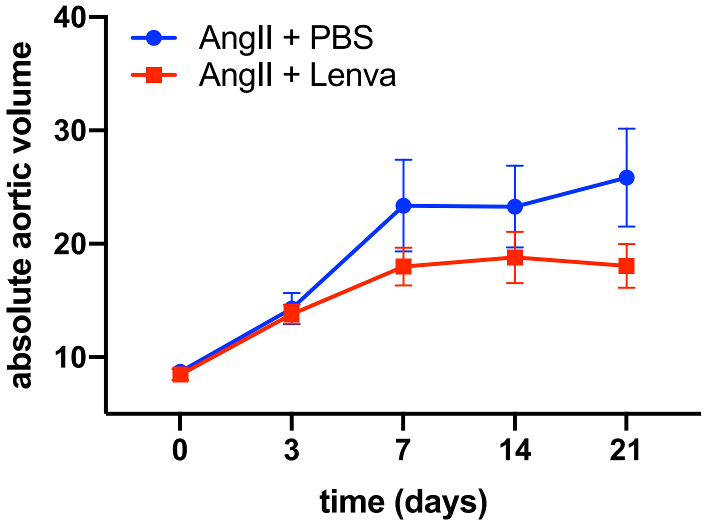

	Below threshold?	P value	Mean of AngII + PBS	Mean of AngII + Lenva	Difference	SE of difference	t ratio	df	Adjusted P Value
0	No	0.723776	8.712	8.462	0.2503	0.6865	0.3647	9.000	0.923700
3	No	0.762740	14.28	13.81	0.4757	1.529	0.3112	9.000	0.923700
7	No	0.220849	23.37	18.00	5.379	4.089	1.316	9.000	0.631458
14	No	0.301495	23.28	18.80	4.485	4.092	1.096	9.000	0.659193
21	No	0.114185	25.84	18.06	7.780	4.448	1.749	9.000	0.454599

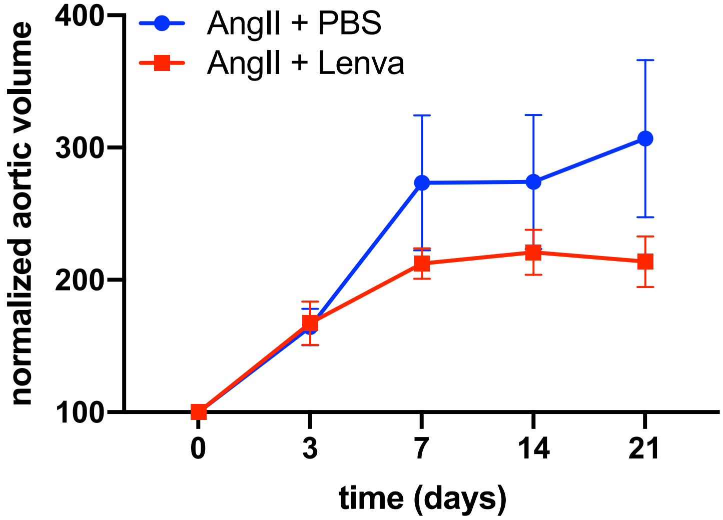

	Below threshold?	P value	Mean of AngII + PBS	Mean of AngII + Lenva	Difference	SE of difference	t ratio	df	Adjusted P Value
0			100.0	100.0	0.000	0.000			
3	No	0.895642	164.4	167.3	-2.955	21.90	0.1349	9.000	0.895642
7	No	0.233026	273.5	212.4	61.05	47.75	1.279	9.000	0.548828
14	No	0.309926	274.0	220.9	53.10	49.35	1.076	9.000	0.548828
21	No	0.141198	306.8	213.8	92.99	57.65	1.613	9.000	0.456034

For the PPE data analysis, the adjustment was performed for comparisons deemed relevant as per below:

- D0+7 comparisons – not relevant
- D14+28 comparisons – relevant (PPE vs Lenva syst, PPE vs Lenva local, Lenva local vs Sham reop), not relevant (remaining 3 permutations)
- D14 + D28 vs D7 for each treatment.

Thus, the correction for 3 tests was performed for treatment comparisons and for 2 tests for time.

Comparison (absolute)	p-value	Sidak adj-p
D14 - PPE vs. Lenva syst	0.0009	0.002697571 **
D14 - PPE vs. Lenva local	0.0036	0.010761167 **
D14 - Lenva local vs. Sham reop	0.1075	0.289073547
D28 - PPE vs. Lenva syst	<0.0001	<0.0001 ***
D28 - PPE vs. Lenva local	<0.0001	<0.0001 ***
D28 - Lenva local vs. Sham reop	0.0207	0.0608234
PPE – d14 vs d7	0.0173	0.03430071 *
PPE – d28 vs d7	0.0001	0.00019999 ***
Sham reop – d14 vs d7	0.0153	0.03036591 *
Sham reop – d28 vs d7	0.0998	0.18963996
Lenva syst – d14 vs d7	0.5935	0.8347578
Lenva syst – d28 vs d7	0.3105	0.5245898
Lenva local – d14 vs d7	0.9877	0.9998487
Lenva local – d28 vs d7	0.5974	0.8379132

Table

Results table for ANOVA

Source of Variation	% of total variation	P value	SS	DF	MS	F (DFn, DFd)
Time x Treatment	5.596	0.0001	0.1308	9	0.01453	F (9, 63) = 4.614
Time	54.4	<0.0001	1.271	3	0.4238	F (2.552, 53.58) = 134.5
Treatment	6.572	0.0017	0.1536	3	0.05120	F (3, 21) = 7.181
Subject	6.405	0.0067	0.1497	21	0.007129	F (21, 63) = 2.263
Residual			0.1985	63	0.003150	

Assumption of normality:

Groupwise residual plots

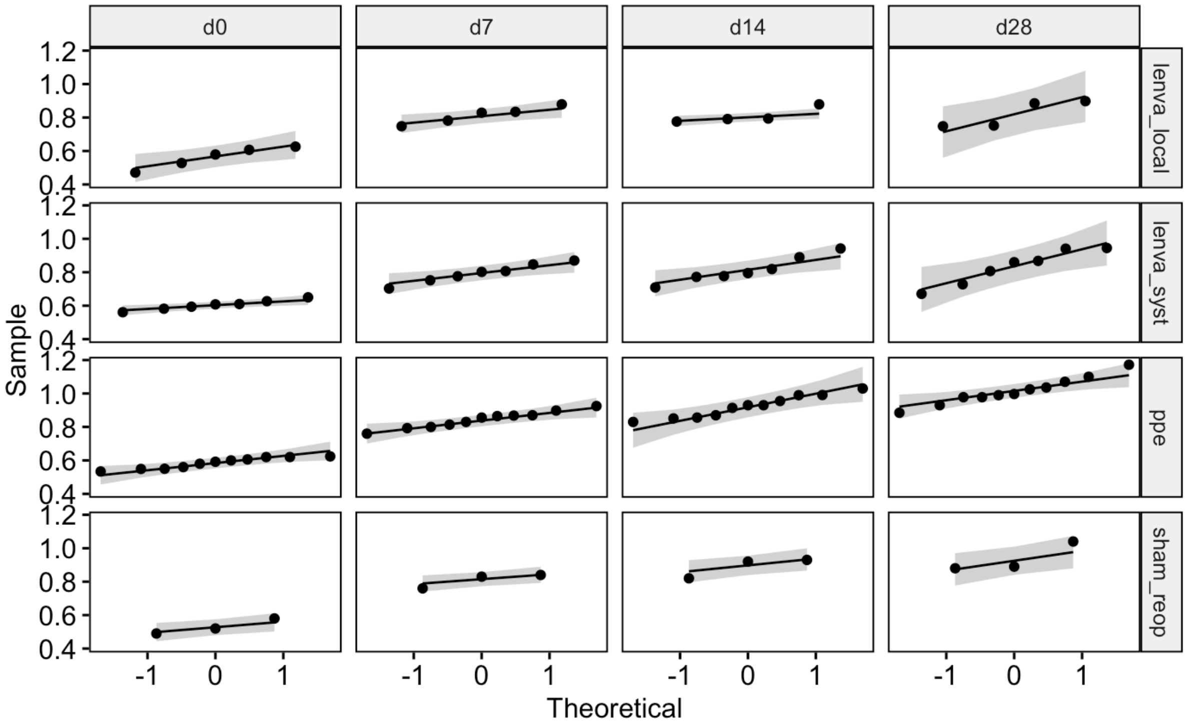

Shapiro-Wilk’s test **NS** for all above.

Overall residual plot

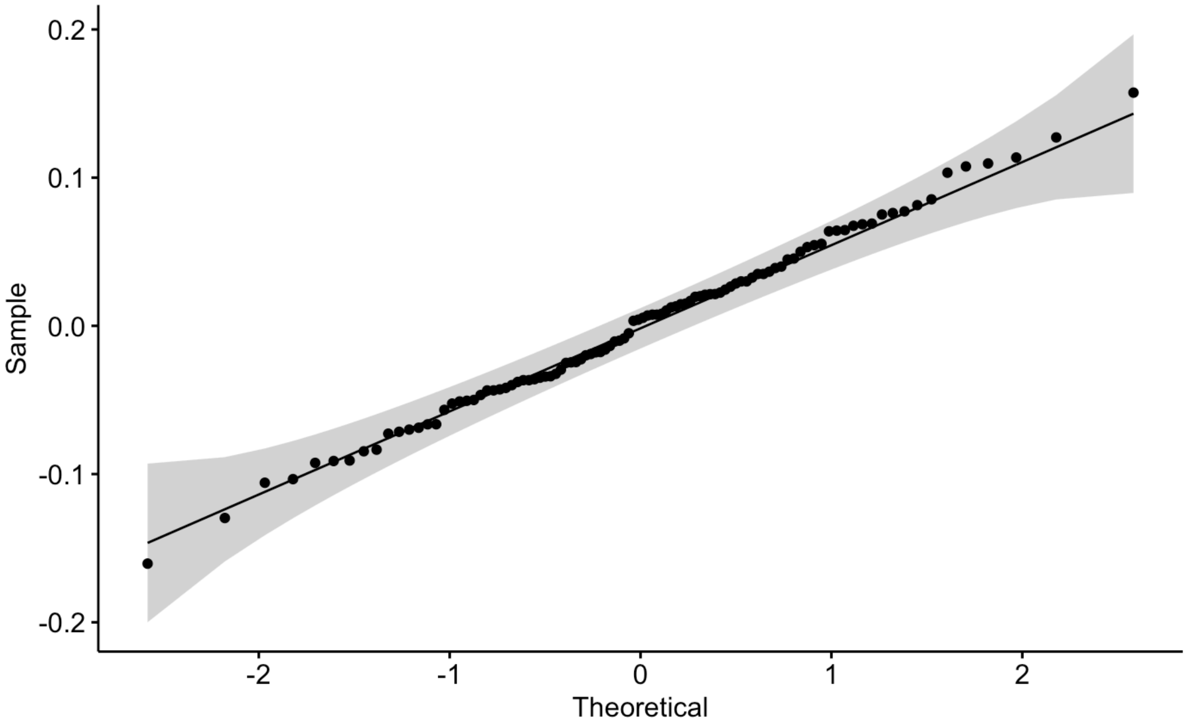

Shapiro-Wilk’s test for all datapoints – NS (p=0.997)

Assumption of homogeneity of variance:

Levene's test – NS:

timepoint	df1	df2	statistic	p
d0	3	22	1.133	0.3574
d7	3	22	0.1569	0.9241
d14	3	21	0.483	0.6977
d28	3	21	0.2249	0.878
Overall	15	86	0.9837	0.4792

Box's M-test for Homogeneity of covariance – NS (p=0.116)

Supplement Table II: Primer List

target	name	sequence/name	supplier
**TAGLN**	transgelin	Hs01038777_g1	Thermo Fisher
change and transformation sensitive actin-binding protein; belongs to calponin family; ubiquitously expressed in vascular smooth muscle; early marker of smooth muscle differentiation			
**CNN1**	calponin 1	Hs00959434_m1	Thermo Fisher
an actin filament-associated regulatory protein; role in contractile functions;			
**MYOCD**	myocardin	Hs00538076_m1	Thermo Fisher
nuclear protein, expressed in heart, aorta, and smooth muscle cell-containing tissues; modulates expression of smooth muscle-specific SRF-target genes; crucial role in differentiation of the smooth muscle cell lineage			
**ITGA8**	integrin subunit alpa 8	Hs00233321_m1	Thermo Fisher
Integrins are heterodimeric transmembrane receptor proteins that mediate numerous cellular processes; the integrin alpha8beta1 protein thus plays an important role in wound-healing and organogenesis;			
**COL3A1**	collagen type III alpha 1 chain	Hs00943809_m1	Thermo Fisher
fibrillar collagen extensible in connective tissues such as the vascular system; Mutations are associated with Ehlers-Danlos syndrome types IV and with aortic and arterial aneurysms			
**CALD1**	caldesmon 1	Hs00921987_m1	Thermo Fisher
calmodulin- and actin-binding protein that plays an essential role in the regulation of smooth muscle and nonmuscle contraction			
**S100A1**	S100 calcium binding protein A1	Hs00984741_m1	Thermo Fisher
S100 proteins are localized in the cytoplasm and/or nucleus of a wide range of cells and involved in the regulation of a number of cellular processes such as cell cycle progression and differentiation			
**KDR**	kinase insert domain receptor	Hs00911700_m1	Thermo Fisher
VEGF receptor 2 functions as the main mediator of VEGF-induced endothelial proliferation, survival, migration, tubular morphogenesis and sprouting			
**RPLP0**	ribosomal protein lateral stalk subunit P0	HS00420895_gH	Thermo Fisher
housekeeping gene for PCR			
**VCAN**	versican	HS00171642_M1	Thermo Fisher
aggrecan/versican proteoglycan family; major component of the extracellular matrix; nvolved in cell adhesion, proliferation, proliferation, migration and angiogenesis;			
**HIF1a**	hypoxia inducible factor 1 subunit alpha	Hs_00153153_m1	Thermo Fisher
HIF-1 functions as a master regulator of cellular and systemic homeostatic response to hypoxia; essential role in embryonic vascularization, tumor angiogenesis and ischemic disease;			
**ACTA2**	actin alpha 2	HS00426835_G1	Thermo Fisher
smooth muscle actin that is involved in vascular contractility;			
**GAPDH**	glyceraldehyde-3-phosphate dehydrogenase	HS03929097_G1	Thermo Fisher
housekeeping gene for PCR			
**SMTN**	smoothelin	Hs01022255_g1	Thermo Fisher
structural protein that is found exclusively in contractile smooth muscle cells;			
**MMP2**	matrix metallopeptidase 2	Hs_01548727	Thermo Fisher
zinc-dependent enzymes capable of cleaving components of the extracellular matrix; type IV collagenase;			
**GAPDH**	s. above	Hs_GAPDH_1_SG	Qiagen
housekeeping gene for PCR in pig tissues			
**MYH11**_human_fwd	myosin heavy chain 11	TCGAAGAAGAAGCTGCAGGA	Thermo Fisher
MYH11_human_rev		GTACTGCTGGGTGAGGTTCT	Thermo Fisher
smooth muscle myosin; major contractile protein			
**RPLP0**_human_fwd	s. above	ATGGCAGCATCTACAACCCT	Thermo Fisher
RPLP0_human_rev		TTGGGTAGCCAATCTGCAGA	Thermo Fisher
housekeeping gene for PCR in pig tissues			

Supplement Table III

Ultrasound measurements								
experimei	sex	Day O		Day 7		Day 28		
		absolut	normali	absolut	normali	absolut	normali	
								N=7
stan PPE	male	8,59	1			17,19	2,00	
stan PPE	female	6,63	1	9,50	1,43	12,70	1,92	
stan PPE	female	5,60	1	8,20	1,46	8,60	1,54	
stan PPE	male	6,80	1	8,60	1,26	10,60	1,56	
stan PPE	female	6,63	1	9,50	1,43	10,40	1,57	
stan PPE	female	6,40	1	7,60	1,19	9,60	1,50	
stan PPE	male	7,20	1	8,60	1,19	12,00	1,67	
mean		**7,56**	**1**	**9,48**	**1,21**	**11,78**	**1,56**	
tandard dev		1,34	0	1,07	0,15	2,40	0,21	
Lenva 1	male	7,10	1	10,00	1,41	9,70	1,37	N=4
Lenva 2	female	8,00	1	10,80	1,35	9,70	1,21	
Lenva 3	female	8,50	1	10,50	1,24	9,90	1,16	
Lenva 4	male	8,10	1	10,00	1,23	9,40	1,16	
mean		7,79	1	10,68	1,38	9,68	1,23	
tandard dev		0,56	0	0,89	0,13	0,18	0,08	
DMSO 9	female	7,70	1	11,00	1,43	11,30	1,47	N=3
DMSO 11	female	7,90	1	12,20	1,54	10,60	1,34	
DMSO 12	female	8,10	1	10,40	1,28	10,80	1,33	
mean		7,79	1	10,68	1,38	10,90	1,38	
tandard dev		0,56	0	0,89	0,13	0,29	0,06	
wt	male	8,2	1	9,8	1,195	11,6	1,415	N=3
wt	male	7,2	1	8,6	1,194	12	1,667	
wt	female	7	1	9,3	1,329	10,1	1,443	
mean		**7,47**	**1,00**	**9,23**	**1,24**	**11,23**	**1,51**	
tandard dev		**0,52**	**0,00**	**0,49**	**0,06**	**0,82**	**0,11**	

experimen	sex	Day O		Day 7		Day 28	
		absolut	normali	absolut	normali	absolut	normalized
stan PPE	male	8,59	1			17,19	2,00
stan PPE	female	6,63	1	9,50	1,43	12,70	1,92
stan PPE	female	5,60	1	8,20	1,46	8,60	1,54
stan PPE	male	6,80	1	8,60	1,26	10,60	1,56
stan PPE	female	6,63	1	9,50	1,43	10,40	1,57
stan PPE	female	6,40	1	7,60	1,19	9,60	1,50
stan PPE	male	7,20	1	8,60	1,19	12,00	1,67
**mean**	**male**	**7,53**	**1,00**	**8,60**	**1,23**	**13,26**	**1,74**
**tandard dev**		0,77	0,00	0,00	0,04	2,83	0,19
**mean**	**female**	**6,32**	**1,00**	**8,70**	**1,38**	**10,33**	**1,63**
**tandard dev**		0,42	0,00	0,83	0,11	1,51	0,17

Laboratory analysis								
		Day 0						
Animal		1	2	3	4	9	11	12
treatment		Lenva	Lenva	Lenva	Lenva	DMSO	DMSO	DMSO
**Blood cou**	**normal range**							
WBC (G/L)	11,0-22,0	6,7	7,7	5,9	6,7	15	7,2	8
RBC (T/L)	5,8-6,1	5,89	5,11	6,3	5,89	4,76	6,18	5,57
HGB (g/L)	120-160	123	105	133	123	106	135	117
HCT (L/L)	0,36-0,50	0,38	0,32	0,38	0,38	0,33	0,42	0,36
MCV (fL)	50-65	64,7	63,1	60,7	64,7	68,9	67,5	65,2
MCH (pg)	17-21	20,9	20,5	21,1	20,9	22,3	21,8	21
MCHC (g/(	30-35	32,3	32,6	34,8	32,3	32,3	32,4	32,2
PLT (G/L)	220-620	334	370	289	334	301	202	156
**Liver**								
alcalic phc	< 156	56	78	48	56	73	46	114
AST (U/L)	< 59	32	67	29	32	29	31	22
ALT (U/L)	< 68	31	56	31	31	20	43	31
y-GT	< 54	71	69	76	71	55	58	65
GLDH (U/L	-6,4	< 2,0	5,4	< 2,0	<2,0	<2,0	<2,0	<2,2
Bilirubin (μ	< 4,3	6,84	3,42	3,42	6,84	11,97	5,13	8,55
CK (U/L)	< 4000	390	375	410	390	361	361	177
**Kidney**								
creatinine	< 160	147,6	116,7	101,7	147,6	157,4	123,8	115,8
urea (mm(	2,5-6,7	4,00	4,66	2,33	4	9,49	4,66	2,83
sodium (m	135-150	144	142	139	144	144	140	141
potassium	4,2-5,6	3,8	3,9	4,3	3,8	3,1	4,1	4
Calcium(m	1,80-2,90	2,49	2,4	2,44	2,49	2,52	2,52	2,52
Magnesiui	0,50-1,30	0,75	0,84	0,79	0,75	0,88	0,78	0,76
chloride (r	102-106	101	101	101	101	99	99	99
Phosphat (	1,90-3,20	1,94	1,97	1,86	1,94	2,05	2,15	2,04
**other**								
total iron (	> 18	28,7	14	19,9	28,7	31,2	29,6	20,2
glucose (m	4,05-6,60	10,38	7,16	10,16	10,38	5	9,99	9,32
Albumin(g	28-40	33,9	30,2	33,5	33,9	36,6	32,9	33,4
total prote	65-85	79	72	74	79	78	80	80

Day 7						
1 Lenva	2 Lenva	3 Lenva	4 Lenva	9 DMSO	11 DMSO	12 DMSO
9,2	9,1	10,5	7,7	9,8	9,6	7,6
3,08	4,67	4,04	3,47	4,02	4,68	5,31
70	96	84	74	89	102	110
0,21	0,3	0,25	0,23	0,28	0,31	0,34
68,3	64	61,7	66	68,6	64,6	64,8
22,7	20,6	20,8	21,3	22,1	21,8	20,7
33,3	32,1	33,7	32,3	32,3	33,2	32
542	346	316	536	151	223	83
62	47	33	35	59	35	73
34	35	29	33	37	40	26
31	35	30	34	33	34	31
53	75	34	59	50	65	52
15,6	15,9	<2,0	15,8	<2,0	<2,0	<2,0
5,13	5,13	5,13	6,84	6,84	6,84	5,13
692	649	496	613	505	511	536
96,4	122	105,2	120,2	118,5	146,7	107,9
4,5	3,16	2,16	4	2,33	4,16	3,5
142	144	143	145	141	138	140
4	3,9	3,2	3,8	3,3	3,5	4
2,45	2,38	2,32	2,39	2,46	2,5	2,54
0,98	0,86	0,78	0,93	0,9	1,05	0,97
103	102	101	102	98	100	101
2,23	1,94	1,87	2,15	2,3	2,09	2,09
21,5	10,6	12	27,4	16,8	16,3	19,3
4	4,22	5,44	5,44	7,16	7,38	11,1
31,7	25,4	23,7	27,4	32,1	30	30,8
64	63	54	63	72	72	69

Day 28						
**1**	**2**	**3**	**4**	**9**	**11**	**12**
**Lenva**	**Lenva**	**Lenva**	**Lenva**	**DMSO**	**DMSO**	DMSO
5,8	7	6,3	5,8	6,7	6,3	8,4
5,32	6	5,71	5,32	5,03	6,13	5,1
116	126	118	116	110	134	112
0,36	0,39	0,37	0,36	0,33	0,4	0,34
67,5	64,8	64,9	67,5	65,8	64,7	66,2
21,8	21	20,7	21,8	21,9	21,9	22
32,3	32,4	31,8	32,3	33,2	33,8	33,2
431	263	256	431	415	298	403
64	61	61	64	105	67	103
31	29	33	31	33	28	12
34	41	33	34	29	42	29
56	63	48	56	49	43	44
< 2,0	< 2,0	< 2,0	< 2,0	< 2,0	< 2,0	< 2,0
3,42	3,42	3,42	3,42	3,42	3,42	5,13
120	157	339	120	185	124	56
91,1	122,9	108,7	91,1	97,2	112,3	103,4
4,83	4	3,66	4,83	3	3,83	2,33
143	141	146	143	140	139	139
3,6	3,6	3,3	3,6	3,5	4	3,3
2,5	2,57	2,45	2,5	2,51	2,48	2,55
0,76	0,76	0,83	0,76	0,71	0,79	0,83
103	100	103	103	103	104	99
1,9	1,67	1,84	1,9	1,91	1,87	1,79
42,8	18,3	22,7	42,8	25,4	21,9	9,1
4,83	6,83	6,6	4,83	4,66	7,16	9,82
34,7	33,4	31,7	34,7	35,9	33	34,8
79	82	73	79	75	76	78

Supplement Table IV: Antibody List

target	used in	antibody	manufacturer	dilution
**mouse mouse mouse mouse mouse mouse mouse mouse mouse mouse**				
KI67	IHC	ab16667	Abcam	1:50
MYH11	IHC	ab224804	Abcam	1:100
α smooth muscle actin	IHC	ab119952	Abcam	1:200
CD31	IHC	ab28364	Abcam	1:200
**pig and human pig and human pig and human pig and human pig and human**				
SMA	IHC<br>prim IF	ab7817	Abcam	1:200
CD31	IHC	ab28364	Abcam	1:500
MYH11	IHC<br>prim IF<br>WB	ab53219	Abcam	1:300<br>1:200<br>1:500
Alexa Fluor 488	sec IF<br>human/pig	A11034	Invitrogen	1:300 (human)<br>1:500 (pig)
Alexa Fluor 594	sec IF<br>pig	A11032	Invitrogen	1:500
Alexa Fluor 647	sec IF<br>human	A21236	Invitrogen	1:300
ß-actin	WB	A1978	Sigma-Aldrich	1:5000
Phospho-p44/42 MAPK (Erk1/2)	WB	4370	Cell Signaling	1:2000
p44/42 MAPK (Erk1/2)	WB	4695	Cell Signaling	1:1000
Phospho-SAPK/JNK	WB	9255	Cell Signaling	1:2000
Phospho-Akt	WB	9271	Cell Signaling	1:1000
Akt	WB	9272	Cell Signaling	1:1000
ß-Tubulin	WB	Ab6046	Abcam	1:500
Goat Anti-Mouse IgG H&L (HRP)	WB	ab205719	Abcam	1:10000
Goat Anti-Rabbit IgG H&L (HRP)	WB	ab205718	Abcam	1:2000
